# Deep learning recognises antibiotic modes of action from brightfield images

**DOI:** 10.1038/s41467-026-76355-0

**Published:** 2026-09-08

**Authors:** Daniel Krentzel, Kelvin Kho, Julienne Petit, Nassim Mahtal, Thomas Delerue, Max E. Huber, Agnès Zettor, Jeanne Chiaravalli, Spencer L. Shorte, Mark Brönstrup, Anne Marie Wehenkel, Ivo G. Boneca, Christophe Zimmer

**Affiliations:** 1 Imaging and Modeling Unit, Institut Pasteur, Université Paris Cité, Paris, France; 2 Unité de Biologie et Génétique de la Paroi Bactérienne, CNRS UMR6047, INSERM U1306, Institut Pasteur, Université Paris Cité, Paris, France; 3 Structural Microbiology Unit, CNRS UMR 3528, Institut Pasteur, Université Paris Cité, Paris, France; 4 Bacterial Cell Cycle Mechanisms Unit, CNRS UMR 3528, Institut Pasteur, Université Paris Cité, Paris, France; 5 Photonic Bio-Imaging, Centre de Ressources et Recherches Technologiques (UTechS-PBI, C2RT), Institut Pasteur, Université Paris Cité, Paris, France; 6 Chemical Biology, Helmholtz Centre for Infection Research, Braunschweig, Germany; 7 Chemogenomic and Biological Screening Core Facility, CNRS UMR 3523, C2RT, Institut Pasteur, Université Paris Cité, Paris, France; 8 Machine Biophotonics Lab, Rudolf Virchow Center, University of Würzburg, Würzburg, Germany; 9 Center for Artificial Intelligence and Data Science, University of Würzburg, Würzburg, Germany

**Keywords:** Machine learning, Image processing, High-throughput screening, Phenotypic screening, Antibiotics

## Abstract

The antimicrobial resistance crisis urgently calls for antibiotics with novel modes of action (MoAs). While growth inhibition assays can identify antibiotic molecules, they miss promising compounds below inhibitory concentrations and cannot reveal their MoA. Imaging-based profiling of drug-treated bacteria can inform on MoA, but current approaches generally require fluorescent labelling and/or inhibitory concentrations and it remains unclear whether compounds with novel MoAs can be robustly detected. Here, we demonstrate a deep learning approach to recognise antibiotic MoAs from unlabelled images. We train a convolutional neural network to predict treatment conditions from brightfield images of *Escherichia coli* exposed to antibiotics covering eight MoAs. Our approach can detect drug exposure at subinhibitory concentrations and allows near-perfect MoA recognition, even when trained on only eight images per treatment condition. Previously unseen compounds are assigned to their correct MoA with good accuracy if the MoA is represented in the training data, and our method can robustly detect MoA novelty in five out of six considered MoAs, enabling microscopy-based identification of new antibiotic classes. We also achieve near-perfect MoA recognition in *Klebsiella pneumoniae*, suggesting applicability to other species. Our approach complements growth inhibition assays and is poised to improve the search for innovative antibiotic compounds.

## Abstract (web summary)

Authors present a deep learning model that recognises antibiotic modes of action (MoA) directly from brightfield images. Their proposed method complements traditional growth inhibition assays in the search for novel antibiotics.

## Introduction

Antimicrobial resistance (AMR) caused 1.27 million deaths in 2019^1^, with death tolls estimated to rise to as much as 10 million deaths a year by 2050^2^. During the last 40 years, very few antibiotics with novel modes of action (MoAs) have been discovered. New resistance mechanisms of various pathogenic bacteria have thus remained unchecked, thereby rendering the existing arsenal of antibiotics increasingly ineffective^3^.

Phenotypic drug discovery (PDD) is an attractive approach to discover antibiotics with new MoAs, since no prior target hypothesis is required. A typical read-out in PDD screens is optical density (OD) of compound-treated bacterial cultures, which serves as a proxy for bacterial cell density. While this read-out allows to identify compounds that inhibit bacterial growth, it precludes the identification of antibacterial compounds at sub-inhibitory levels that could be optimised by medicinal chemistry or be effective in combination therapies. Moreover, these single read-out screens cannot provide information on how an antibacterial compound inhibited bacterial growth, i.e. the MoA^4^.

To overcome these limitations, recent antibacterial screening campaigns^5^ have drawn inspiration from eukaryotic PDD approaches using microscopy images as read-outs^6^. Nonejuie et al.^7^ and Zoffmann et al.^8^ acquired fluorescence microscopy images of bacteria and extracted hand-crafted quantitative descriptors (features) from segmented bacterial cells to perform bacterial cytological profiling (BCP). Importantly, compounds with the same MoA induced similar phenotypic changes, thus enabling the computational clustering of compounds by MoA directly from microscopy images. However, a limitation of early BCP is its reliance on cell segmentation, an error-prone step especially at high cell densities, and the potentially insufficient or unspecific information contained in hand-crafted features^4^. Deep learning (DL) approaches, in contrast, hold the potential to extract highly specific phenotypic features from images in an end-to-end manner without segmentation^4^. Prior work on microscopy images of eukaryotic cells labelled with multiple fluorescent dyes (Cell Painting^9^) has shown that features learned by a convolutional neural network (CNN) can outperform handcrafted features in discriminating between different treatment conditions and associating similar treatment conditions, respectively^10^. Recently, a CNN trained on over 5000 images has been shown to successfully classify the MoA of antibiotics at 1–5 times the minimum inhibitory concentration (MIC) from multi-channel images of fluorescently stained *Mycobacterium tuberculosis* bacteria^11^.

Despite these encouraging efforts, several limitations and questions remain to be addressed before DL analysis of high-throughput microscopy images can be leveraged efficiently in future screens to discover antibiotic compounds with novel MoAs. First, it is unclear if antibiotic compounds can be identified from images at concentrations well below MICs, which would greatly improve the sensitivity of large-scale screens that typically test thousands of compounds at only one or two fixed concentrations. Second, it remains to be determined to what extent unseen compounds can be assigned to their correct MoA and, critically, whether compounds with novel MoAs can be detected, especially at sub-MIC concentrations. Third, fluorescent labelling is expensive, significantly adds complexity to experimental protocols, can introduce batch-to-batch variability, and may interfere with the molecular mechanism of the tested compounds. Whether fluorescent labelling can be dispensed with in favour of brightfield (BF) images in phenotypic screens of bacteria, as recently shown in eukaryotic cells^12^, is unclear, since bacterial cells are roughly an order of magnitude smaller, potentially limiting the range of visible morphological phenotypes. Finally, the performance of DL typically comes at a steep cost in training data, as e.g. ~200 fields of view (FoV) per compound were used to train a CNN in ref. ^11^, imposing limits to scaling up this approach to thousands of compounds and calling for more efficient approaches.

Here, focusing on the model bacterium *Escherichia coli*, we develop and validate a DL approach to extract bacterial phenotypic features end-to-end directly from BF images, thus dispensing with the need for fluorescent staining. We show that from these BF images alone, our model can reveal alterations induced by antibiotic compounds at concentrations well below the MIC and accurately recognise the MoA of compounds when trained on as few as eight images per well. Moreover, our approach can recognise the MoA of unseen compounds by comparing them to reference antibiotics of the same MoA, and determine if a compound has a novel MoA, again from BF images at sub-MIC concentrations only. We also show the successful application of our method to *Klebsiella pneumoniae*. Our results lay the foundations for a time- and cost-effective pipeline to identify chemical compounds with potentially novel antibiotic MoAs.

## Results

### Collecting a high-throughput image dataset of bacterial phenotypes

**Fig. 1:**
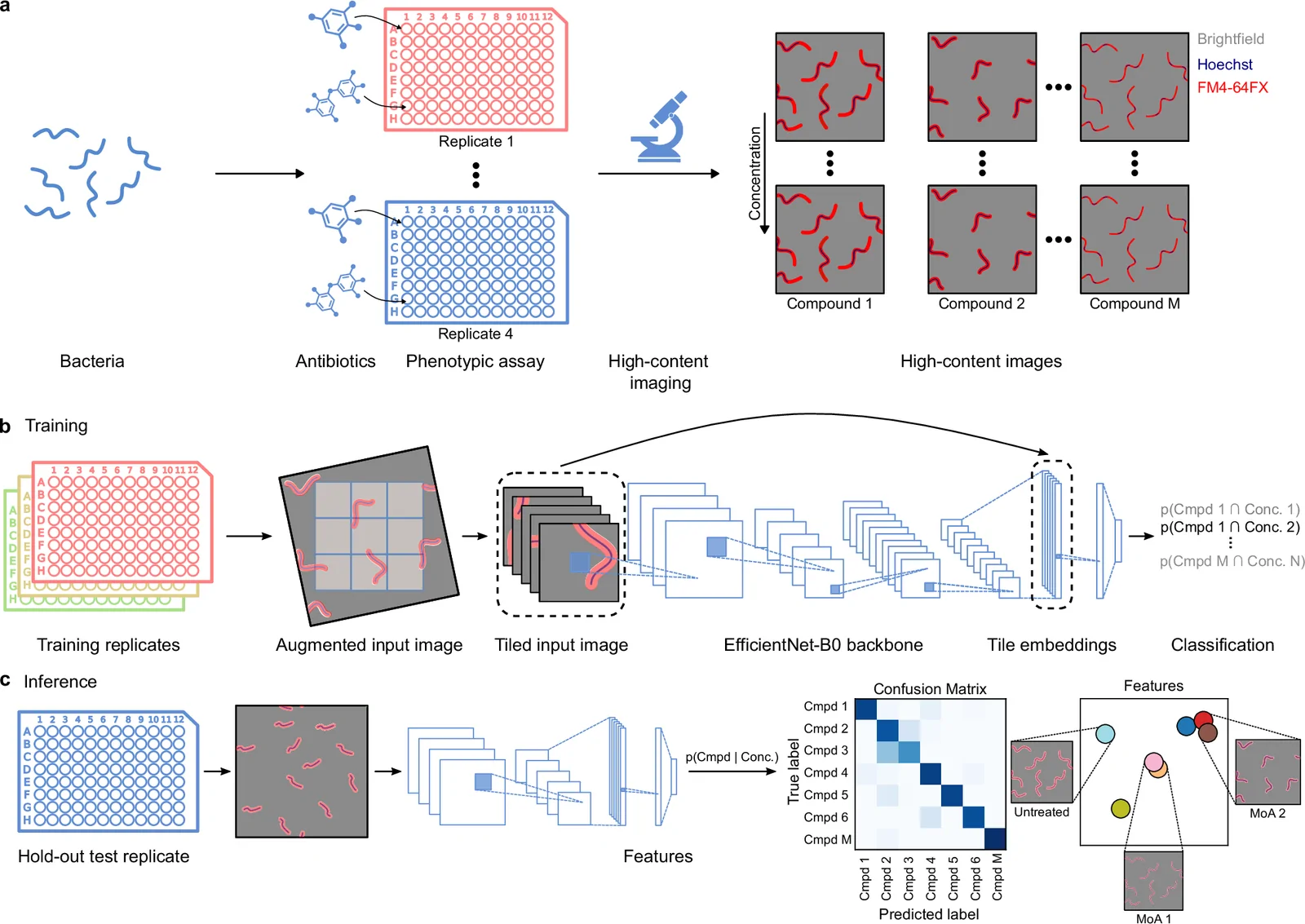
Data acquisition, model training and inference. **a** Bacteria were grown to the exponential phase in culture, antibiotics were distributed onto four 96-well plates at four different concentrations with an acoustic liquid handler, and bacteria were dispatched onto the well plates. Bacteria were then grown with antibiotics for 2 h 15 min and fixed. Plates were imaged on a high-content microscope with a 63× water-immersion objective in non-confocal mode with three channels (BF, FM4-64FX and Hoechst). **b** The model was trained on high-throughput images from three out of four plates to predict the combination of antibiotic compound and concentration with a cross-entropy loss. Images were first passed through a data augmentation pipeline and then tiled. Each tile was then passed through the same EfficientNet-B0 backbone, whereupon embedding vectors from each image tile were aggregated to obtain a global embedding vector. **c** Inference/testing was performed on a hold-out test plate with compounds at a given concentration. Confusion matrices compare classifications (predictions) to ground-truth classes. Embedding vectors are visualised in 2D with UMAP dimensionality reduction. Cmpd., Compound; Conc., Concentration; MoA, mode of action.

**Fig. 2:**
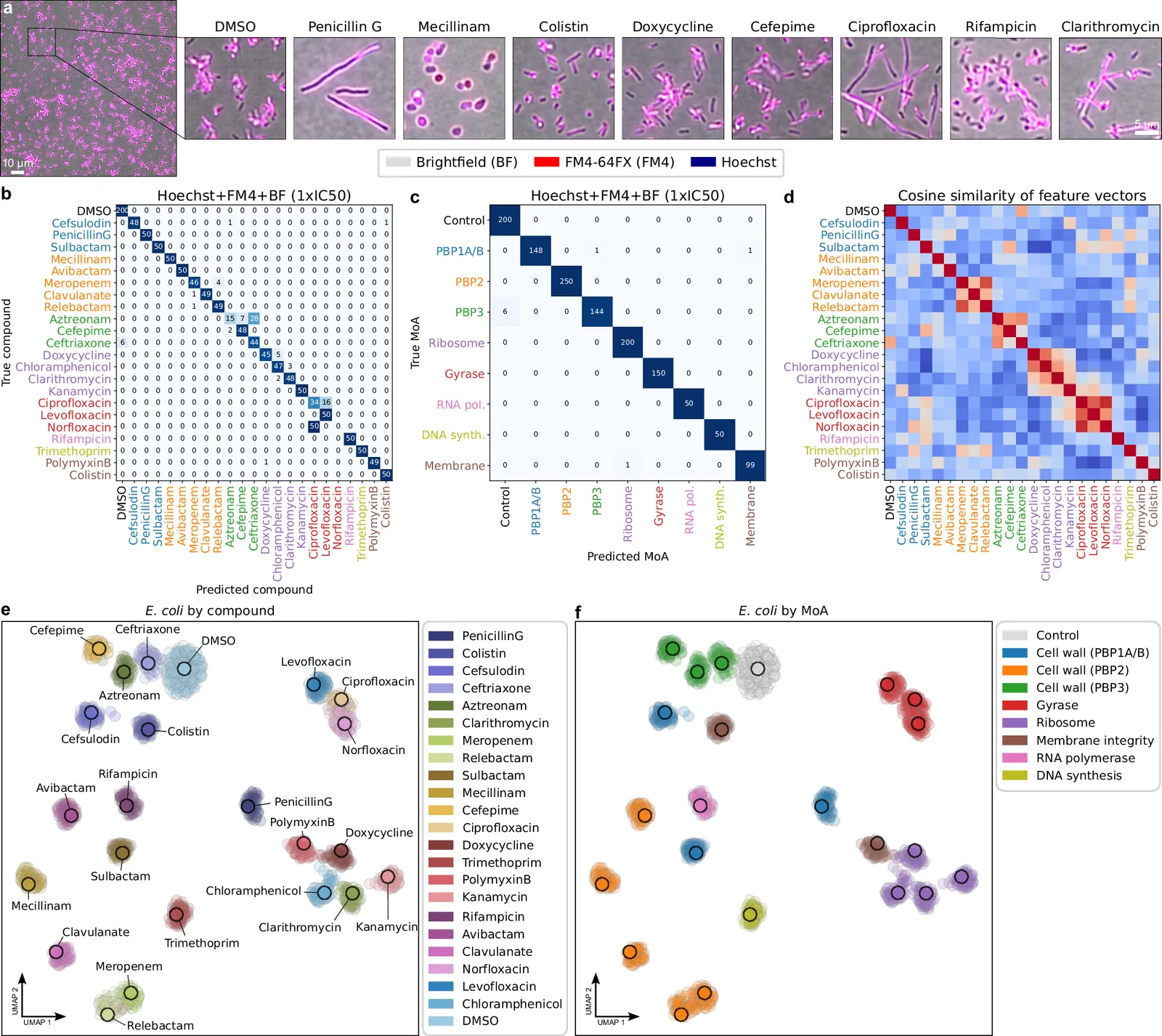
Deep learning recognises compounds and their MoA from *E. coli* images. **a** Representative images of antibiotic-treated *E. coli* (or negative controls, DMSO) from the test plate, with brightfield (BF) shown in grey, the FM4-64FX channel in red and the Hoechst channel in blue. Images were acquired in four biological replicates with 50 FoVs per class (see Supplementary Figs. 2–7 for more examples of BF images). Image contrast was adjusted to improve visualisation. All cropped images from DMSO to clarithromycin are at the same scale. **b** Confusion matrix of compound predictions per FoV from high-throughput images of bacteria treated at IC_50_ using a model trained on all imaging channels and all concentrations. Colours encode the MoA as indicated in (**c**). The confusion matrix corresponds to one hold-out test plate. Supplementary Fig. 9 shows confusion matrices for all four hold-out test plates. **c** Confusion matrix per FoV for the same test plate obtained by pooling compounds within the same MoA (Table 1), with colours matching those in (**b**). **d** Cosine similarities between median feature vectors, with red indicating high similarity and blue indicating low similarity. High similarity tends to be found between antibiotics belonging to the same MoA (e.g. ciprofloxacin, norfloxacin and levofloxacin target the DNA gyrase). Colours encode MoA as in (**b**, **c**). **e** UMAP of image embeddings where smaller dots correspond to individual FoVs and larger dots with black borders to median embeddings over all FoVs in a well. Dots are coloured by antibiotic compounds. **f** Same UMAP with dots coloured by MoA. Antibiotics belonging to the same MoA tend to co-localise in latent space (e.g. PBP3 inhibitors in green and DNA gyrase inhibitors in red). MoA, mode of action. IC50, half maximal inhibitory concentration. Source data are provided as a Source Data file.

We collected a high-throughput imaging dataset of *E. coli* bacteria exposed to 22 widely used reference drugs (Figs. 1a and 2a). We labelled each of these drugs using only one of eight target classes (MoAs) based on available information from the literature (Table 1). Our reference drugs included seven inhibitors of cell wall synthesis targeting penicillin binding proteins (PBPs), which we labelled based on their primary target as inhibitors of PBP1A/B, PBP2 or PBP3; four β-lactamase inhibitors shown to have moderate to weak activity towards these PBPs; four antibiotics targeting ribosomes; three targeting DNA gyrase; two targeting membrane integrity; one targeting RNA polymerase and one targeting DNA synthesis (Supplementary Tables 1 and 2). Relebactam is the only drug for which no targets had been reported in *E. coli*. As it belongs to the family of diazabicyclooctanes (DBOs) that includes the PBP2 inhibitor avibactam, we putatively labelled relebactam as targeting PBP2. For each of these drugs, we determined the half maximal inhibitory concentration (IC_50_) ('Methods'), which ranged from 4 ng/mL to 64 μg/mL (Table 1). We distributed these antibiotics onto four 96-well plates with randomised layouts (Supplementary Fig. 1) using an acoustic liquid handler ('Methods') to expose bacteria to one of four subinhibitory concentrations (0.125 × IC_50_, 0.25 × IC_50_, 0.5 × IC_50_ and IC_50_). Then, we grew bacteria to the exponential phase and seeded them onto the four 96-well plates containing the previously distributed antibiotics ('Methods'). In order to test robustness to biological variability, we performed these experiments as four biological replicates, wherein *E. coli* were cultured independently and grown with randomised well positions in four 96-well plates (Supplementary Fig. 1). After a 2 h 15 min incubation followed by bacterial membrane staining with FM4-64FX for 15 min, we fixed bacteria with a combination of paraformaldehyde and glutaraldehyde, whereupon we stained DNA with Hoechst 33342. Next, we acquired high-throughput images ('Methods') in three channels (BF, FM4-64FX and Hoechst) using an automated high-content screening system in non-confocal mode with a 63× water-immersion objective (Figs. 1a and 2a).

**Table 1:** Antibiotics used for *E. coli*

MoA	Antibiotic	IC_50_ (μg/mL)	MoA reference
Cell wall (PBP1A/B)	Cefsulodin	23.77	Refs. ^24,42^
Cell wall (PBP1A/B)	Penicillin G	25.65	Refs. ^24,43^
Cell wall (PBP1A/B)	Sulbactam	15.62	Ref. ^25^
Cell wall (PBP2)	Avibactam	19.20	Ref. ^43^
Cell wall (PBP2)	Mecillinam	0.3009	Refs. ^24,42,44^
Cell wall (PBP2)	Meropenem	0.1085	Refs. ^43,45^
Cell wall (PBP2)	Clavulanate	17.24	Ref. ^25^
Cell wall (PBP2)	Relebactam	64	N/A for *E. coli*
Cell wall (PBP3)	Aztreonam	0.01568	Ref. ^43^
Cell wall (PBP3)	Cefepime	0.007517	Refs. ^43,46^
Cell wall (PBP3)	Ceftriaxone	0.004	Ref. ^43^
Ribosome	Chloramphenicol	3.079	Ref. ^47^
Ribosome	Clarithromycin	22.24	Ref. ^48^
Ribosome	Doxycycline	0.3703	Ref. ^49^
Ribosome	Kanamycin	2.529	Ref. ^50^
DNA gyrase	Ciprofloxacin	0.005723	Ref. ^51^
DNA gyrase	Levofloxacin	0.01089	Ref. ^52^
DNA gyrase	Norfloxacin	0.02538	Ref. ^53^
RNA polymerase	Rifampicin	3.358	Ref. ^54^
DNA synthesis	Trimethoprim	0.1335	Ref. ^55^
Membrane integrity	Colistin	0.6465	Ref. ^56^
Membrane integrity	Polymyxin B	1.166	Ref. ^57^

The 22 antibiotics used to treat *E. coli*, along with their MoA according to the literature and their potency (IC_50_). Relebactam has no reported target in *E. coli.*

### Deep learning model and training strategy

We then designed a DL model (Supplementary File 1) to map images of antibiotic-treated bacteria to chemical perturbations ('Methods'). We based our model on the CNN architecture EfficientNet-B0^13^, which was shown to achieve state-of-the-art performance on established image classification benchmarks with much fewer parameters than more widely used architectures like ResNet-50^14^ (e.g. 5.3 million parameters vs 26 million parameters and 76.3% vs 76.0% accuracy on ImageNet classification^13,15^), thereby keeping memory requirements and computation time moderate.

As input data, we used images of entire FoVs, of 2160 × 2160 pixels (corresponding to an area of 139.32 × 139.32 μm). Images were passed through a data augmentation pipeline and divided into nine tiles, which were processed in parallel using the same convolutional filters. Tile-wise embedding vectors were aggregated before the final classification layer (Fig. 1b and 'Methods'), thus diminishing the influence of tiles without bacteria on model training. More details on image pre-processing, model architecture, training hyperparameters and data augmentation are provided in 'Methods' and Supplementary Tables 3 and 4.

We trained our model to perform image classification, where each distinct antibiotic drug treatment, i.e. each combination of antibiotic and concentration (e.g. ‘aztreonam’ and ‘0.25 × IC_50_’), was considered as a separate class, resulting in a total of 89 classes (4 concentrations × 22 compounds + 1 negative control treated only with the solvent DMSO; 'Methods'). In order to reduce our model’s sensitivity to possible technical confounders (e.g. plate-specific image features and well position-dependent effects independent of the drug-induced phenotypes) and increase its robustness to biological variability, we used three plates corresponding to three biological replicates to train a single model. Critically, to avoid biases and spurious correlations between training and testing data that could lead to an overestimation of classification performance, we evaluated each model’s classification performance on the biological replicate corresponding to the fourth hold-out test plate (Fig. 1c).

### Deep learning accurately recognises the antibiotic mode of action from images

We first asked if and to what extent our model can predict the combination of both drug and concentration from images alone, considering all the available imaging channels (BF, Hoechst, FM4-64FX). This is a very difficult task, since (i) our treatments include multiple compounds with the same targets, expected to induce very similar phenotypes (Table 1) and (ii) changes in drug concentration are not necessarily expected to induce different phenotypes (e.g. if concentrations are either much below or above the IC_50_). Indeed, although some drug treatments led to easily recognisable phenotypes, such as cell filamentation or shortening, distinguishing between all treatment conditions by visual inspection only remained extremely challenging (Supplementary Figs. 2–7). We assessed classification accuracy (i.e. the percentage of correct classifications) on four rotations of the training and testing plates, with confusion matrices shown in Supplementary Fig. 8. If the model was unable to learn any features specific to the combination of drug and concentration, the expected random classification accuracy would be 1.12% (1/89). Remarkably, the actual classification accuracy ranged from 31.3 to 47.4% at the FoV level, i.e. 28–42 times better than random. Visual inspection of classification errors indicated that the majority of errors were confusions between drugs sharing the same MoA and that misclassifications were more frequent at lower concentrations, where predictions often defaulted to a small number of drugs or the negative control (Supplementary Fig. 8). These results indicate that the model extracted some information specific to drug-concentration combinations from images.

As the effect of antibiotics is likely to be more pronounced at higher concentrations, we next assessed our model (trained on all drug-concentration combinations) only on test data at the highest drug concentrations, i.e. the IC_50_ and assessed accuracy based only on the predicted antibiotic (irrespective of the predicted concentration; 'Methods'). On these data, our model achieved a very high per-image accuracy of 76.2– 90.2%, compared to 4.3% (1/23) for random classification (Supplementary Fig. 9). Per-well accuracies (obtained using the majority of predictions from all images in the same well) were slightly higher, namely 78.3–91.3%, indicating that at the IC_50_, the vast majority of the 22 reference drugs used here led to highly specific phenotypes, which our model was able to automatically learn from the training data (Fig. 2b).

Nevertheless, antibiotic classification was not perfect, as the model still tended to confuse certain compounds. Inspection of individual hold-out test replicates revealed that the model tended to systematically confuse a subset of drugs (Fig. 2b and Supplementary Fig. 9). For example, aztreonam was confused with cefepime, cefsulodin or ceftriaxone for three out of four, and relebactam was confused with meropenem in two out of four pairs of model and hold-out test data, and norfloxacin was systematically mistaken for ciprofloxacin in one hold-out test plate. We noted that the confused compounds tended to have similar MoAs (Table 1). Moreover, when computing cosine similarities between median embeddings for all pairs of antibiotics, we could observe clustering by MoA (Fig. 2d), as also illustrated by UMAPs (Fig. 2e, f), suggesting that our model extracted similar feature vectors from images of compounds targeting the same MoA, despite only having been trained on drug labels and not explicitly on MoA labels.

This observation encouraged us to assess our model’s ability to recognise the MoA, as opposed to the identity of the drugs themselves. For this purpose, we first assigned each of the reference drugs to one out of six MoAs or target classes, namely: cell wall, ribosome, DNA gyrase, RNA polymerase, DNA synthesis and membrane integrity (Table 1). Because half of the antibiotics were cell wall inhibitors (11 out of 22), we further subdivided this class by their main target proteins, namely PBP1A/B, PBP2 and PBP3 (Table 1), thereby leading to eight target classes with one to five antibiotics per class. We then assessed the model’s performance at recognising the MoA of each drug by pooling drugs according to their known MoA. We note that this did not involve any retraining on MoAs, as we still used the model trained on combinations of antibiotics and concentrations. Remarkably, our model achieved an accuracy of 84.1–99.3% in classifying drug MoAs at the image-level and of 100% for two out of four replicates at the well-level (on the remaining plates, the classification accuracies were 87.0 and 95.7%), indicating almost perfect recognition of MoA from images (Fig. 2c).

Thus, our data shows that images of bacteria treated with reference antibiotics at 1 × IC_50_ levels contain highly specific information about drug MoA, allowing accurate MoA recognition from images alone. Moreover, our results indicate that established CNN architectures are well-suited to extract this information in an end-to-end fashion when trained on drug labels alone, without the need for handcrafted features, cell segmentation or prior knowledge of drug MoAs.

### Brightfield images alone allow accurate recognition of drug MoA

**Fig. 3:**
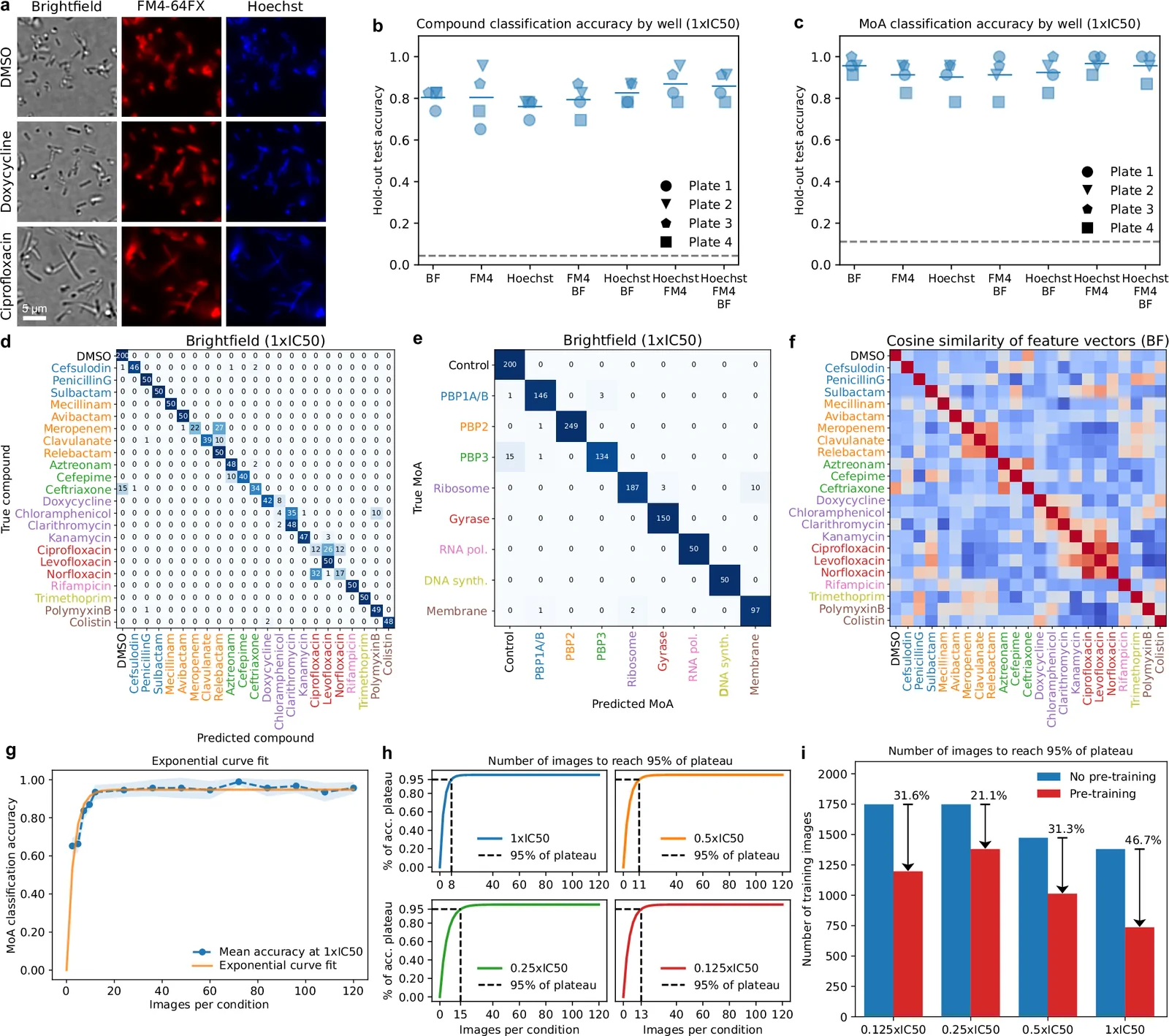
Classification performance for different numbers of imaging channels and training images. **a** Example images of *E. coli* split by imaging channel. Images were acquired in four biological replicates with 50 FoVs per treatment condition. Image contrast was adjusted to improve visualisation. All images are at the same scale. **b** Per-well compound classification accuracies for different combinations of imaging channels. Models were trained in a cross-validation experiment on three plates (biological replicate) and tested on a hold-out test plate containing the same imaging channels as the training images. Test images contain bacteria treated at the same relative concentration (IC_50_). The solid horizontal line indicates the mean, and the dashed line indicates random classification. A Friedman test (*n* = 4 plates, *k* = 7 channel combinations) shows no significant difference between the combinations of imaging channels (*p* = 0.088). **c** Per-well MoA classification accuracies. The solid horizontal line indicates the mean, and the dashed line indicates random classification. A Friedman test (*n* = 4 plates, *k* = 7 channel combinations) shows no significant differences (*p* = 0.053). **d** Confusion matrix by compound per FoV as in Fig. 2b but using only BF images for training and testing. **e** Confusion matrix by MoA per FoV, as in Fig. 2c, but for BF images. **f** Cosine similarities as in Fig. 2d, but based on the BF images. **g**, **h** MoA classification accuracy as a function of the number of training images per condition. The number of images required to reach 95% of the accuracy plateau was estimated by fitting a two-parameter exponential function to averaged MoA classification curves, with an example shown in (**g**) for predictions obtained from images of bacteria treated at IC_50_. The solid line is the average, and the shaded area shows the standard deviation across the four replicates. **h** The number of images per condition required to reach 95% of the accuracy plateau was computed for each concentration on held-out test data, showing that between eight and 15 images are required. **i** Bars show the number of training images required to reach 95% of the accuracy plateau with (red) or without (blue) pretraining on ImageNet, evaluated for different concentrations by cross-validation on four hold-out test plates. The number of required training images (across all conditions) ranged from 736 (1 × IC_50_) to 1380 (0.25 × IC_50_) with pretraining. For **d–i**, all models were trained on BF images only. BF, brightfield; MoA, mode of action; acc., accuracy. Source data are provided as a Source Data file.

Fluorescent labelling of various bacterial cell compartments requires the selection of dyes with compatible excitation and emission spectra, as well as the species-specific optimisation of staining protocols. Additionally, fluorescent staining increases cost, acquisition time and data volumes and technical variations in staining may compromise reproducibility. We therefore asked if all three imaging channels, namely BF, FM4-64FX and Hoechst (Fig. 3a), were necessary to accurately distinguish between antibiotic drugs or MoAs. To quantify this, we trained several CNN models as described previously, but with all possible combinations of the three imaging channels as input and assessed classification accuracy on the hold-out test data (at IC_50_) using the same channels. In some cases, the well-level compound classification accuracy varied significantly between the four hold-out plates, e.g. accuracies ranged from 65.2 to 95.7% for the models trained only on the FM4-64FX channel (Fig. 3b, Supplementary Figs. 10 and 11). We speculate that these differences could be due to variations in staining efficiency between plates. The median well-level compound classification accuracy over the four replicates was highest for the models trained and tested only on the two fluorescent channels (Hoechst and FM4-64FX) and all three channels together (Hoechst, FM4-64FX and BF). To our surprise, however, ignoring one or even two of the channels in training and testing resulted in only small reductions of median accuracy when compared to the differences between plates (Fig. 3b, Supplementary Figs. 10 and 11) and differences in well-level compound classification accuracies between different channel combinations were not significant (Friedman test: *p* = 0.088), suggesting that the three channels contained largely redundant information. When considering the BF channel alone (Fig. 3d), the drug classification accuracy was 80.4 ± 4.3% (mean ± std.dev.), only 5.4% below the three-channel accuracy, and was more consistent across plates (accuracies ranged from 73.9 to 82.6% vs 78.3 to 91.3%). Strikingly, pooling the predictions from the BF-only model by MoA led to almost perfect well-level MoA classification (95.7 ± 3.6%; Fig. 3c, e) and differences were again not significant (*p* = 0.053) when compared to other channel combinations. Accordingly, feature vectors obtained from the BF-only model also clustered by MoA (Fig. 3f).

To shed light on the image features used by our model, we computed saliency maps using Gradient-weighted Class Activation Mapping (Grad-CAM)^16^, which highlights the parts of the image most important for the prediction of each class. Grad-CAM highlighted different features of bacterial cells, rather than the background (Supplementary Fig. 12 and 'Methods'), consistent with the CNN having learned biologically relevant image features as opposed to technical confounders. In composite images of cells treated with antibiotics of distinct MoAs, the class-dependent activations are consistently stronger for the image halves corresponding to the antibiotic class, as expected if the CNN has learned MoA-specific features (Supplementary Fig. 13).

These results show that accurate MoA recognition is possible from BF images alone, thereby removing the need for fluorescent labelling. This could drastically simplify experimental protocols and significantly reduce costs in large microscopy-based phenotypic screens.

### CNN learns MoA-specific features from only eight images per condition

A major drawback of using DL algorithms compared to classical analysis methods based on hand-crafted features is the need for large amounts of training data. Assessing the minimum number of images required to train a DL model without losing classification performance can help to optimise and potentially reduce the burden of data collection and increase the rate of assayed compounds. We therefore estimated the minimum number of images required to train our CNN without adversely affecting MoA classification and also assessed the impact of pretraining our model on a large public dataset of natural images (ImageNet^15^). For this purpose, we trained our CNN using an EfficientNet-B0 convolutional backbone pretrained on ImageNet (Fig. 1b) with varying numbers of bacterial images per condition ranging from three to 120 and computed mean classification accuracies across four replicates. We observed that classification accuracy stopped increasing with the number of training images and fitted a two-parameter exponential function to determine the accuracy plateau and the number of images required to reach it (within 5%), at four different concentrations (Fig. 3g, h and 'Methods'). We found that between eight (IC_50_) and 15 images (0.25 × IC_50_) per condition (736 and 1380 images in total) were sufficient to achieve near-maximum accuracy. This number ranged from seven to nine (at 1 × IC_50_) when training on the 30% images of highest or lowest cellular density, respectively, roughly consistent with an average 1.33-fold difference in mean cell density (Supplementary Fig. 15). Repeating the analysis after retraining the models with randomly initialised weights (Fig. 3i), we found that pretraining on ImageNet data reduced the number of required images by between 21.1% (for 0.25 × IC_50_) and 46.7% (for 1 × IC_50_).

These results show that near-perfect MoA classification performance can be achieved with only eight images per condition for *E. coli* bacteria exposed to compounds at the IC_50_ level and highlight the benefit of using pretrained models to reduce the required training data.

### Detecting drug phenotypes at subinhibitory concentrations

**Fig. 4:**
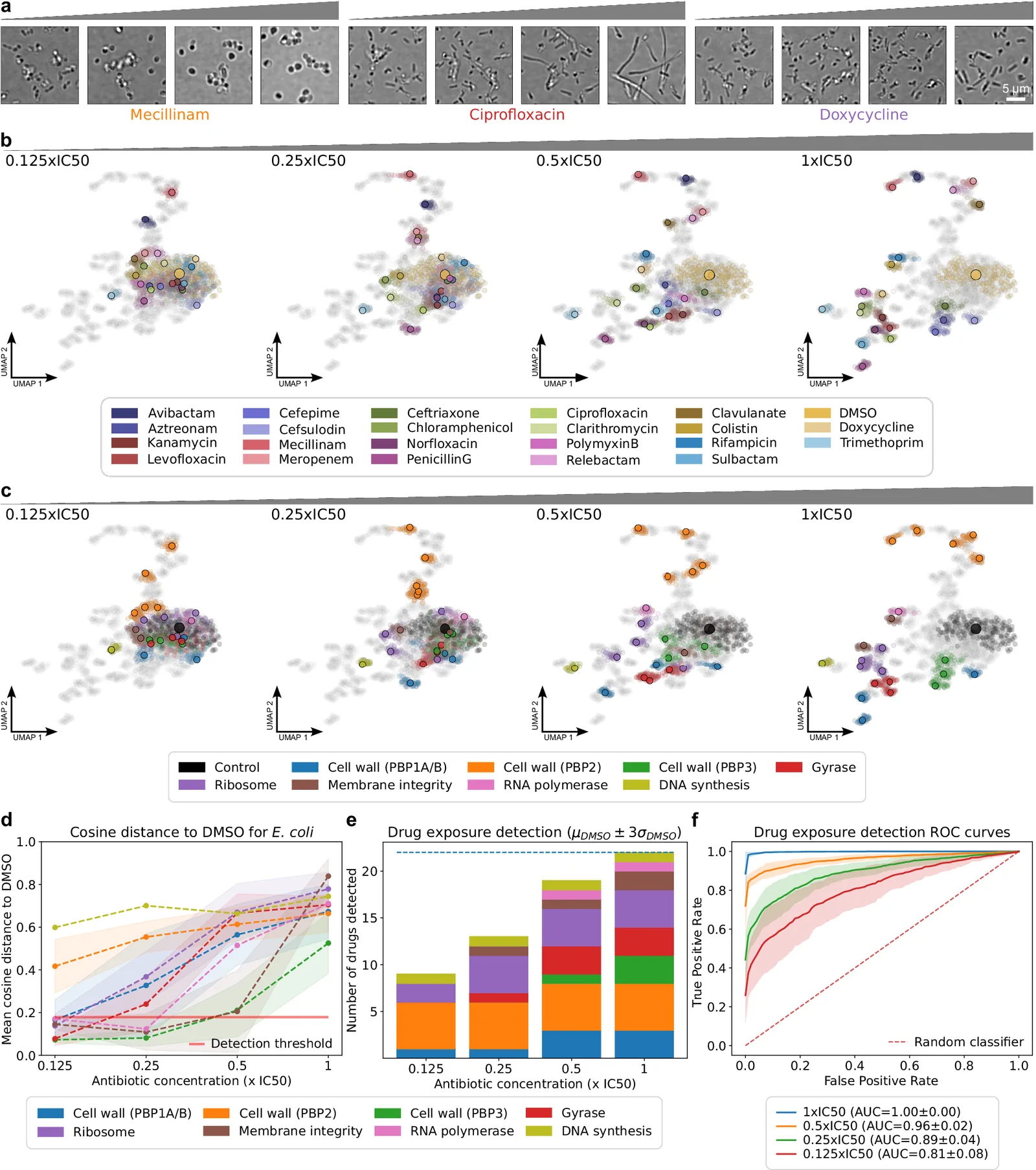
Detecting drug exposure at subinhibitory concentrations. **a** Example images of *E. coli* treated at increasing concentrations (from 0.125 × IC_50_ on the left to 1 × IC_50_ on the right) show dose-dependent phenotypic changes. The antibiotic is indicated with colour encoding the MoA (see legend in **c**). Images were acquired in four biological replicates with 50 FoVs per treatment condition. Image contrast was adjusted to improve visualisation. All images are at the same scale. **b** Feature vectors of bacteria treated at four different concentrations were jointly projected with UMAP, and dots corresponding to a given concentration were highlighted in each plot. Small dots correspond to individual high-throughput images, and larger dots correspond to median feature vectors. Dots are coloured by antibiotics. **c** UMAP plots coloured by MoA show that feature vectors tend to move further away from the negative control (DMSO) as concentration increases. **d** The cosine distance between median feature vectors and DMSO was computed for each antibiotic class at different concentrations. Average distances for each MoA are shown as dashed lines, and shaded areas indicate the standard deviation. A detection threshold (red line) was computed from the intra-class cosine distances of DMSO feature vectors (mean plus three standard deviations). **e** Drug exposure detection was determined by evaluating cosine distances between median feature vectors and DMSO with respect to the detection threshold. The number of detected drugs at each relative concentration is shown, and bars are coloured by their MoA. The dashed line indicates the total number of antibiotics. **f** Receiver operating characteristic (ROC) curves for FoV-level drug exposure detection at different concentrations cross-validated on the four hold-out test replicates with the corresponding area-under-the-curve (AUC) values (mean ± std. dev.). Solid lines correspond to mean ROC curves, and shaded areas indicate the standard deviation. Source data are provided as a Source Data file.

The above results show that DL can reliably extract MoA-specific features from images of bacteria exposed to drugs at 1 × IC_50_, which for the reference drugs considered here ranged from 4 ng/mL to 64 μg/mL (Table 1). Visual inspection of drug-treated bacteria shows that the cellular phenotypes tend to be less pronounced at lower concentrations, as expected (Fig. 4a). When screening large libraries of compounds, systematic titration to determine the IC_50_ for each compound is usually not practical. Instead, chemical screens typically rely on a single concentration, which for most active compounds is generally different from the inhibitory concentration. We therefore asked if our DL method could detect or even classify drug-induced phenotypes at subinhibitory concentrations, and in particular for concentrations at or below the IC_50_. Since at subinhibitory concentrations, new chemical compounds may induce only subtle effects in phenotypic screening campaigns before optimisation by medicinal chemistry, the ability to detect such weak phenotypes could increase the sensitivity and hit rate for antibiotic drug discovery.

We applied our models trained on reference antibiotics at multiple concentrations (as detailed above), on hold-out test data at sub-IC_50_ levels and observed that, unsurprisingly, our ability to correctly classify bacterial images by compound increased with concentration (Supplementary Fig. 16). We then analysed the embeddings computed by our BF-only model on test data at increasing concentrations, from 0.125 × IC_50_ to 1 × IC_50_. For most antibiotics, embeddings were similar to negative controls at the lowest tested concentration (0.125 × IC_50_) and tended to move away from the control and from one another as concentration increased (Fig. 4b, c). The exceptions were the PBP2 inhibitors avibactam and mecillinam, as well as the DNA synthesis inhibitor trimethoprim, for which embeddings already departed markedly from the controls at the lowest concentration, suggesting that these compounds induce distinctive phenotypic changes at concentrations well below inhibitory values. To quantify this observation, we computed the cosine distances between the median embeddings of drug-treated cell images and the negative control (DMSO) images, at each concentration (Fig. 4d). We also defined a threshold distance as the mean plus three standard deviations of the cosine distance between individual DMSO images. Based on this threshold, we found that even at 0.125 × IC_50_, compounds from four groups of antibiotics (inhibitors of DNA synthesis, ribosome, PBP1A/B and PBP2) exhibited sufficiently different phenotypes to be detected (Fig. 4d, e). At 0.25 × IC_50_, all but two groups of antibiotics (RNA polymerase and PBP3 inhibitors) were distinguished from the negative control.

To assess drug exposure detection at different subinhibitory concentrations, we obtained receiver operating characteristic (ROC) curves, which describe the trade-off between the true positive rate (ratio of FoVs with detected drug exposure to actual drug-treated FoVs) and the false positive rate (ratio of FoVs with detected drug exposures to untreated FoVs). These ROC curves were cross-validated on four hold-out plates, and performance was quantified using the area-under-the-curve (AUC), which is 1.0 for perfect detection and 0.5 for random detection. We observed that, in line with Fig. 4e, drug exposure detection improved with increasing concentrations and that even at the lowest concentration (0.125 × IC_50_), drug exposure was robustly detected with an AUC of 0.81 ± 0.08, while at IC_50_, detection was perfect (AUC = 1.00) (Fig. 4f).

Overall, our data show that image embeddings learned by our CNN can be used to robustly detect subtle phenotypic changes at subinhibitory concentrations from BF images alone, thereby enabling highly sensitive detection of potentially active compounds in phenotypic screens.

### Recognising known modes of action of unseen drugs

We showed above that our model can recognise the identity of a compound or the MoA of several antibiotics after training on images obtained in independent experiments with the same antibiotics. However, in the context of a screening campaign, the goal is to determine the MoA of a novel, i.e. previously unseen drug. Therefore, we next asked if our model could correctly recognise the MoA of an unseen compound, i.e. a compound absent from the training data. To address this question, we performed a leave-one-compound-out (LOCO) analysis for compounds sharing a MoA with at least two other compounds.

**Fig. 5:**
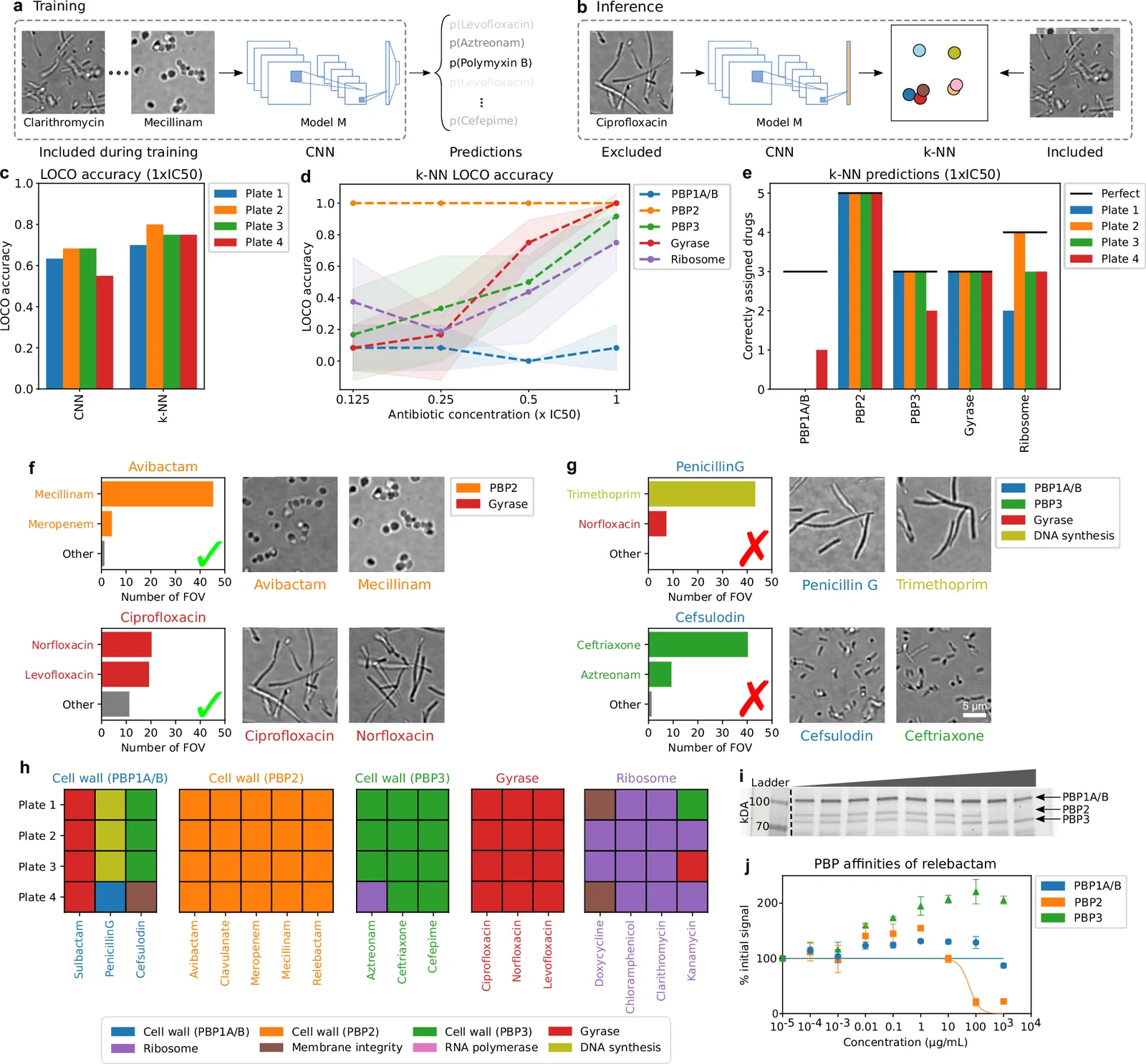
Assigning the MoA of unseen compounds. **a** All images corresponding to one antibiotic (in this example, ciproflocaxin) were systematically left out from the training data, and models were trained in a cross-validated setting on three out of four plates (biological replicates). **b** The MoA of images belonging to the left-out antibiotic class was then determined via k-nearest neighbour (k-NN) classification using the image embeddings of previously seen antibiotics on a hold-out test replicate. **c** Bars show the leave-one-compound-out (LOCO) MoA prediction accuracy at IC_50_ for each hold-out test plate, using either the convolutional neural network (CNN) predictions (left: 63.8 ± 6.3%) or the k-NN classifier (right: 75.0 ± 4.1%). **d** The LOCO MoA prediction accuracy was computed for images of bacteria exposed to different antibiotic concentrations with the k-NN classifier. The dashed lines show the average LOCO accuracy for each MoA, and shaded areas indicate the standard deviation. **e** The number of correctly assigned antibiotics using k-NN classification is shown by MoA for all four hold-out test plates. The black horizontal lines indicate a perfect assignment. **f** Examples of correctly assigned antibiotics showing the assignment of individual fields of views (FOV) to previously seen antibiotics with a k-NN classifier (*k* = 150). Image crops of bacteria exposed to the left-out antibiotic and of bacteria treated with the most frequently associated antibiotic are shown. **g** Examples of incorrectly assigned antibiotics according to literature annotations (Table 1) with image crops showing bacteria exposed to the left-out antibiotic and bacteria treated with the most commonly associated antibiotic. **h** LOCO predictions are shown for each hold-out test plate and antibiotic, with colours indicating the assigned MoA. In **f**–**h**, the colour of each antibiotic encodes its MoA as in Fig. 2c. **i**, **j** The fluorescent penicillin analogue Bocillin FL was used in a competition assay against relebactam at increasing concentrations. **i** Representative gel showing bands of PBP1A/B, PBP2 and PBP3 (the experiment was repeated three times). *E. coli* was treated across a gradient of relebactam concentrations before labelling PBPs with Bocillin-FL. The PBP1A/B, PBP2 and PBP3 bands observed between the 70 and 100 kDa markers were then quantified and plotted. **j** Normalised fluorescence intensity (symbols and error bars show mean and standard deviation of three biological replicates) as a function of relebactam concentration and fitted curves. All images shown are at the same scale. Source data are provided as a Source Data file.

We retrained our CNN (using BF images only) after systematically excluding all images for one compound from the training data (Fig. 5a). Then, using a hold-out test plate, we calculated embeddings from images of bacteria exposed to all reference (i.e. training set) compounds as well as to the left-out compound at 1 × IC_50_. For each FoV, we identified the closest reference compound using a k-NN classifier on embedding vectors (with *k* = 150 and using cosine distance) (Fig. 5b and 'Methods'). To each left-out compound, we then assigned the MoA of the reference compound that was the closest for most FoVs. We found that at a concentration of IC_50_, the assignment accuracy of the k-NN classifier across plates ranged from 70 to 80% (Fig. 5c). Moreover, we observed that the k-NN classification on embeddings performed better on average than directly using the MoA predictions of our model (75.0 ± 4.1% vs 63.8 ± 6.3%, Fig. 5c). We then evaluated MoA assignment accuracy for a range of concentrations and found that accuracy tended to improve with higher concentration (Fig. 5d). Interestingly, drugs targeting PBP2 were perfectly classified across the entire range of concentrations, indicating that these compounds induce highly distinctive phenotypes even at low concentrations.

At the highest concentration (1 × IC_50_), we observed good to excellent MoA prediction accuracy for compounds inhibiting PBP2 (100%), PBP3 (91.7%), DNA gyrase (100%) and ribosome (75%), while compounds targeting PBP1A/B were almost never correctly assigned (1 out of 12 cases; Fig. 5e; see below and Discussion). Overall, the predicted MoA agreed with the ground-truth, i.e. with our literature-based MoA annotations (Table 1), in 56 out of 72 cases (Fig. 5h).

To better understand apparent misclassifications, we investigated MoA assignments in more detail by considering predictions for individual FoVs of bacteria treated with the left-out compounds (Fig. 5f). For instance, in the case of avibactam, out of the 50 FoVs, 45 were assigned to mecillinam and four to meropenem, consistent with the visually similar coccoid morphologies induced by the three compounds (Fig. 5f, h and Supplementary Fig. 3). Since both compounds target PBP2, avibactam was classified as a PBP2 inhibitor, in agreement with its literature-based MoA annotation (Table 1). Similarly, 20 FoVs of bacteria exposed to ciprofloxacin were assigned to norfloxacin and 19 FoVs to levofloxacin, consistent with the visually similar elongated phenotypes induced by all three compounds (Fig. 5f, h and Supplementary Fig. 5). By contrast, the three nominal PBP1A/B inhibitors sulbactam, cefsulodin and penicillin G led to inconsistent MoA predictions (Fig. 5g, h). Penicillin G was associated with the DNA synthesis inhibitor trimethoprim, while cefsulodin was associated with the PBP3 inhibitor ceftriaxone (Fig. 5g). Indeed, visual inspection shows that the morphologies of bacteria exposed to penicillin G resemble those induced by the DNA synthesis inhibitor trimethoprim (long filaments) more so than those induced by cefsulodin, while cefsulodin induced morphologies (shorter filaments) more similar to those induced by the PBP3 inhibitor ceftriaxone than those of penicillin G (Fig. 5g, Supplementary Figs. 2, 4 and 7).

To further investigate these discrepancies in PBP predictions, we performed Bocillin FL competition assays ('Methods') and determined the specificity against different PBPs of a subset of PBP inhibitors studied here, namely clavulanate, cefepime, penicillin G, cefsulodin and sulbactam (Supplementary Fig. 17). Our data confirm that clavulanate, classified by our LOCO analysis as a PBP2 inhibitor (Fig. 5h), is indeed highly specific to PBP2, in agreement with our literature-based annotations (PBP1A/B: 256.9 μg/mL, PBP2: 0.615 μg/mL and PBP3: >1000 μg/mL). Similarly, these data show that cefepime primarily inhibited PBP3, in agreement with our model predictions and annotation (PBP1A/B: 1.724 μg/mL, PBP2: 0.067 μg/mL and PBP3: 0.001 μg/mL). However, the affinity data for two of our three nominal PBP1A/B inhibitors were not consistent with the literature. Indeed, whereas our assay confirmed that cefsulodin primarily targets PBP1A/B (PBP1A/B: 0.748 μg/mL, PBP2: >1000 μg/mL and PBP3: 10.4 μg/mL), in agreement with the literature, neither penicillin G (PBP1A/B: 7.597 μg/mL, PBP2: 64.64 μg/mL and PBP3: 1.977 μg/mL) nor sulbactam (PBP1A/B: 37.4 μg/mL, PBP2: 200.3 μg/mL and PBP3: 102.2 μg/mL) had a specific target, but instead acted as pan-PBP inhibitors. Although these data do not explain the model predictions for cefsulodin, the lack of specificity of penicillin G and sulbactam is a likely cause of their inconsistent assignments by our model (see Discussion).

As pointed out above, we initially labelled relebactam as a PBP2 inhibitor — despite a lack of evidence of its target in *E. coli* — based only on the drug’s structural similarity with avibactam. Our model indeed classified relebactam as a PBP2 inhibitor from the images alone (Fig. 5h). To verify this prediction experimentally, we measured affinities of relebactam to multiple PBPs using the same competition assay (Fig. 5i, j) ('Methods'). This assay confirmed that relebactam indeed selectively inhibits PBP2 as predicted by our model.

Thus, our CNN can learn to extract embeddings from BF images alone that enable accurate MoA prediction of an unseen drug if the MoA is represented by reference compounds with consistent target specificity.

### Detecting drugs with unseen modes of action

**Fig. 6:**
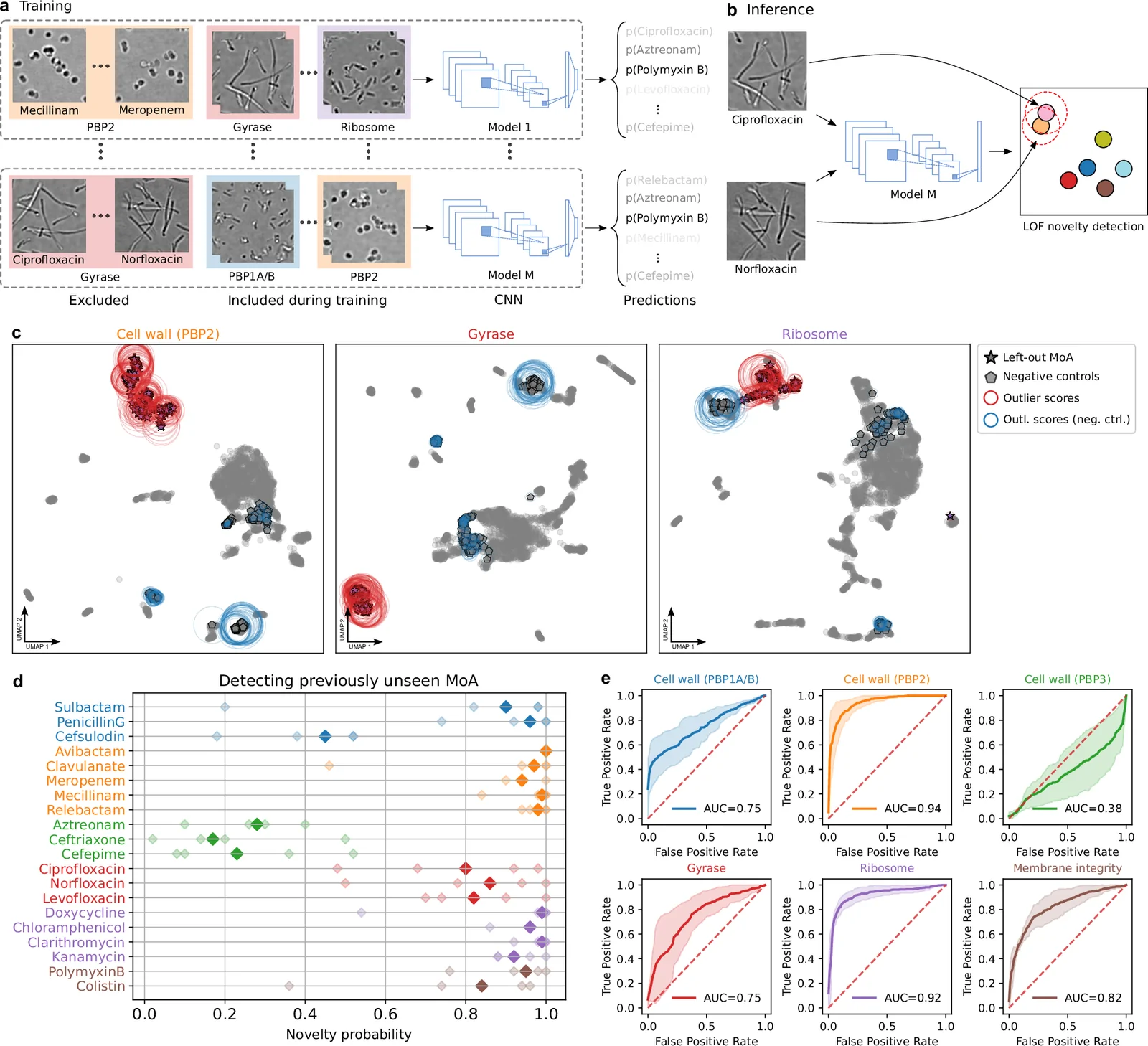
Detecting antibiotics with novel MoAs. **a** Models were trained on images of drug-treated *E. coli* after removing all images corresponding to antibiotics from one MoA (e.g. PBP2 inhibitors for model 1 and DNA gyrase inhibitors for model M), and models were trained in a cross-validated setting on three out of four plates. **b** Embeddings from images of bacteria treated with a compound from the left-out MoA were then obtained and outlier scores assigned with a local outlier factor (LOF) algorithm. **c** UMAPs of embeddings for models trained on compounds from all MoAs except PBP2 (left), DNA gyrase (centre) and ribosome (right). Unsupervised outlier detection was conducted using LOF on embeddings of bacteria exposed to all concentrations (grey dots). Embeddings of compounds from the held-out MoA are shown as stars, negative controls as pentagons and their outlier scores as red and blue circles, respectively. **d** For each compound, probability scores of MoA novelty were computed by thresholding outlier scores and computing the fraction of detected embeddings. Pale symbols show the novelty detection probability for each plate, and the median is shown in bright colours. **e** Cross-validated receiver operating characteristic (ROC) curves for FoV-level MoA novelty detection were obtained for each held-out MoA on four hold-out test plates. Solid lines show averages, and shaded areas the standard deviation across the four replicates. Mean area-under-the-curve (AUC) values are shown. In **d**, **e**, the colour of each antibiotic encodes its MoA as in Fig. 2c. Source data are provided as a Source Data file.

While the above results indicate that known MoAs can be assigned to novel compounds, addressing AMR requires the ability to identify compounds with novel MoAs. To address this, we assessed if our approach is capable of detecting compounds with a MoA that — unlike in our previous analysis — was not seen by our model during training. We therefore designed a leave-one-MoA-out (LOMO) experiment where we singled out one MoA and trained our CNN on BF images of drug-treated bacteria after systematically leaving out all compounds from this MoA (Fig. 6a). To serve as negative controls for novel MoA detection, we also randomly removed one compound for each MoA with at least three compounds (Table 1) from the training data. We then computed outlier scores on image embeddings with a local outlier factor (LOF) algorithm^17^ (Fig. 6b and 'Methods').

After model training, we obtained embeddings from images of bacteria on a hold-out test plate treated with all compounds except those of the left-out MoA and the left-out negative control compounds at all four concentrations (grey dots in Fig. 6c). Next, we defined an image as showing a novel MoA if the outlier score of its embedding exceeded a threshold (and as a known MoA otherwise). We then computed novelty probabilities for each left-out compound (at 1 × IC_50_) by considering the fraction of FoVs that were detected as exhibiting a novel MoA (Fig. 6d). The compounds of the left-out MoA and the negative control compounds allowed us to quantify true and false positives, respectively. We determined ROC curves by cross-validation across four hold-out test plates and computed mean AUC values for each MoA (Fig. 6e).

All compounds targeting PBP2, DNA gyrase, ribosome and membrane integrity exhibited median novelty probabilities above 0.8 and high or very high AUC scores (PBP2: 0.94, DNA gyrase: 0.75, ribosome: 0.92 and membrane integrity: 0.82), indicating that compounds with these MoAs induce phenotypes that can be well differentiated from the MoAs seen during training. In the case of PBP1A/B (AUC = 0.75), penicillin G and sulbactam exhibited high novelty probabilities, but cefsulodin did not (Fig. 6d). For the DNA gyrase inhibitors, median novelty scores across compounds were consistently high (AUC = 0.75), but individual scores by plate exhibited a higher degree of spreading compared to ribosome or PBP2 inhibitors. Conversely, PBP3 inhibitors consistently showed low novelty probabilities (AUC = 0.38), suggesting that these compounds induce phenotypes that do not differ from other MoAs sufficiently to be determined as novel MoAs de novo. Note that the PBP1A/B inhibitor cefsulodin was predominantly classified as a PBP3 inhibitor in our LOCO analysis (Fig. 5h), indicating that cefsulodin induces PBP3-like morphologies. The consistently low novelty scores observed for PBP3 inhibitors could thus also explain the low novelty scores of cefsulodin. However, this was dependent on concentration, since at 4 × IC_50_ or higher, PBP3 inhibitors led to a clear change in phenotype, enabling good novelty detection (AUC = 0.86) (Supplementary Fig. 18).

Taken together, our results show that compounds from all studied MoAs induce phenotypes that are robustly detected as novel when excluded from the training data at concentrations of IC_50_, except for PBP3 inhibitors, where concentrations of 4 × IC_50_ were required. Our approach could thus play a key role in identifying compounds with potentially novel MoAs in phenotypic screens.

### MoA recognition from brightfield images of *Klebsiella pneumoniae*

**Fig. 7:**
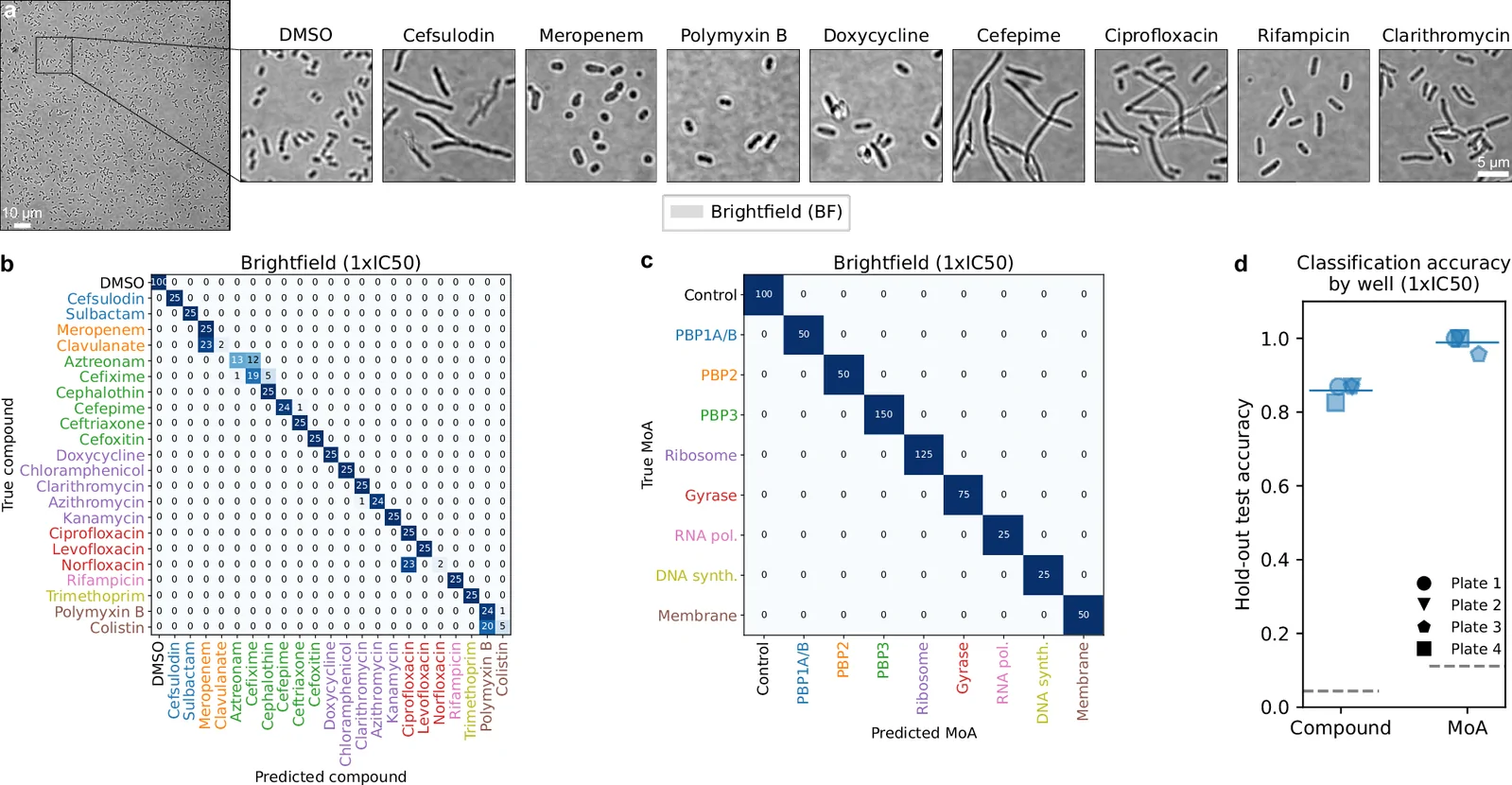
Recognising drug MoA from brightfield images in *K. pneumoniae.* **a** Brightfield (BF) images of *K. pneumoniae* bacteria treated with antibiotics or negative controls (DMSO). Images were acquired in four biological replicates with 25 FoVs per treatment condition (see Supplementary Figs. 19–24 for more examples). Image contrast was adjusted to improve visualisation. **b** Confusion matrix for compound prediction per FoV from BF images of *K. pneumoniae* treated with drugs at 1 × IC_50_ in one hold-out test plate. Confusion matrices for all hold-out test plates are shown in Supplementary Fig. 26. **c** Confusion matrix by mode of action (MoA) per FoV for the same test plate. Confusion matrices for all hold-out test plates are shown in Supplementary Fig. 27. In **b**, **c**, the colour of each antibiotic encodes its MoA as in Fig. 2c. **d** Well-level classification accuracies of compounds and MoAs across four hold-out test plates (with treatments at 1 × IC_50_), using models trained on the remaining three plates (biological replicates). The solid horizontal line indicates the mean. Dashed horizontal lines indicate accuracies of random classification. Source data are provided as a Source Data file.

Finally, we tested the applicability of our approach beyond the model bacterium *E. coli*. For this purpose, we turned to *Klebsiella pneumoniae*, another member of the *Enterobacteriaceae* family. *K. pneumoniae* has a slightly larger genome than *E. coli* (~5.5 Mb vs 4.5 Mb and more than 5000 vs ~4400 coding sequences). These differences contribute to different antibiotic susceptibilities, and *K. pneumoniae* is resistant to four out of the 22 antibiotics used for *E. coli*. We therefore replaced these by four antibiotics to which *K. pneumoniae* is sensitive and exposed bacteria grown from a master frozen stock culture to the 22 antibiotics, at concentrations of 0.25 × IC_50_, 0.5 × IC_50_, 1 × IC_50_ and 2 × IC_50_. We grouped these antibiotics by MoA based on literature annotations as indicated in Table 2. For each drug and each concentration, we acquired 25 BF images of unstained cells on four plates (biological replicates) with randomised layouts using the same automated high-throughput microscopy system as for *E. coli*. Example images are shown in Fig. 7a and Supplementary Figs. 19–24.

**Table 2:** Antibiotics used for *K. pneumoniae*

MoA	Antibiotic	IC_50_ (μg/mL)	MoA reference
Cell wall (PBP1A/B)	Cefsulodin	57.98	PBP1, *E coli* : ref. ^42^
Cell wall (PBP1A/B)	Sulbactam	19.95	PBP1a, *E. coli*; ref. ^25^
Cell wall (PBP2)	Meropenem	0.03311	PBP2, *K. pneumoniae*; ref. ^58^
Cell wall (PBP2)	Clavulanate	20.94	PBP2, *E coli*; ref. ^59^
Cell wall (PBP3)	Aztreonam	0.006467	PBP3, *K. pneumoniae*; ref. ^58^
Cell wall (PBP3)	Cefepime	0.0129	PBP3, *K. pneumoniae*; ref. ^58^
Cell wall (PBP3)	Cefixime	0.01869	PBP2, *Neisseria gonnorhea* (homolog of *E. coli* PBP3); ref. ^60^
Cell wall (PBP3)	Cefoxitin	7.165	PBP1+3, *E. coli*; ref. ^61^
Cell wall (PBP3)	Ceftriaxone	0.009485	PBP3, *E coli*; ref. ^42^
Cell wall (PBP3)	Cephalothin	9.647	PBP3, *Bacteroides fragilis* (homolog of *E. coli* PBP3); ref. ^62^
Ribosome	Chloramphenicol	1.978	Ref. ^47^
Ribosome	Clarithromycin	70.61	Ref. ^48^
Ribosome	Azithromycin	5.735	Ref. ^63^
Ribosome	Doxycycline	2.335	Ref. ^49^
Ribosome	Kanamycin	2.271	Ref. ^50^
DNA gyrase	Ciprofloxacin	0.02229	Ref. ^51^
DNA gyrase	Levofloxacin	0.0691	Ref. ^52^
DNA gyrase	Norfloxacin	0.07824	Ref. ^53^
RNA polymerase	Rifampicin	16.19	Ref. ^54^
DNA synthesis	Trimethoprim	0.6577	Ref. ^55^
Membrane integrity	Colistin	2.01	Ref. ^56^
Membrane integrity	Polymyxin B	1.638	Ref. ^57^

The 22 antibiotics used to treat *K. pneumoniae*, along with their MoA according to the literature (with the species from which they were inferred) and their potency (IC_50_).

We first attempted to use the model trained on *E. coli* images directly to analyse the *K. pneumoniae* images, after application of a sphering transform to align negative control wells ('Methods'). Although this analysis allowed some meaningful clustering of compounds based on feature similarities (e.g. clustering of the ribosome inhibitors azithromycin and clarithromycin or clustering of the PBP2 inhibitors meropenem and clavulanate), it also produced some incorrect or unexpected groupings, e.g. the DNA synthesis inhibitor trimethoprim was clustered with the gyrase inhibitor norfloxacin, while three compounds (cefoxitin, sulbactam and chloramphenicol) were clustered with negative controls (Supplementary Fig. 25).

We therefore retrained our CNN from scratch on *K. pneumoniae* images to predict treatment conditions (i.e. drug + concentration) from three plates and evaluated classification accuracies on the fourth hold-out test plate, following the same approach as for *E. coli*. For evaluation, we focused on bacteria treated at 1 × IC_50_. On the drug classification task (i.e. ignoring concentration), our model achieved per-image accuracies ranging from 79.1 to 86.6%, and per-well accuracies ranging from 82.6 to 86.9% (Fig. 7b, d and Supplementary Fig. 26), a similarly high accuracy as for *E. coli*. When pooling predicted drugs by MoA (Table 2), we achieved per-image accuracies between 93.7 and 100%, depending on the replicate, and per-well accuracies of 100% for three out of the four replicates (and 95.7% for the remaining replicate; Fig. 7c, d and Supplementary Fig. 27). Thus, our data show that near-perfect MoA-recognition from BF images is possible in another Gram-negative bacterium of clinical interest, suggesting broader applicability across bacterial species.

## Discussion

**Fig. 8:**
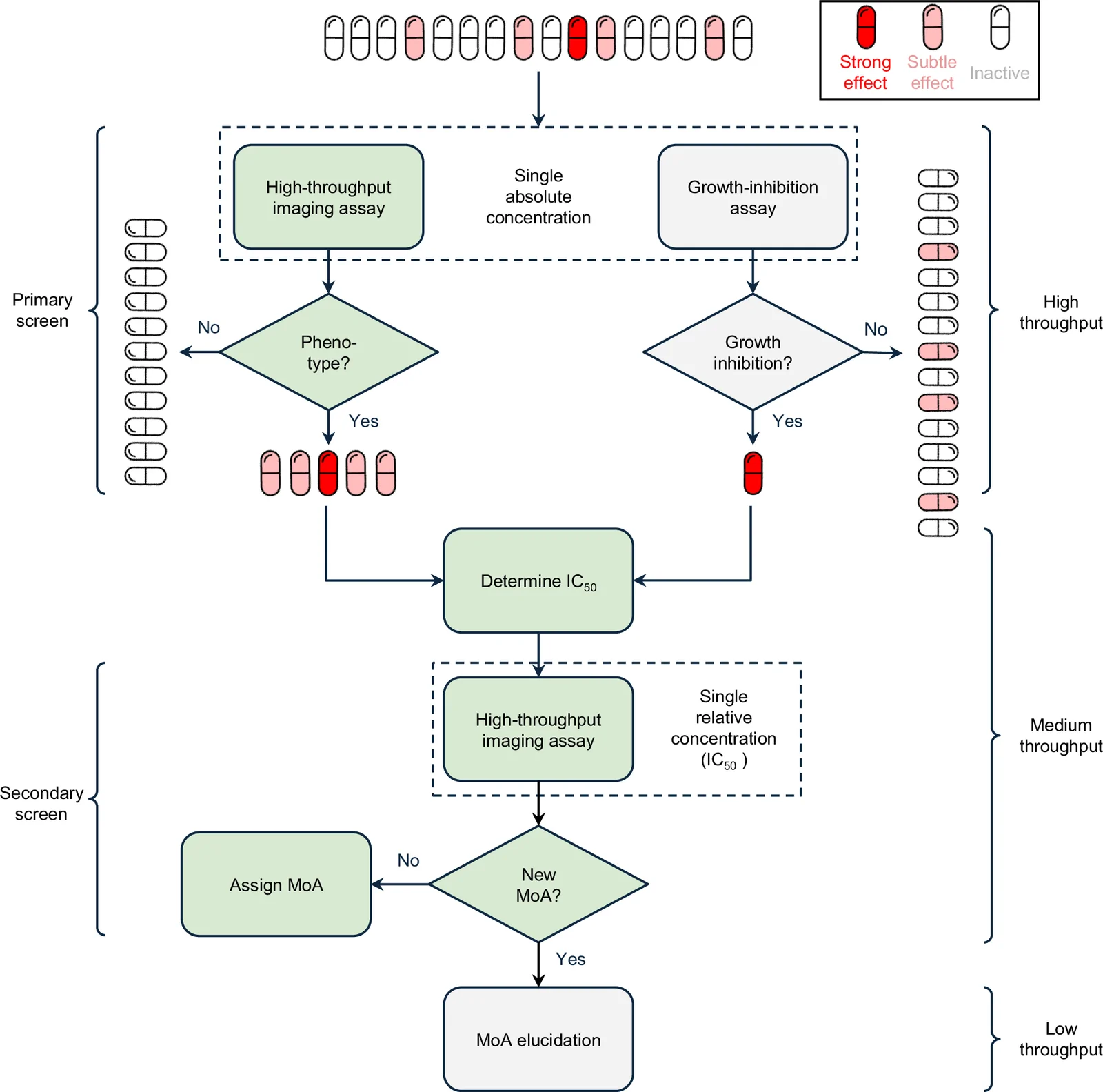
Complementarity of the proposed imaging-based pipeline with growth inhibition assays. In chemical screens, compound libraries typically contain inactive compounds (white) and active compounds with strong or subtle effects (red and pink, respectively). Primary high-throughput screens based on microscopy or growth assays usually examine compounds at a single absolute concentration. Imaging-based screens can detect weak phenotypes induced by compounds at sub-inhibitory concentrations (pink), which are missed by growth inhibition screens (see Fig. 4). For the subset of compounds that elicit a phenotype either based on imaging or on growth inhibition, concentration–response curves are measured to determine IC_50_’s. In a secondary screen, high-throughput imaging of these compounds at IC_50_ allows to predict if compounds have a novel MoA. If yes, follow-up experiments are in order to elucidate the MoA. If not, MoA can be assigned computationally from the images alone. Green boxes highlight the imaging and analysis methods described in this paper.

In this study, we introduced a deep learning (DL) approach based on convolutional neural networks (CNNs) that can extract meaningful phenotypic features from microscopy images of bacteria treated with antibiotic compounds and show that these features can be used for multiple downstream tasks with high relevance for phenotypic drug screening. These tasks include the recognition of mode of action (MoA) or even compound identity directly from images (Figs. 2, 3), the detection of weak phenotypes at subinhibitory drug concentrations (Fig. 4), the assignment of drug MoAs for novel compounds (Fig. 5) and the identification of compounds with novel MoAs (Fig. 6). While we focused on *E. coli*, our analysis of *K. pneumoniae* data also supports excellent recognition of drug MoA in a second bacterial species of high relevance to AMR (Fig. 7). We propose that our imaging-based approach provides a valuable complement to widely used growth inhibition screens, thanks to its ability to identify compounds at sub-inhibitory concentrations and its ability to either recognise compound MoA by comparison to reference antibiotics or detect novel MoAs (Fig. 8).

Our work builds on prior studies in eukaryotic cells and bacteria that established cytological profiling to analyse drug-induced phenotypes and group drugs by MoA, whether using handcrafted features^7,8^, or more recently, DL approaches^4,10,11,18^. However, our study represents a significant extension of these early approaches in several ways.

First, we establish that DL can extract both MoA- and compound-specific features from brightfield (BF) images alone, enabling near-perfect MoA recognition without any fluorescent labelling (Figs. 3c, e and 7c). This came to us as a surprise, since most microscopy-based drug screens employ a mixture of fluorescent dyes to characterise subcellular features, as in the popular Cell Painting assay^9,19,20^ or bacterial cell profiling assays^5,7,8,11^. Our finding that fluorescent labelling of bacterial membranes and DNA is redundant with BF images is consistent with studies showing that DL models can be trained to generate realistic fluorescent labelling or coloration (virtual staining) in silico from label-free images in eukaryotes^21^ and in bacteria, as illustrated by a recent report that Gram-staining can be predicted from darkfield images of *E. coli* and *Listeria innocua*^22^. They are also in line with a recent related study demonstrating MoA recognition in unlabelled *E. coli* using time-resolved holographic microscopy^18^. Our results extend previous work in eukaryotic cells, showing that DL applied to BF images performs on par with classical Cell Profiler features to determine drug MoA^12^. More importantly, our findings enable a drastic simplification of the experimental workflow of phenotypic imaging screens by removing the need for staining and protocol optimisation, as well as avoiding the associated costs and potential confounding factors like variations in fluorescent staining. The removal of staining also facilitates phenotypic screening in live cells, potentially circumventing fixation issues and enabling the incorporation of live cell dynamics in phenotyping.

Second, although DL-based image recognition for natural images typically requires thousands of training images per class to reach state-of-the-art classification accuracies, we show that roughly eight FoVs per condition (i.e. compound and concentration) are sufficient to achieve highly accurate MoA recognition, and that acquiring more images does not yield significant improvements (Fig. 3g–i). The main reason for this surprisingly low requirement is probably the large number of cells contained in each FoV, and the relative simplicity of bacterial BF images compared to natural images like those from the ImageNet dataset. Nevertheless, our observation that pretraining on ImageNet significantly reduces the required number of images suggests that many ImageNet features are also relevant for BF *E. coli* images. Our finding that recognition accuracy saturates with only a handful of FoVs should encourage future phenotypic imaging screens to reduce image acquisition time per well and thereby increase the rate of assayed compounds.

Third, we emphasise that our model was trained solely to predict the combinations of drugs and concentrations and thus requires no explicit prior knowledge of the MoA. Despite this, we show that our model implicitly encodes similarities by MoA in its latent space. This is a welcome feature, as it means that a straightforward supervised learning strategy with well-defined and unambiguous labels (drugs and concentrations) can learn biologically meaningful representations from image data in spite of incorrect MoA annotations. On the contrary, our approach can be used to flag potential MoA annotation errors as showcased in our LOCO analysis for the supposed PBP1A/B inhibitors cefsulodin, sulbactam and penicillin G (see below).

Fourth, we show that DL can robustly detect weak phenotypes induced by drugs at concentrations well below inhibitory levels (Fig. 4). This result implies that large chemical libraries at a fixed concentration can be screened with high sensitivity, i.e. with a high likelihood of picking up active compounds that elicit subtle phenotypes in the images without affecting bacterial growth. In a second step, these hit compounds could then be titrated to determine their IC_50_ and analysed by imaging for MoA assignment at 1 × IC_50_ levels, along with active compounds identified by traditional growth inhibition assays (Figs. 5c–h and 8).

Fifth, we have addressed the critical task of determining if a novel compound also has a novel MoA^18^. We have shown robust novelty detection for five out of six tested MoAs, with excellent performance (specificity and sensitivity) for PBP2 and ribosome inhibitors, and good to very good performance for inhibitors of PBP1A/B, DNA gyrase and membrane integrity. We acknowledge poor performance for PBP3 inhibitors at concentrations up to 1 × IC_50_ but note that performance improves at concentrations of 4 × IC_50_ or higher (Supplementary Fig. 18). Despite imperfections, our method thus opens the door to screening large libraries of compounds using high-throughput microscopy to identify compounds that are likely to act through novel MoAs (Fig. 8).

Finally, we also showed near-perfect MoA recognition in *K. pneumoniae*, using a model trained only on BF images of this bacterium, indicating that our approach extends to other bacterial species (Fig. 7).

We acknowledge several limitations or open questions. While MoA assignment in the LOCO analysis was good for compounds belonging to four MoA groups, it is notable that almost none of the nominal PBP1A/B inhibitors were correctly assigned to this MoA. Instead, each compound was assigned to a different MoA (Fig. 5h). The phenotypic discordances underlying these predictions and the results of our PBP affinity assays call the literature-based grouping of these PBP1A/B inhibitors into question (Table 1 and Supplementary Table 1). Cefsulodin has been reported to selectively target PBP1A/B (PBP1A: 0.47 μg/mL, PBP1B: 3.7 μg/mL, PBP2: >250 μg/mL and PBP3: >250 μg/mL)^23,24^. However, this is not supported by our PBP affinity assay (Supplementary Fig. 17), where we observed inhibition of both PBP1A/B (0.75 μg/mL) and PBP3 (10.4 μg/mL). Sulbactam has been reported to primarily target PBP1A with low affinities for PBP2 and PBP3^25^, but this is also inconsistent with our affinity assays, where we observe broad inhibition of PBP1A/B, PBP2 and PBP3 (Supplementary Fig. 17). By contrast, the high reported affinity of penicillin G for multiple PBPs (PBP1A: 0.5 μg/mL, PBP1B: 3.0 μg/mL, PBP2: 0.8 μg/mL, PBP3: 0.9 μg/mL and PBP4: 1.0 μg/mL)^24^ is consistent with our PBP affinity assay showing a pan-PBP inhibitor activity (Supplementary Fig. 17). Taken together, these differences in PBP affinities between putative PBP1A/B inhibitors plausibly explain the observed inconsistencies in bacterial phenotypes. We note that the discrepancies in PBP affinities between our experiments and prior studies could result from variations in bacterial strains due to their individual history of pre-exposure to β-lactams. Cefsulodin is the only drug in our dataset that primarily targets PBP1A/B (0.75 μg/mL), although with high secondary affinity for PBP3 (10.4 μg/mL). Nevertheless, it is classified as a PBP3 inhibitor by our LOCO analysis (Fig. 5g). In this context, we also note that recent work has shown that PBP1A and PBP1B do not regulate the morphology of *E. coli* bacteria^26^ and instead contribute to cell-wall integrity, whereas PBP3 directly regulates bacterial cell shape. If inhibition of PBP1A/B does not produce a detectable phenotype, it appears likely that morphological changes induced by cefsulodin will be driven by inhibition of PBP3 alone. This would be consistent with our model’s classification of cefsulodin as a PBP3 inhibitor. In the future, predictions could also benefit from characterising the specificity profile of compounds to non-essential low molecular weight PBPs that we ignored here (e.g. PBP4-7), whose inhibition may affect bacterial phenotypes^27^. In any case, the discrepancies between the predicted MoAs of compounds that nominally share the same MoA (such as cefsulodin, sulbactam and penicillin G in Fig. 5h) suggest that MoA annotations should be revisited. Corrections of MoA ground-truth assignments flagged by our image-based approach, e.g. by affinity assays, are then likely to yield improved image-based prediction accuracies.

Additionally, our study included four β-lactamase inhibitors (sulbactam, clavulanate, avibactam and relebactam). Although sulbactam has been reported to preferentially bind PBP1A, we show that it is a pan-PBP inhibitor, while we confirm that clavulanate targets PBP2 as reported (Supplementary Fig. 17). Relebactam, however, has not been studied in *E. coli,* and our LOCO analysis indicates that this drug induces PBP2-like phenotypes similar to avibactam and clavulanate (Fig. 5h). Indeed, our affinity assay confirmed that relebactam selectively targets PBP2 in our *E. coli* strain (Fig. 5i, j). This is consistent with data on other DBOs like avibactam that target PBP2. Interestingly, prior work shows that in *Pseudomonas aeruginosa* avibactam preferentially binds PBP1B and PBP4, while relebactam acts as a pan-PBP inhibitor^26^. However, we note that in the same study, an agnostic target predictor methodology classifies both avibactam and relebactam as PBP2 inhibitors, in agreement with our data.

Intriguingly, while trimethoprim is commonly classified as a DNA synthesis inhibitor and specifically targets folate synthesis, some studies have shown that trimethoprim also directly targets the peptidoglycan pathway^28,29^. Indeed, peptidoglycan synthesis relies on nucleotide-activated sugars, such as UDP-N-acetylglucosamine and UDP-N-acetylmuramic acid. Inhibition of nucleotide synthesis by trimethoprim would thus also affect peptidoglycan synthesis and, more generally, cell wall biogenesis, which relies on many nucleotide-activated sugars, including lipopolysaccharides. The assignment of penicillin G to trimethoprim appears consistent with these prior findings.

Taken together, these observations highlight the limitations of grouping compounds into discrete MoA classes and of using reference compounds that inhibit multiple targets. One promising avenue to overcoming this limitation is to predict drug targets by comparing their induced phenotypes to those of large mutant libraries.

The results in our study are limited to *E. coli* and *K. pneumoniae*, two Gram-negative bacteria with pronounced morphological changes in response to drug exposure. To what extent our BF-only methodology extends to Gram-positive bacteria and/or other Gram-negative species with more subtle morphological phenotypes remains to be determined. However, we point out that a variation of our deep learning-based analysis method proved effective at analysing images of the Gram-positive bacterium *Corynebacterium glutamicum*, a proxy for *Mycobacterium tuberculosis*, despite its significantly smaller size and less pronounced phenotypes, as detailed in ref. ^30^. Follow-up work could investigate the applicability of our approach to a broader range of bacterial species.

At the technical level, it will be interesting to determine the robustness of our method to changes in experimental conditions, such as microscopy settings. We verified that MoA recognition accuracy is robust to changes in the image bit-depth (16-bit vs 8-bit) and to a roughly 2-fold change in image resolution (500 × 500 vs 256 × 256 pixels) (Supplementary Fig. 14). Additionally, we acquired BF images of unstained *E. coli* in a different laboratory with a conventional, manually operated imaging system. Compounds were administered at concentrations ranging from 0.25 to 2 × IC_50_, based on independently determined IC_50_ values, and many other experimental conditions differed (Supplementary Note 1, Supplementary Tables 5 and 6). After retraining our CNN on this new data set, we achieved a mean MoA recognition accuracy per well on hold-out test plates of 69.6% at 1 × IC_50_ and 81.9% at 2 × IC_50_ (Supplementary Fig. 28). Despite the lower accuracies, these data suggest that our approach is relatively robust to changes in experimental conditions and may be applied to characterise compound MoA even without automated microscopy and liquid handling systems. This could considerably extend the reach of our method, including to low-resource settings. Nevertheless, we acknowledge that more analyses beyond these preliminary data will be needed to better define the experimental conditions required for reliable MoA recognition from BF images alone.

Finally, we acknowledge that despite our use of Grad-CAM saliency maps (Supplementary Figs. 12 and 13), our approach provides limited insights into the MoA-specific image features learned by the CNN. Leveraging recent advances in explainable AI to overcome limitations of Grad-CAM or similar methods and extract more detailed information about these features provides another exciting perspective for follow-up studies^31–33^. A related limitation is our treatment of concentration labels in the same manner as compound labels. We speculate that better performance on compound classification might be achievable by reframing the prediction of concentration as a regression task or by enforcing the learning of features robust to changes in concentration.

Overall, our work is a step towards deploying DL-based analysis of microscopy images to bacterial phenotypic screens. To facilitate uptake by the community, we make all our analysis methods and pretrained models available, along with the data they were trained on. As sketched in Fig. 8, we propose that our CNN approach and the downstream analysis methods described here complement traditional growth-inhibition assays in a comprehensive phenotypic screening pipeline aiming to discover novel classes of antibiotics to fight AMR.

## Methods

### Bacterial growth protocol for *E. coli*

*E. coli* K-12 MG1655 was grown in 5 mL of cation-adjusted Mueller–Hinton Broth II (MHBII, Fisher Scientific Cat. 11800482) overnight at 37 °C until the stationary phase. The culture was then diluted to 0.05 OD in fresh MHBII and grown at 37 °C for 1 h 45 min until mid-exponential phase between 0.5 and 0.7 OD before further dilution. IC_50_ values were determined via broth serial dilutions in flat-bottomed 96-well plates (TPP Cat. 92096) with a starting inoculum density of 0.05 OD in a total volume of 100 μL. Micro dilution plates were incubated at 37 °C, 180 rpm for 18 h. The final cell density was measured on a Tecan Spark Multimode Plate Reader and plotted on GraphPad Prism to calculate the IC_50_ values with the ‘[Inhibitor] vs response – Variable slope (four parameters)’ formula.

For microscopy, antibiotics (see Supplementary Table 2 for further information on the source of the antibiotics) were dispensed into 96-well plates in nanolitre volumes using an Echo 550 liquid handler (Beckman Coulter) before adding 100 µL of bacteria from mid-exponential growth diluted to 0.05 OD. The plates were incubated at 37 °C, 180 rpm for 2 h 15 min. Then, 20 μg/mL of FM4-64FX (Invitrogen Cat. F34653) was added and was further incubated at 37 °C, 180 rpm for another 15 min. The supernatant was then removed after centrifugation at 4000 × *g* for 5 min. 100 µL of fixative (2.8% paraformaldehyde, 0.04% glutaraldehyde in PBS) was added, and the plates were incubated at room temperature (RT) at 800 rpm for 30 min. Cells were washed twice with 200 µL of PBS by centrifugation and resuspension. Then, cells were suspended in 100 µL of PBS containing 10 µg/mL of Hoechst 33342 (Invitrogen Cat. H1399) and transferred to PhenoPlates (Revvity Cat. 6055300) to determine the OD at 600 nm. Finally, the entire plate was diluted based on the median OD of the negative control wells (DMSO) to a calculated OD of 0.036 in 100 µL. The fixed and diluted cells were stored at 4 °C until imaging.

### Bacterial growth protocol for *K. pneumoniae*

*K. pneumoniae KP1* was grown in 5 mL of cation-adjusted Mueller–Hinton Broth II (MHBII, Fisher Scientific Cat. 11800482) overnight at 37 °C until the stationary phase. The culture was then diluted to 0.05 OD in fresh MHBII and grown at 37 °C for 1 h 45 min until mid-exponential phase between 0.5 and 0.7 OD before further dilution. IC50s were determined via broth serial dilutions in 96-well plates with a starting inoculum density of 0.05 OD in a total volume of 100 μL. Micro dilution plates were incubated at 37 °C, 700 rpm for 18 h. The final cell density was measured on a Tecan Spark Multimode Plate Reader and plotted on GraphPad Prism to calculate the IC_50_ values.

For microscopy, antibiotics were dispensed into 96-well plates in nanoliter volumes using an Echo liquid handler (Beckman Coulter) before adding 100 µL of bacteria from mid-exponential growth diluted to 0.05 OD. The plates were incubated at 37 °C, 700 rpm in an orbi-shaker (Heidolph Titramax 1000) for 2 h 30 min. The OD was measured at 600 nm, and 10 µL of 10× fixative (20% paraformaldehyde, 1% glutaraldehyde in PBS) was added to the cell cultures, followed by a 15 min incubation at RT. Next, 10 µL of fixed cells were transferred to a new plate containing 90 µL of 0.2 M glycine in PBS; the cell density was adjusted in each well with 0.2 M glycine if necessary. Subsequently, 30 µL of the diluted cells were transferred to PhenoPlates (Revvity Cat. 6055300) prefilled with 200 µL of 0.005% PEI in PBS. The plate was centrifuged at 500 × *g* for 10 min and washed three times with PBS. Finally, the plate was stored overnight at RT prior to imaging. All four biological replicates (plates) were obtained and imaged on the same day.

### Penicillin-binding protein affinity assay

*E. coli* K-12 MG1655 was grown in 5 mL of LB overnight at 37 °C until the stationary phase. The culture was then diluted to 0.05 OD in fresh LB and grown at 37 °C for 1 h 30 min until mid-exponential phase between 0.5 and 0.7 OD. Bacteria were collected by centrifugation at 3220 × *g* for 5 min and washed with 50 mM Tris, pH 7.8. Then, bacteria were suspended in 50 mM Tris, 200 mM EDTA, pH 7.8 to 20 OD. 25 μL of 10-fold serially diluted antibiotics starting at 2 mg/mL were aliquoted into a V-bottomed plate, and 25 μL of 20 OD bacteria was added for a final starting antibiotic concentration of 1 mg/mL. The plate was incubated at 20 °C for 30 min and then centrifuged at 3220 × *g* for 5 min to pellet the cells. The supernatant was removed, and cell pellets were suspended in 50 μL of 50 mM Tris, 200 mM EDTA, pH 7.8, containing 25 μM of Bocillin-FL (Invitrogen Cat. B13233). The plate was incubated at 20 °C for 30 min, 1400 rpm. The cells were collected by centrifugation, suspended in 100 μL of 50 mM Tris pH 7.8, and centrifuged again before removing the supernatant. Fifty microliters of Laemmli sample buffer was added, and the plate was incubated at 70 °C for 10 min at 800 rpm before centrifugation at 3220 × *g* for 10 min to pellet insoluble materials. Twenty-five microliters of each sample were run on 4–15% Tris-Glycine SDS-PAGE (Bio-Rad, Cat. 4568084). The gels were imaged on a Bio-Rad Chemidoc at 488 nm to visualise the Bocillin-FL labelled bands and at stain-free settings to visualise total protein content.

### High-throughput imaging

Prior to imaging, plates stored at 4 °C were left at RT for at least 20 min and then centrifuged at 35 × *g* for 10 min to bring floating bacteria to the well bottom. Images were then acquired using an automated Opera Phenix Plus High-Content Screening System (Revvity) with 63×/NA 1.15 water immersion objective in widefield mode without binning. For *E. coli*, two imaging sequences were set up: one for the simultaneous acquisition of fluorescent channels by two cameras, and one for the BF acquisition via a dedicated camera. Hoechst 33342 and FM4-64FX were imaged with a 405 and 488 nm excitation laser line and a 435–480 nm and 650–680 nm bandpass filter, respectively. Images were acquired with exposure times of 80 ms for BF, 400 ms for FM4-64FX and 250 ms for Hoechst 33342. In total, 50 FoVs were acquired in non-overlapping, randomly chosen positions within the whole area of each well. These random positions in each well were the same within individual plates but varied between plates. For imaging *K. pneumoniae*, we used a similar workflow, except that we acquired only BF images in 25 FoVs arranged on a grid at the centre of each well.

### Image preprocessing

Images for training were obtained by converting high-throughput images from 16-bit to 8-bit and clipping pixel intensities by percentile between 0.1 and 99.9 to remove spurious saturated pixels. Images from three out of four plates were then randomly split into training (80%) and validation (20%) sets. During training, full-resolution images with 2160 × 2160 pixels were passed through a data augmentation pipeline: images were randomly flipped, rotated and subjected to random changes in brightness, contrast, saturation and hue (for images with more than one channel). To account for potential edge effects due to random rotation, full images were further centre-cropped (1528 × 1528 pixels) (Fig. 1b). Next, from this centre-crop, a random crop of size 1500 × 1500 pixels was obtained, and images were subdivided into nine tiles of size 500 × 500 pixels. Tiles were then resized to 256 × 256 pixels and pixel intensities normalised between −1 and 1. The latter normalisation choice was preferred to the standard normalisation (by subtraction of mean and division by standard deviation) to reduce the effect of changes in cell density. See Supplementary Table 3 for a detailed overview of the data preprocessing pipeline.

### Removal of technical variation with sphering

For the analysis of *K. pneumoniae* data using features learned on *E. coli* (Supplementary Fig. 25), we used negative control sphering^34^ to remove unwanted technical variation by assuming that negative controls (DMSO) should lead to consistent feature vectors. Variations that arise between control wells are thus assumed to be technical. The sphering transform seeks to find a new space where negative controls have the same amount of variation in each feature dimension. This is achieved by first calculating a covariance matrix on the feature vectors from the negative controls and obtaining its eigenvalues. The eigenvalues of the covariance matrix are then used to compute a correction matrix that whitens the feature vectors of the negative controls. Here, we used zero component analysis (ZCA) to whiten the data. The correction matrix is then applied to all feature vectors from images of drug-treated bacteria. This reduces large variations that are assumed to be due to technical variations.

### Deep learning model training

The DL model was trained for 250 epochs (unless stated otherwise) using an Adam^35^ optimiser with a learning rate of 0.001. A batch size of 16 images was used. Note that the effective batch size was 144 (16 × 9), since each image was further subdivided into nine tiles. During training, tiles were passed through an EfficientNet-B0 backbone, with weights initialised by pretraining on ImageNet^1^ (except where indicated otherwise). Latent feature vectors corresponding to individual tiles were aggregated by averaging to obtain a global feature vector for the full-resolution input image. The model was trained to predict the treatment condition, i.e. the combination of antibiotic and concentration as a multi-class classification task with a cross-entropy loss using L2 regularisation with *λ* = 0.001 to avoid overfitting. To allow the model to accept images with different numbers of input channels, the first layer of the EfficientNet-B0 architecture was replaced with a convolutional layer (torch.nn.functional.Conv2d) with the appropriate number of input channels. Models were trained with PyTorch 2.3.1^36^ in Python 3.9 on the HPC cluster at Institut Pasteur Paris using NVIDIA A40 or A100 GPUs with training times of approximately 30–40 h (depending on the GPU). See Supplementary File 1 for the full model architecture and Supplementary Table 4 for an overview of the model hyperparameters.

### Computing compound predictions conditioned on the dose

For every FoV, the DL model outputs a logits vector ${{{\mathbf{z}}}} {{{\mathbf{\in }}}}{{\mathbb{R}}}^{K}$, where K corresponds to the total number of compound-dose classes (including controls). Applying the softmax function yields a joint probability $p\left(k\right)={e}^{{z}_{k}}/{\sum}_{k{\prime} }{e}^{{z}_{k{\prime} }}$ over all compound-dose classes where k encodes a unique compound-dose pair. Since the dose is known at inference, we can obtain the conditional probability of compound c at dose d with:

$$P\left(\left.{\mbox{compound}}=c\right|{\mbox{dose}}=d\right)=\frac{p\left({k}_{c,d}\right)}{{\sum}_{{k}^{{\prime} }\in {K}_{d}}p\left({k}^{{\prime} }\right)}$$

where ${k}_{c,d}$ is the class encoding compound c and dose d, and ${K}_{d}$ is the set of classes at dose d. The predicted compound $\hat{c}$ is then obtained by computing $\hat{c}={{\mbox{arg}}\max }_{c} \, P\left(\left.{\mbox{compound}}=c\right|{\mbox{dose}}=d\right)$ and well-level predictions are then obtained by a majority vote over all FoVs in a given well.

### Estimating the minimum number of required images

To estimate the minimum number of required training images, we fit an exponential $f(x)\,={a}(1-\,{e}^{-{bx}})\,$ (where x is the number of images per condition) to the mean MoA accuracies obtained from hold-out test data with models trained on different numbers of images per well ranging from x=3 to x=120. From these exponential fits, we obtained the accuracy plateau a and estimated the number of images per well to achieve 95% of this plateau as $x=-{{\mathrm{ln}}}(0.05)/b$. We used the curve fitting function from the scipy^37^ package in Python 3.9.

### Splitting images by bacterial density

The normalised bacterial density D was estimated by thresholding the FM4-64FX channel with Otsu’s method and computing the fraction of foreground pixels as:

$$D=\frac{{{{\rm{\#pixel}}}}{{{{\rm{s}}}}}_{{{{\rm{foreground}}}}}}{{{{{\rm{\#pixels}}}}}_{{{{\rm{foreground}}}}}+{{{\rm{\#}}}}{{{{\rm{pixels}}}}}_{{{{\rm{background}}}}}}.$$

Images were then split into low, medium and high-density images based on the normalised bacterial density distribution for each condition. For a given condition, low bacterial density was defined as images below the 30th percentile, medium as between the 30th and 70th percentile and high as above the 70th percentile of the normalised bacterial density distribution.

### MoA assignment with k-NN classifier

Embeddings were obtained from a hold-out test plate using a model trained on all except one compound from the other three plates. A k-NN classifier was then applied to predict the MoA of the unseen compound using  embeddings from the hold-out test plate corresponding to the highest concentration (IC_50_) and using MoA labels as targets with the scikit-learn^38^ package in Python 3.9. The number of neighbours was set to *k* = 150 and the cosine distance (1 minus the cosine similarity) was used  and points were weighted by the inverse of their distance, thereby ensuring that closer neighbours had a greater influence on the MoA assignment than neighbours which were further away. This analysis was repeated for each model with a left-out compound across four hold-out test plates.

### MoA novelty detection with the local outlier factor algorithm

Embeddings from images of previously seen conditions were obtained from a hold-out test plate. A LOF classifier was applied to these embeddings using all available concentrations with the scikit-learn package in Python 3.9. The number of neighbours was set to *k* = 9, and cosine distance (one minus the cosine similarity) was used as the distance metric. To determine if a compound was an outlier, i.e. had a novel MoA, the local outlier factor on embeddings from the highest concentration (IC_50_) was computed and a threshold applied as described in ref. ^17^. For each compound, novel MoA detection probabilities were computed as the fraction of detected outliers considering all FoVs. ROC curves were obtained by computing the local outlier factor for embeddings corresponding to previously unseen compounds and to left-out negative control conditions (one per MoA with at least three compounds) and positive controls (i.e. compounds with the left-out MoA). AUC values were computed with the scikit-learn package. This analysis was repeated for each model with a left-out MoA across four hold-out test plates.

### Grad-CAM saliency maps

The Grad-CAM algorithm was implemented as described in ref. ^16^. All Grad-CAM maps were obtained from the third convolutional block of the EfficientNet-B0 model as:

$${L}_{{{{\rm{Grad}}}}-{{{\rm{CAM}}}}}^{c}={{{\rm{ReLU}}}}\left({\sum}_{k}{\alpha }_{k}^{c}{A}^{k}\right)$$

where A are the activations, c is the class for which the Grad-CAM is evaluated, k is the index of the feature map and ${{{\rm{ReLU}}}}$ is the rectified linear unit. Grad-CAM maps were then rescaled to the size of input images with bicubic interpolation.

Grad-CAM activations ${L}^{A}$ w.r.t. class A (e.g. avibactam at 1 × IC_50_) and ${L}^{B}$ w.r.t. class B (e.g. trimethoprim at 1 × IC_50_) were computed on composite images composed of a half image from class A and a half image from class B. Grad-CAM values were then summed over the pixels p with *x* corresponding to the set of pixels belonging either to class *x*, *A* or *B*. Relative activations ${R}_{A}^{B}$ for class A compared to class B were then computed as:

$${R}_{A}^{B}(x)=\frac{{\sum }_{p\in x}{L}_{p}^{A}}{{\sum }_{p\in x}{L}_{p}^{A}+{\sum }_{p\in x}{L}_{p}^{B}}.$$

### Statistical tests

Rank-based, one-way comparisons of multiple groups on a quantitative variable were performed using the ‘friedman.test’ function (‘stats’ package), stratifying by replicate. Differences were considered significant for *p*-values below 0.05. In case of statistical significance, a post-hoc analysis was performed using the ‘pairwise.wilcox.test’ function (‘stats’ package, stratifying by replicate, after adjusting for the multiple comparisons by a Benjamini–Hochberg method). These analyses were performed using R (version 4.6.0).

### Software packages

Data analysis was carried out in Python 3.9. The following Python packages were used with version numbers in brackets: ‘torch’ (1.12.1), ‘torchvision’ (0.13.1), ‘numpy’ (1.23.1), ‘scipy’ (1.9.1), ‘pandas’ (1.5.0), ‘matplotlib’ (3.6.0), ‘Pillow’ (9.2.0), ‘scikit-image’ (0.19.3), ‘imageio’ (2.22.0), ‘tifffile’ (2022.8.12), ‘imagecodecs’ (2024.1.1), ‘scikit-learn’ (1.1.2), ‘umap-learn’ (0.5.3), ‘xmltodict’ (0.13.0), ‘lxml’ (4.9.1), ‘openpyxl’ (3.1.2), ‘tensorboard’ (2.10.0) and ‘huggingface_hub[torch]’ (1.7.2).

### Reporting summary

Further information on research design is available in the Nature Portfolio Reporting Summary linked to this article.

## Supplementary information

Supplementary Information

Reporting Summary

Transparent Peer Review File

Supplementary File 1

## Source data

Source Data File

## Data Availability

The imaging data generated in this study have been deposited on Bioimage Archive under accession code S-BIAD1851^39^. The embeddings and model predictions generated in this study have been deposited on Zenodo^40^. Source data are provided with this paper.

## Footnotes

1 https://download.pytorch.org/models/efficientnet_b0_rwightman-7f5810bc.pth

## Code availability

The source code for the computational analyses described in this paper is available with MIT license on GitHub (https://github.com/krentzd/ai4ab) and Zenodo^41^. Model weights are available on Hugging Face (https://huggingface.co/krentzd/ai4ab).
