# Supplementary material for: Deep learning recognises antibiotic modes of action from brightfield images
Source: Nat Commun. 2026 Sep 8;17:10660. doi: 10.1038/s41467-026-76355-0 (PMC13655182; doi:10.1038/s41467-026-76355-0)
Supplement: Supplementary file 1 [file 41467_2026_76355_MOESM1_ESM.pdf]

Supplementary information

Deep learning recognises antibiotic modes of action from brightfield images

Daniel Krentzel[1,*,✉], Kelvin Kho[2,*], Julienne Petit[3,4,*], Nassim Mahtal[5], Thomas Delerue[2], Max E. Huber[6], Agnès Zettor[7], Jeanne Chiaravalli[7], Spencer L. Shorte[5], Mark Brönstrup[6], Anne Marie Wehenkel[4], Ivo G. Boneca[2,#,✉] and Christophe Zimmer[1,8,9,#,✉]

[1]Imaging and Modeling Unit, Institut Pasteur, Université Paris Cité, Paris, France

[2]Unité de Biologie et Génétique de la Paroi Bactérienne, CNRS UMR6047, INSERM U1306, Institut Pasteur, Université Paris Cité, Paris, France

[3]Structural Microbiology Unit, CNRS UMR 3528, Institut Pasteur, Université Paris Cité, Paris, France

[4]Bacterial Cell Cycle Mechanisms Unit, CNRS UMR 3528, Institut Pasteur, Université Paris Cité, Paris, France

[5]Photonic Bio-Imaging, Centre de Ressources et Recherches Technologiques (UTechS-PBI, C2RT), Institut Pasteur, Université Paris Cité, Paris, France

[6]Chemical Biology, Helmholtz Centre for Infection Research, Braunschweig, Germany

[7]Chemogenomic and Biological Screening Core Facility, CNRS UMR 3523, C2RT, Institut Pasteur, Université Paris Cité, Paris, France

[8]Machine Biophotonics Lab, Rudolf Virchow Center, University of Würzburg, Würzburg, Germany

[9]Center for Artificial Intelligence and Data Science, University of Würzburg, Würzburg, Germany

*These authors contributed equally

#These authors jointly supervised this work

✉Correspondence: daniel.krentzel@pasteur.fr, ivo.gomperts-boneca@pasteur.fr, christophe.zimmer@uni-wuerzburg.de

Contents

Supplementary Figure 1: Pseudo-randomised plate layouts. 3
Supplementary Figure 2: Images of E. coli treated with PBP1A/B inhibitors. 4
Supplementary Figure 3: Images of E. coli treated with PBP2 inhibitors. 5
Supplementary Figure 4: Images of E. coli treated with PBP3 inhibitors. 6
Supplementary Figure 5: Images of E. coli treated with DNA gyrase inhibitors. 7
Supplementary Figure 6: Images of E. coli treated with ribosome inhibitors. 8
Supplementary Figure 7: Images of E. coli treated with membrane integrity, RNA polymerase and DNA synthesis inhibitors. 9
Supplementary Figure 8: Confusion matrices for treatment classification from E. coli images. 10
Supplementary Figure 9: Confusion matrices for drug recognition in E. coli. evaluated at IC_{50}. 11
Supplementary Figure 10: Per-image classification accuracy for different input channel combinations. 12
Supplementary Figure 11: Confusion matrices for different channel combinations at IC_{50}. 13
Supplementary Figure 12: Saliency maps highlight bacterial features. 14
Supplementary Figure 13: Counterfactual saliency maps and quantification of relative activation. 15
Supplementary Figure 14: MoA recognition accuracy for different training settings. 16
Supplementary Figure 15: Influence of cell density on required number of training images. 17
Supplementary Figure 16: Confusion matrices for sub-IC_{50} concentrations. 18
Supplementary Figure 17: Identifying PBP specificities with Bocillin FL competition assays. 19
Supplementary Figure 18: PBP3 inhibitors are detected as novel MoAs at higher concentrations 20
Supplementary Figure 19: Images of K. pneumoniae treated with PBP1A/B inhibitors. 21
Supplementary Figure 20: Images of K. pneumoniae treated with PBP2 inhibitors. 22
Supplementary Figure 21: Images of K. pneumoniae treated with PBP3 inhibitors. 23
Supplementary Figure 22: Images of K. pneumoniae treated with DNA gyrase inhibitors. 24
Supplementary Figure 23: Images of K. pneumoniae treated with ribosome inhibitors. 25
Supplementary Figure 24: Images of K. pneumoniae treated with membrane integrity, RNA polymerase and DNA synthesis inhibitors... 26
Supplementary Figure 25: Clustering images of drug-treated K. pneumoniae using features learned on E. coli. 27
Supplementary Figure 26: Confusion matrices for drug recognition in K. pneumoniae. 28
Supplementary Figure 27: Confusion matrices for MoA recognition in K. pneumoniae. 29
Supplementary Figure 28: Robustness to non-automated imaging of E. coli 30
Supplementary Table 1: PBP specificities from literature. 31
Supplementary Table 2: Antibiotic source 32
Supplementary Table 3: Data preprocessing pipeline. 33
Supplementary Table 4: Hyperparameter settings 34
Supplementary Table 5: Experimental conditions in replication experiments 35
Supplementary Table 6: Comparison of antibiotic IC_{50} values against E. coli determined at Institut Pasteur and HZI. 36
Supplementary Note 1: Experimental procedures for replication experiments 37
Supplementary References 38

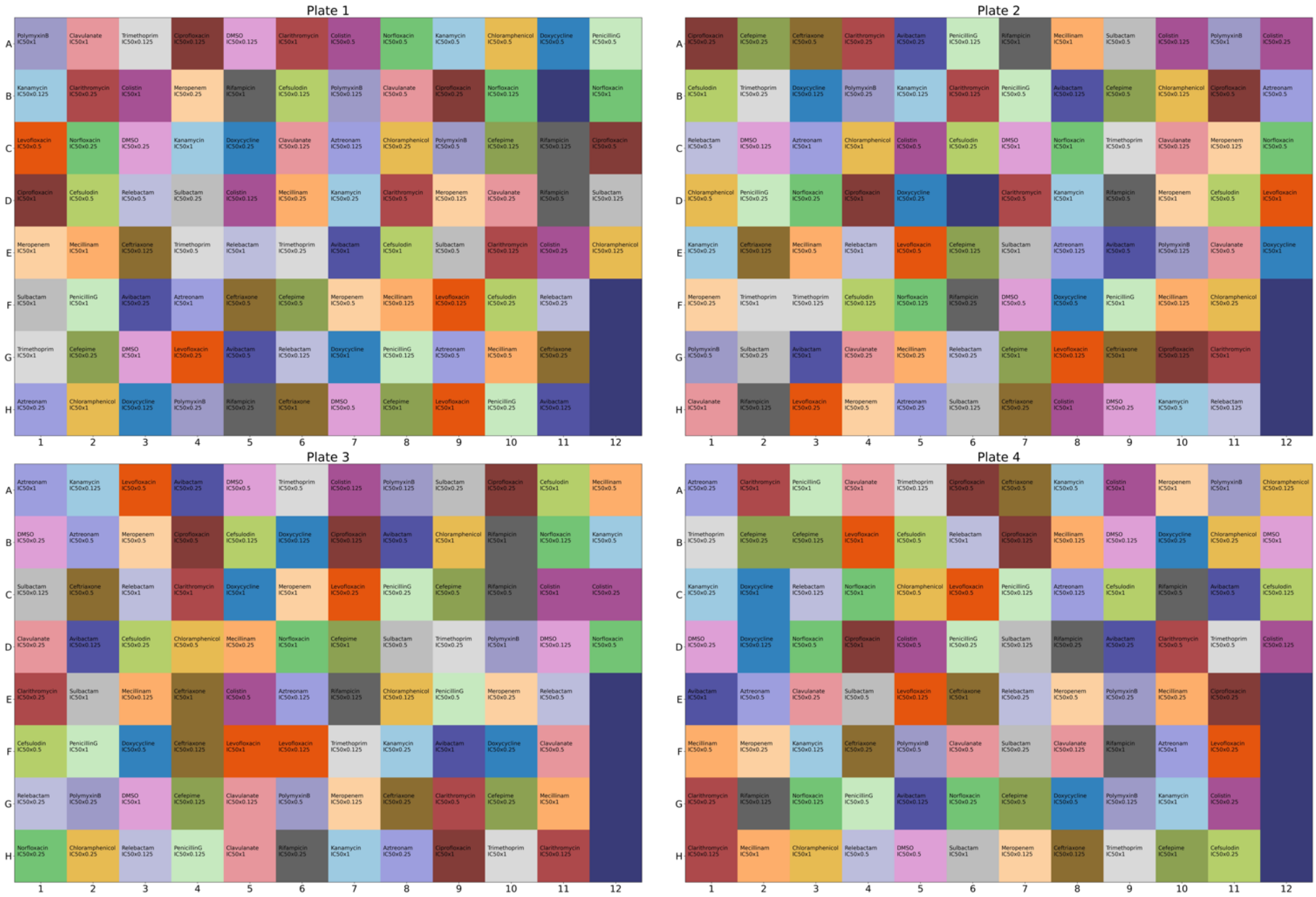

Supplementary Figure 1: Pseudo-randomised plate layouts.

Each 96-well plate of bacteria was acquired using independently cultured *E. coli* (biological replicates) and pseudo-block-randomised plate layouts[1] were generated for compound distribution with an acoustic liquid handler at four different concentrations. Pseudo-randomisation was used to decorrelate the treatment conditions from the positions on the plate and alleviate potential confounding effects. Colours in the plate maps correspond to individual antibiotics.

[1] https://github.com/crukci-bioinformatics/PlateLayout

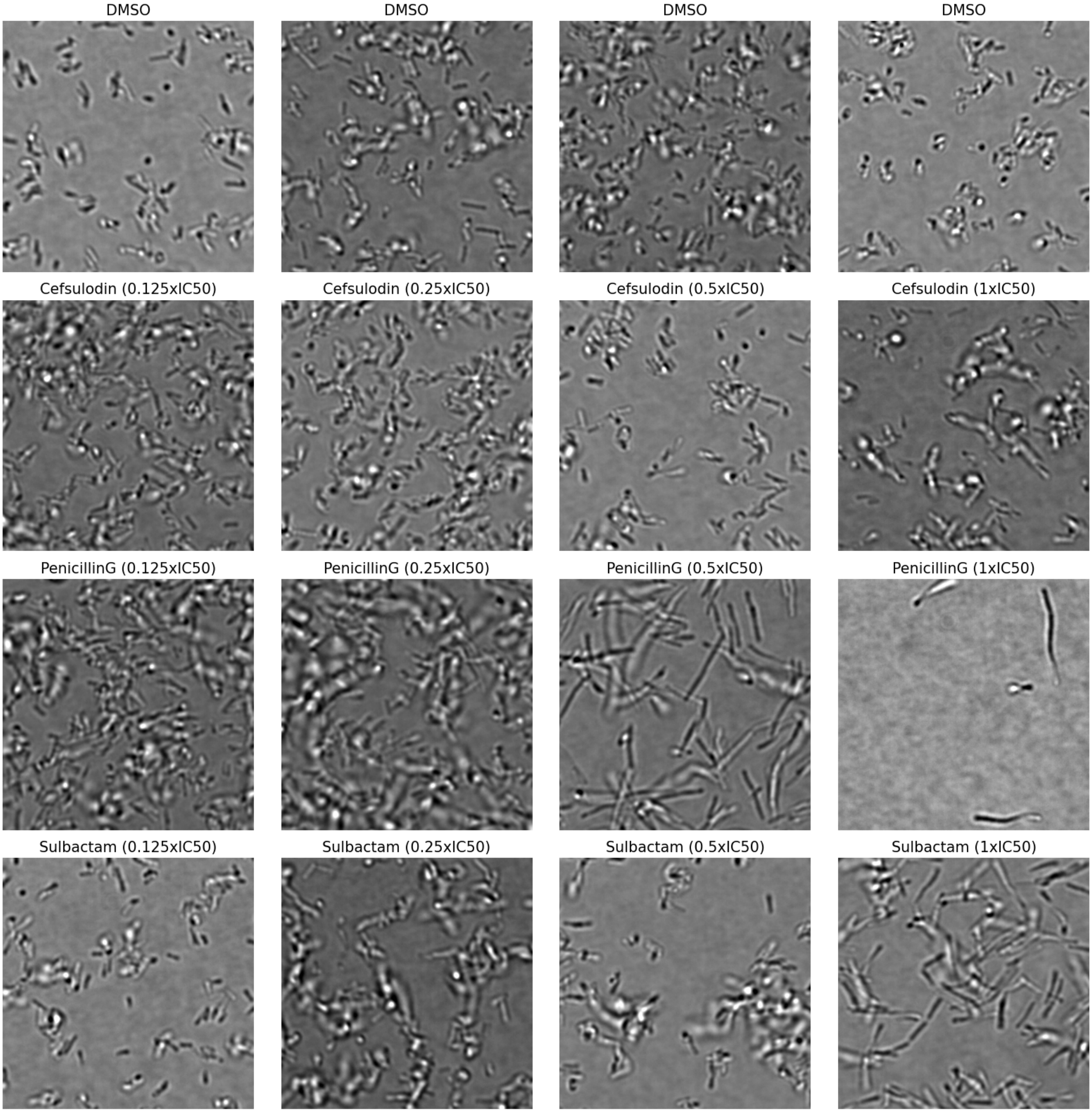

Supplementary Figure 2: Images of *E. coli* treated with PBP1A/B inhibitors.

Supplementary Figures 2-7 show randomly chosen and centre-cropped (64.5 × 64.5μm) brightfield images of *E. coli* bacteria left untreated (top row: DMSO) or treated with the indicated compound at the indicated concentration and grouped by MoA: PBP1A/B (this figure), PBP2 (Supplementary Fig. 3), PBP3 (Supplementary Fig. 4), gyrase (Supplementary Fig. 5), ribosome (Supplementary Fig. 6), membrane integrity, RNA polymerase and DNA synthesis (Supplementary Fig. 7).

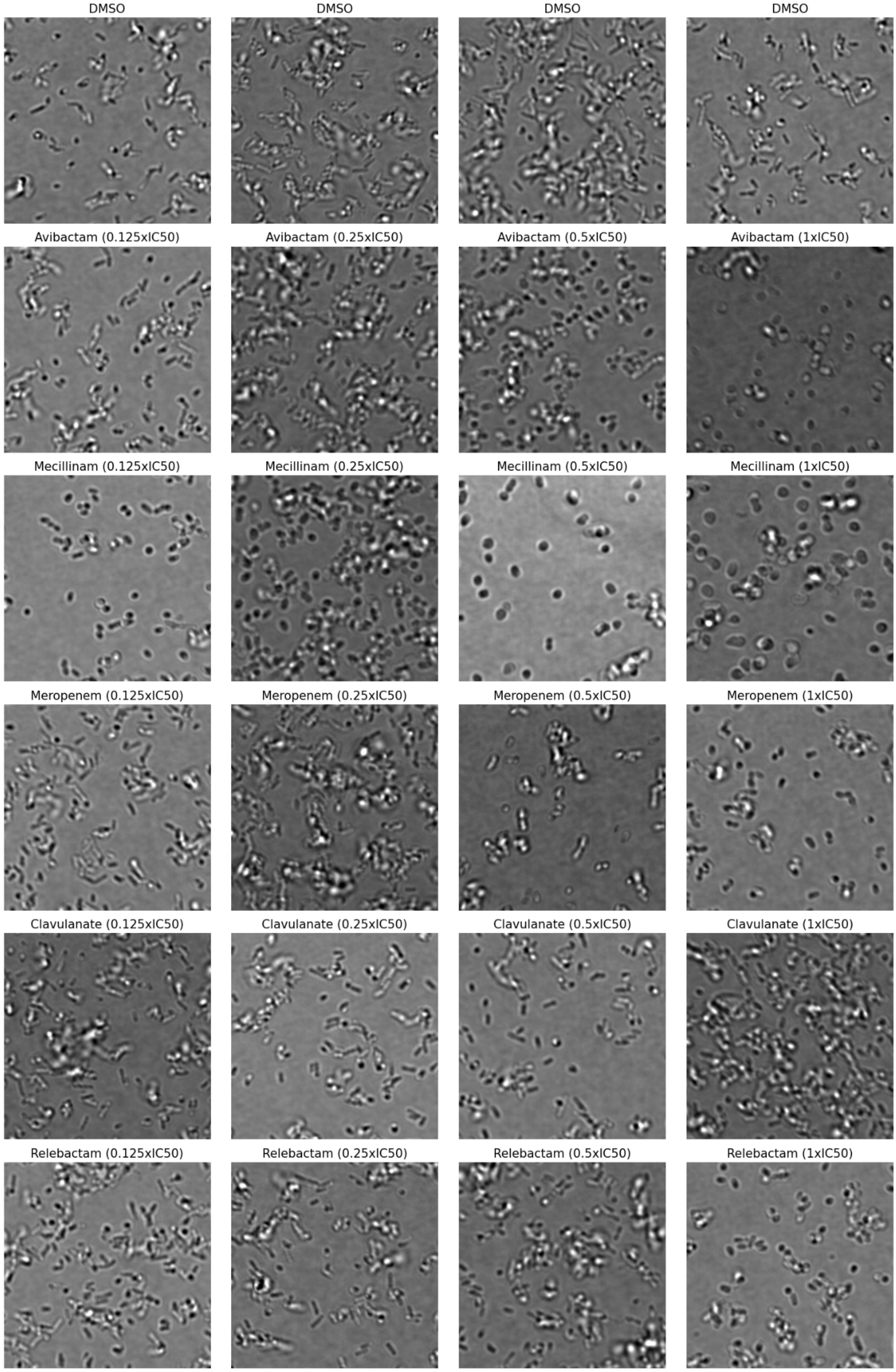

Supplementary Figure 3: Images of *E. coli* treated with PBP2 inhibitors.

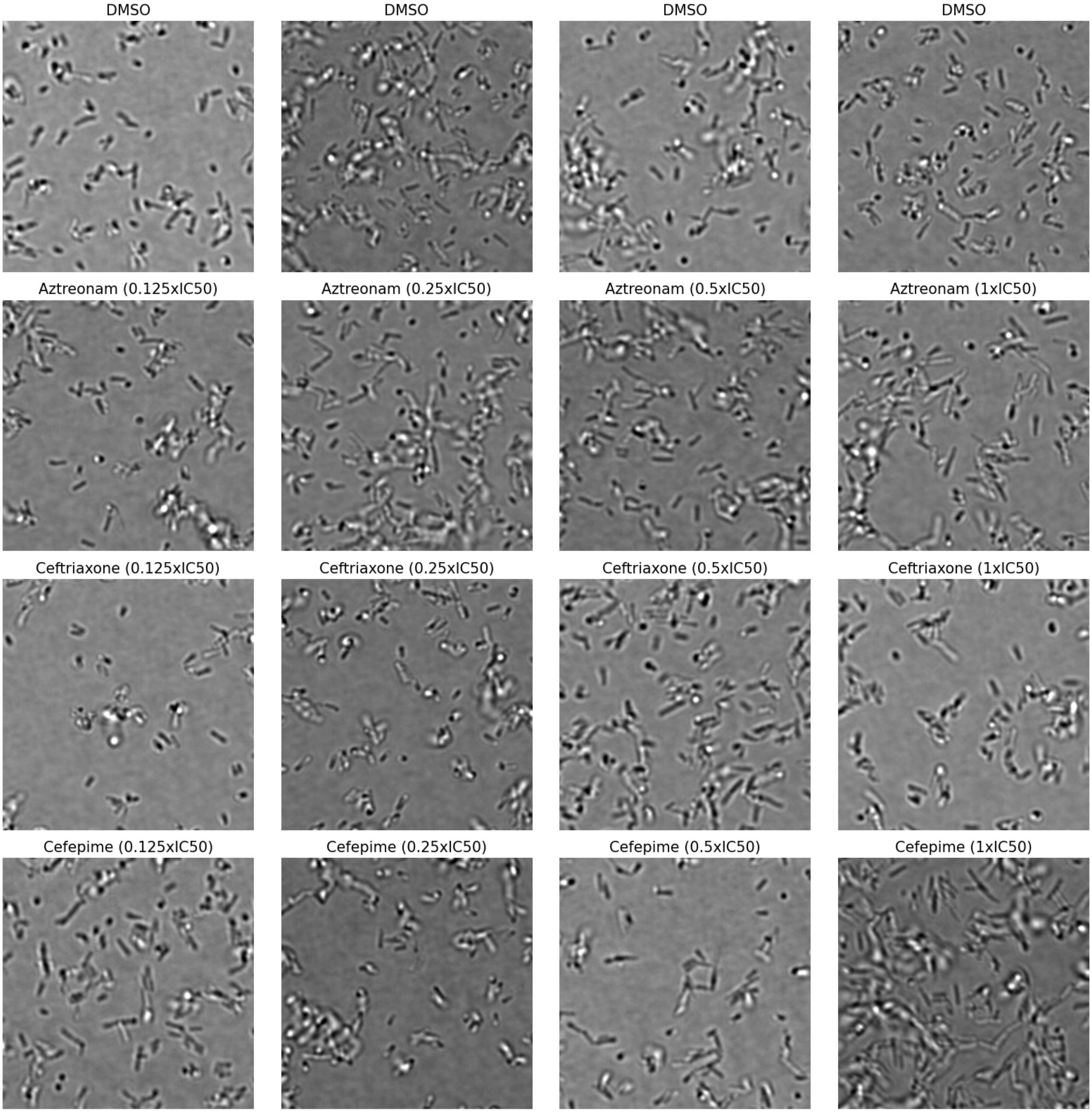

Supplementary Figure 4: Images of *E. coli* treated with PBP3 inhibitors.

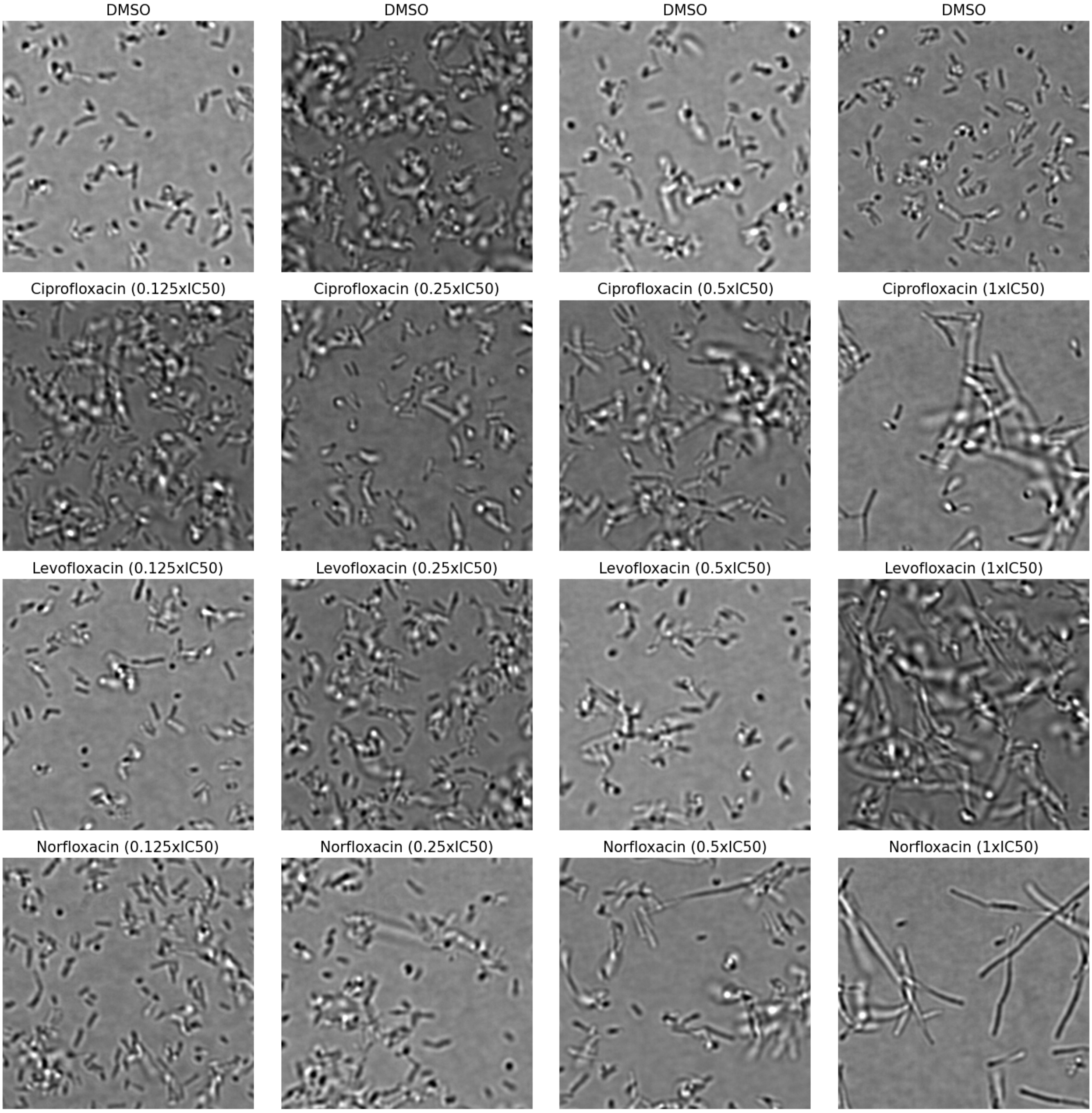

Supplementary Figure 5: Images of *E. coli* treated with DNA gyrase inhibitors.

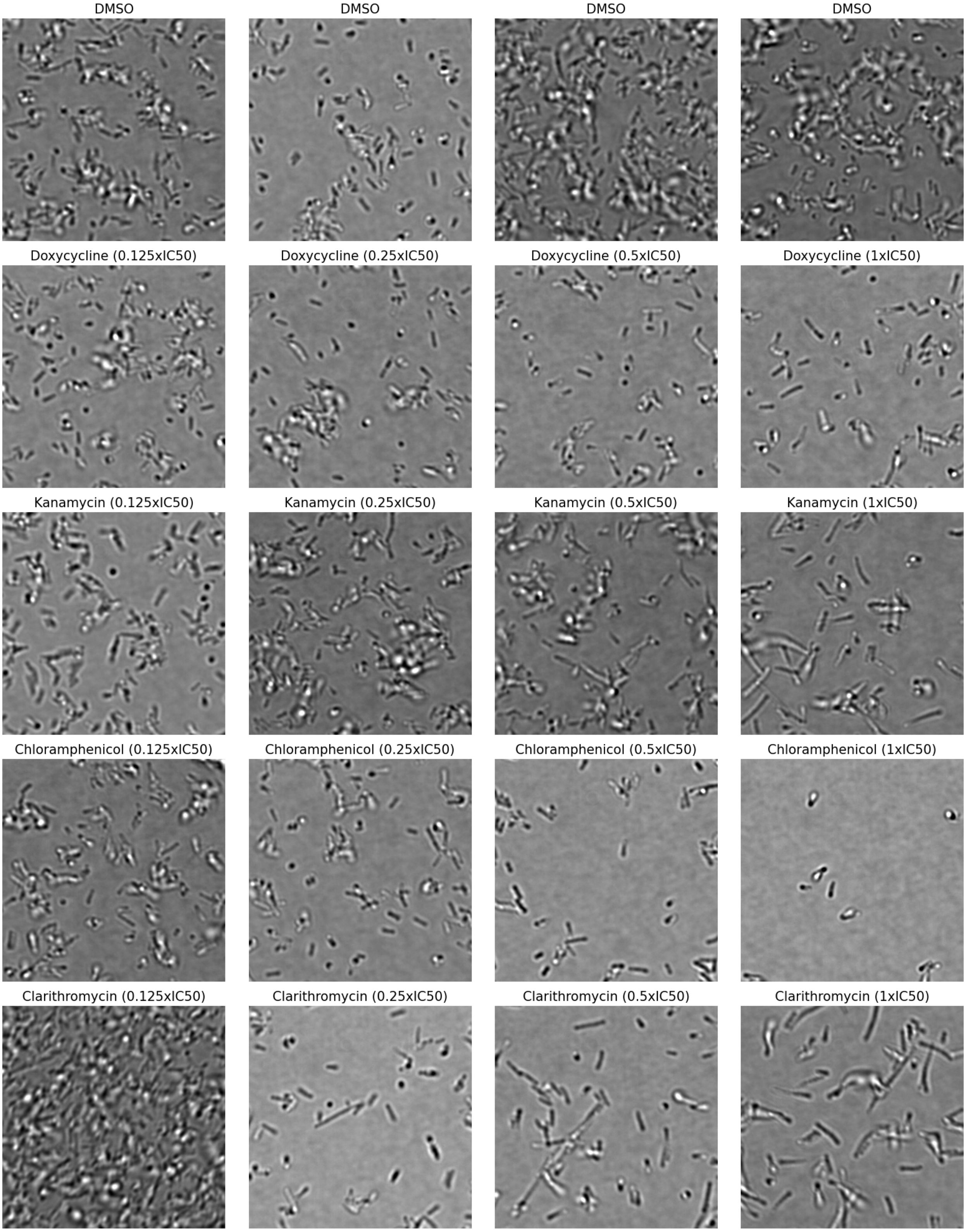

Supplementary Figure 6: Images of *E. coli* treated with ribosome inhibitors.

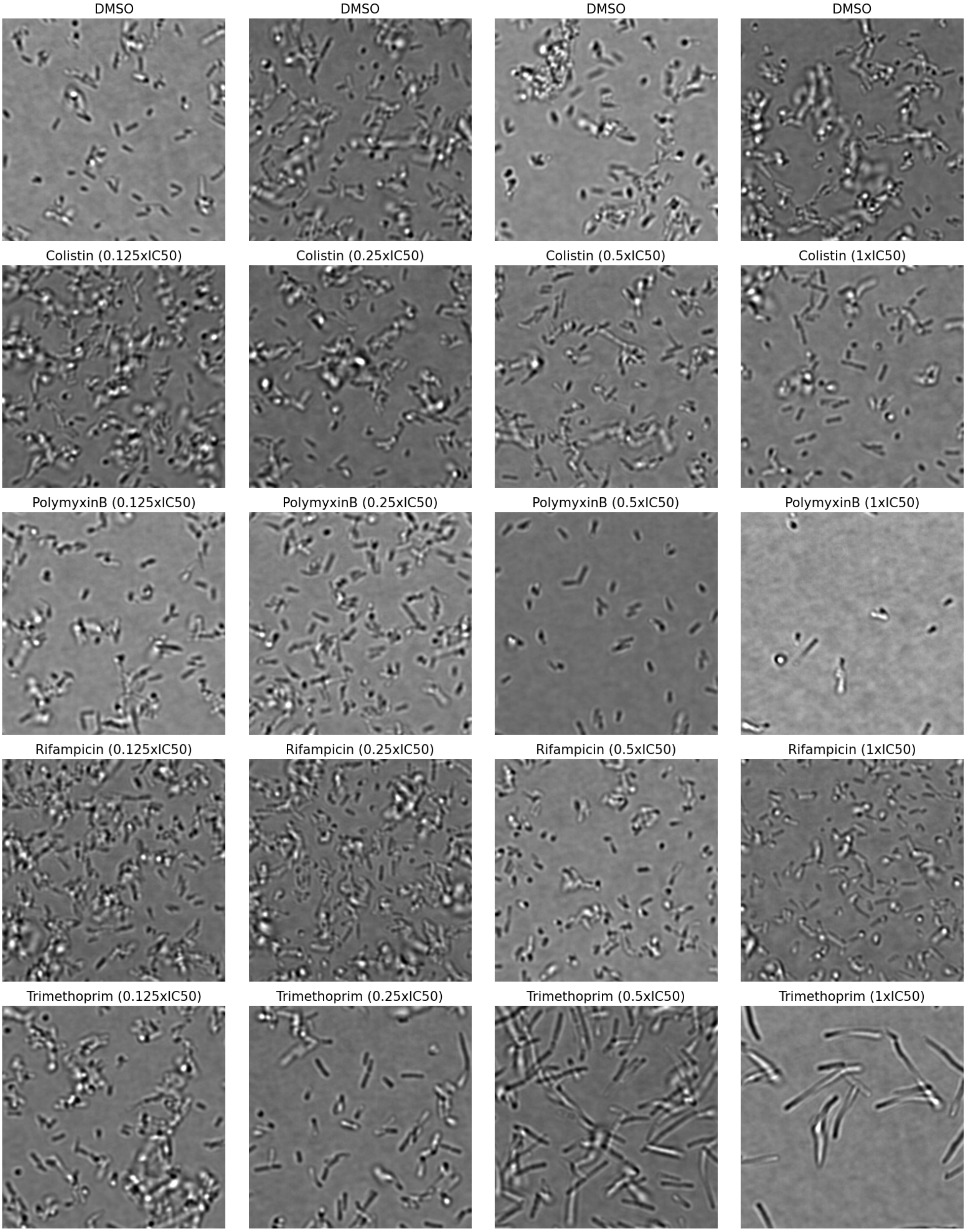

Supplementary Figure 7: Images of *E. coli* treated with membrane integrity, RNA polymerase and DNA synthesis inhibitors.

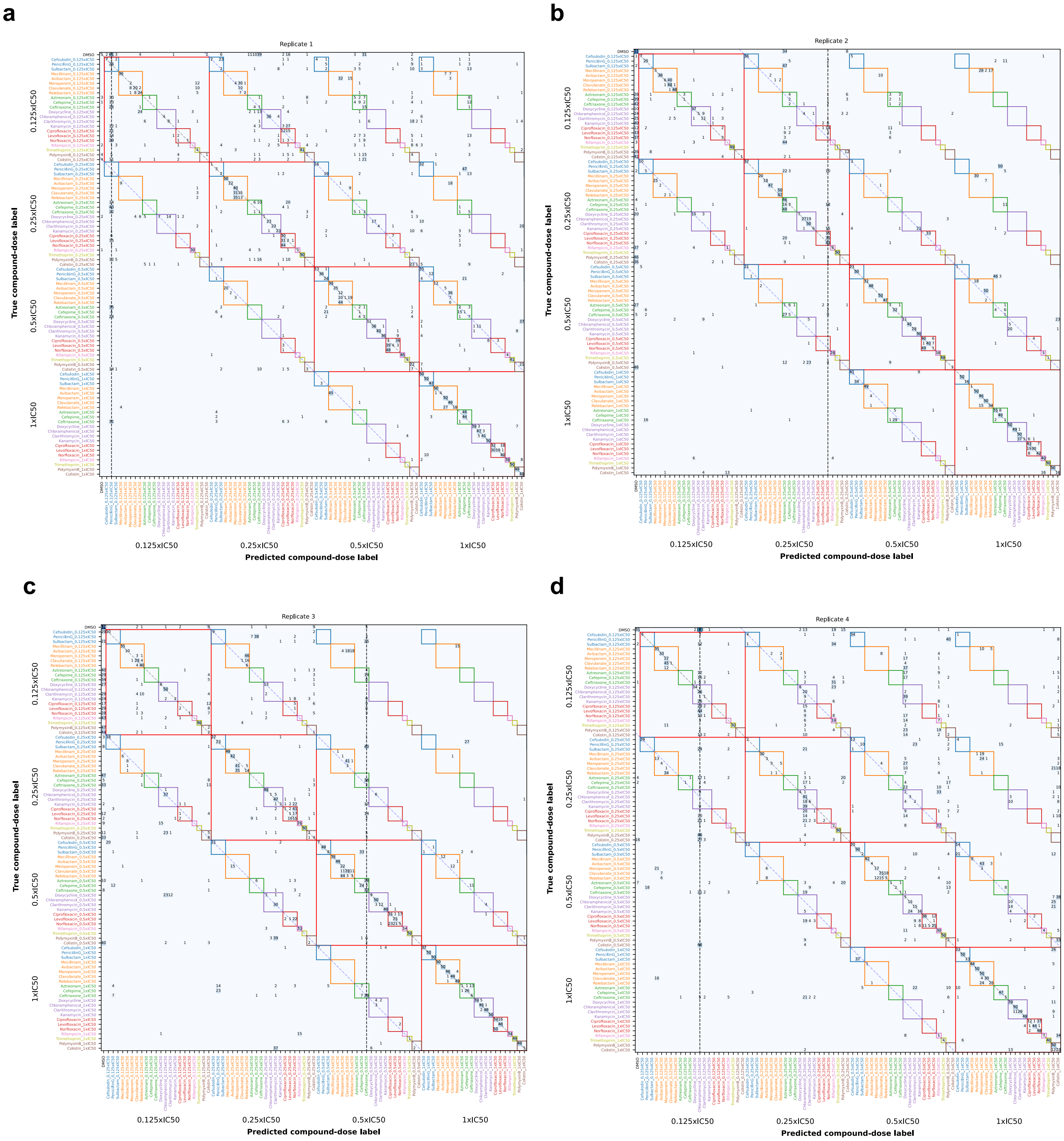

Supplementary Figure 8: Confusion matrices for treatment classification from *E. coli* images. The 89×89 confusion matrices show the predicted treatment combinations (compound identity + concentration = 22 × 4 combinations + 1 negative control condition, DMSO) compared to the ground-truth for CNNs trained on three plates and tested on a fourth hold-out test plate (all plates are biological replicates), in four rotations of the test plate (replicates 1-4). Only non-zero values are shown. Label colors indicate drug MoA as in Figs. 2b,c. Red boxes correspond to ground-truth-prediction pairs within the same relative concentration level (0.125, 0.25, 0.5 or 1×IC_{50}). Black boxes correspond to ground-truth-prediction pairs within the same MoA. Blue dashed diagonals correspond to confusions of concentration only (i.e. the predicted compound is correct). Vertical dashed lines highlight how multiple treatments can be confused with a single treatment. FoV-level classification accuracies and fold improvement over random are as follows: **(a)** Plate 1: 34.8% (31-fold). **(b)** Plate 2: 47.4% (42-fold). **(c)** Plate 3: 45.2% (40-fold). **(d)** Plate 4: 31.3% (28-fold). Source data are provided as a Source Data file.

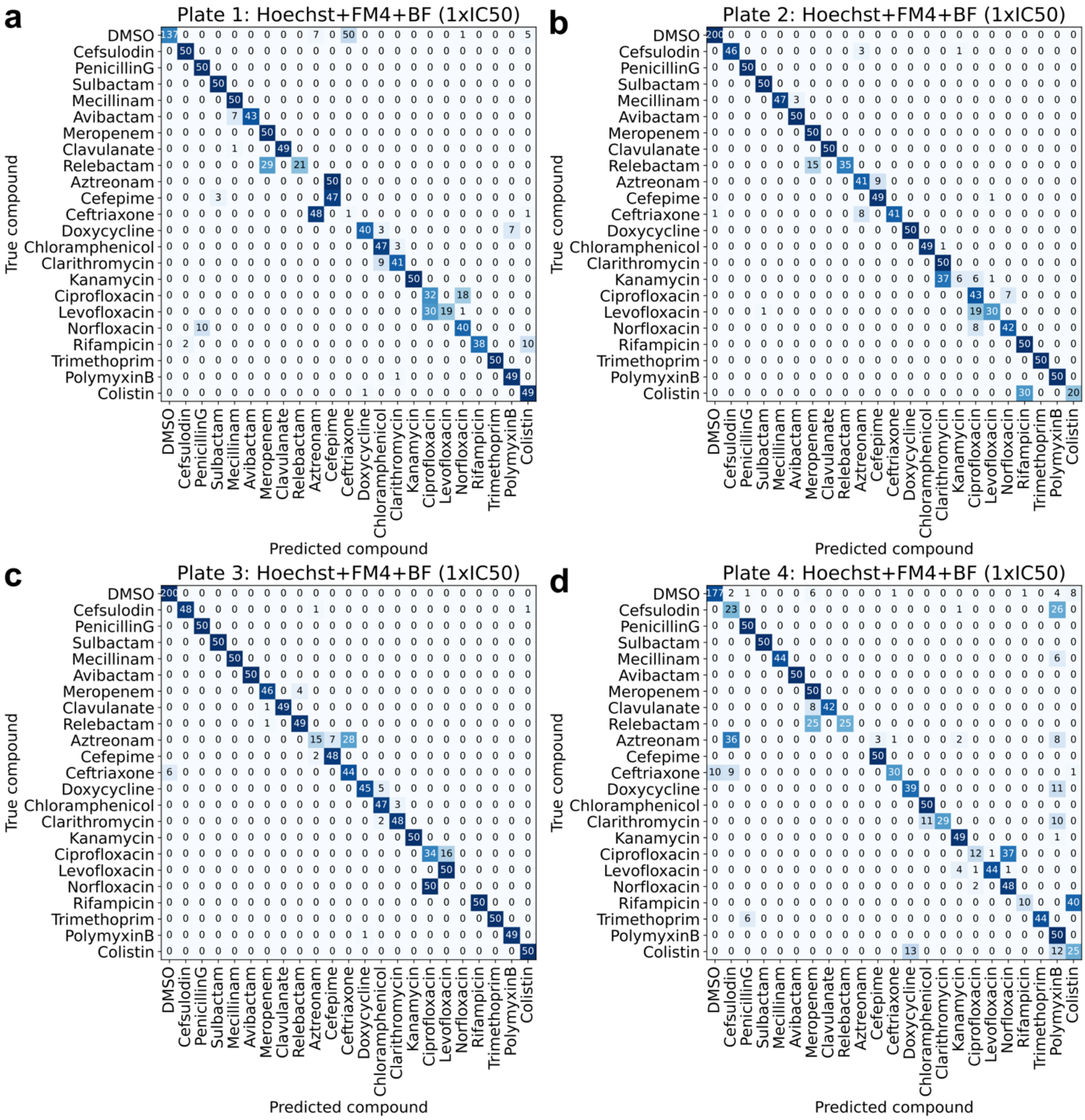

Supplementary Figure 9: Confusion matrices for drug recognition in *E. coli.* evaluated at IC_{50}. These 23×23 confusion matrices show the predicted compound compared to the ground-truth for CNNs trained on three plates and tested on treatments at the highest concentration ($1xIC_{50}$) in the fourth (hold-out) plate (all plates are biological replicates). In the test plate, compounds were administered at IC_{50} concentration. FoV-level classification accuracies for the four test plates are as follows: (**a**) Plate 1: 77.15%. (**b**) Plate 2: 88.38%. (**c**) Plate 3: 90.15%. (**d**) Plate 4: 76.23%. Source data are provided as a Source Data file.

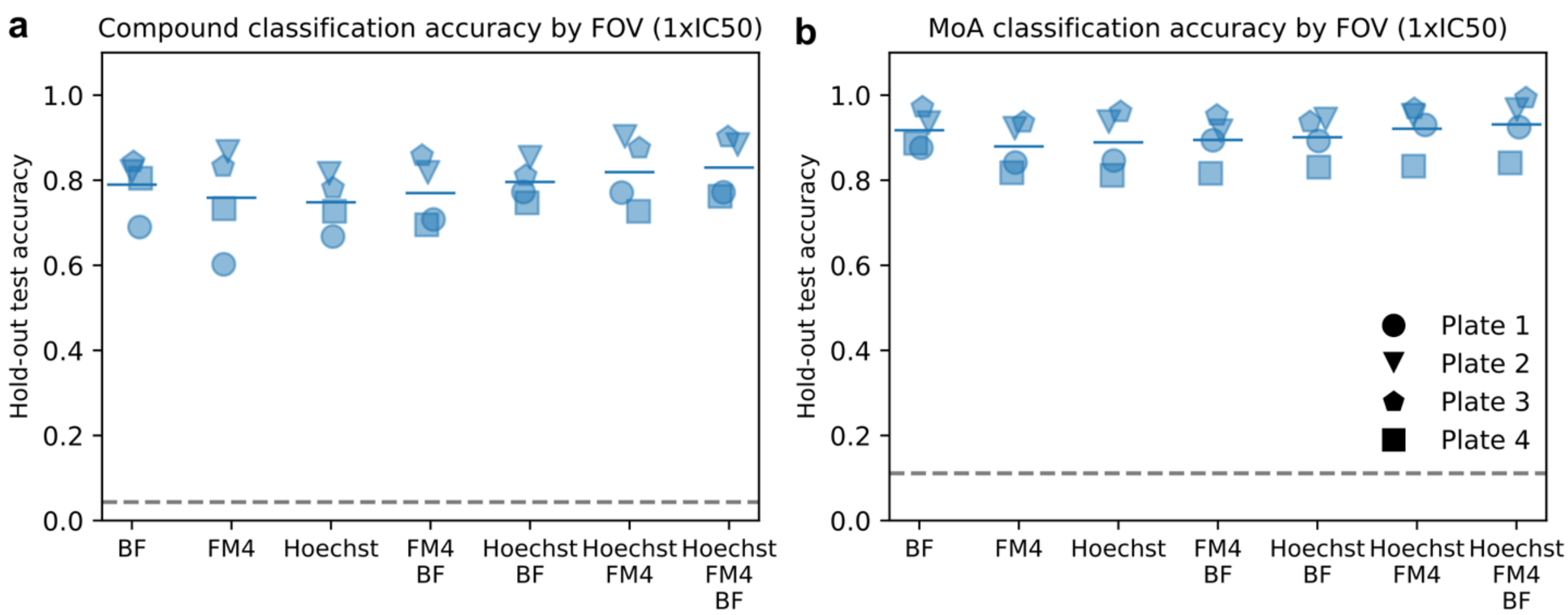

Supplementary Figure 10: Per-image classification accuracy for different input channel combinations.

Models were trained in a cross-validation experiment on data from three plates using different combinations of imaging channels (BF, FM4, Hoechst) as input and tested on treatments at the highest concentration (1xIC_{50}) in a hold-out test plate with the same combinations of channels. All plates are biological replicates. Plots show the average per-image classification accuracy for compounds **(a)** and MoAs **(b)**. Each symbol corresponds to a distinct plate and horizontal lines show median values. The dashed line indicates random classification. A Friedman test (n=4 replicates, k=7 channel combinations) showed no significant differences between channel combinations for compound classification accuracy (p=0.0638) but a significant difference for MoA classification accuracy (p=0.0165). Nevertheless, two-sided post-hoc Wilcoxon-rank sum tests showed no significant difference between any pair of channel combinations (p>=0.12 for all pairs, without adjusting for multiple comparisons, p>=0.26 after Benjamini-Hochberg adjustment for multiple comparisons). Source data are provided as a Source Data file.

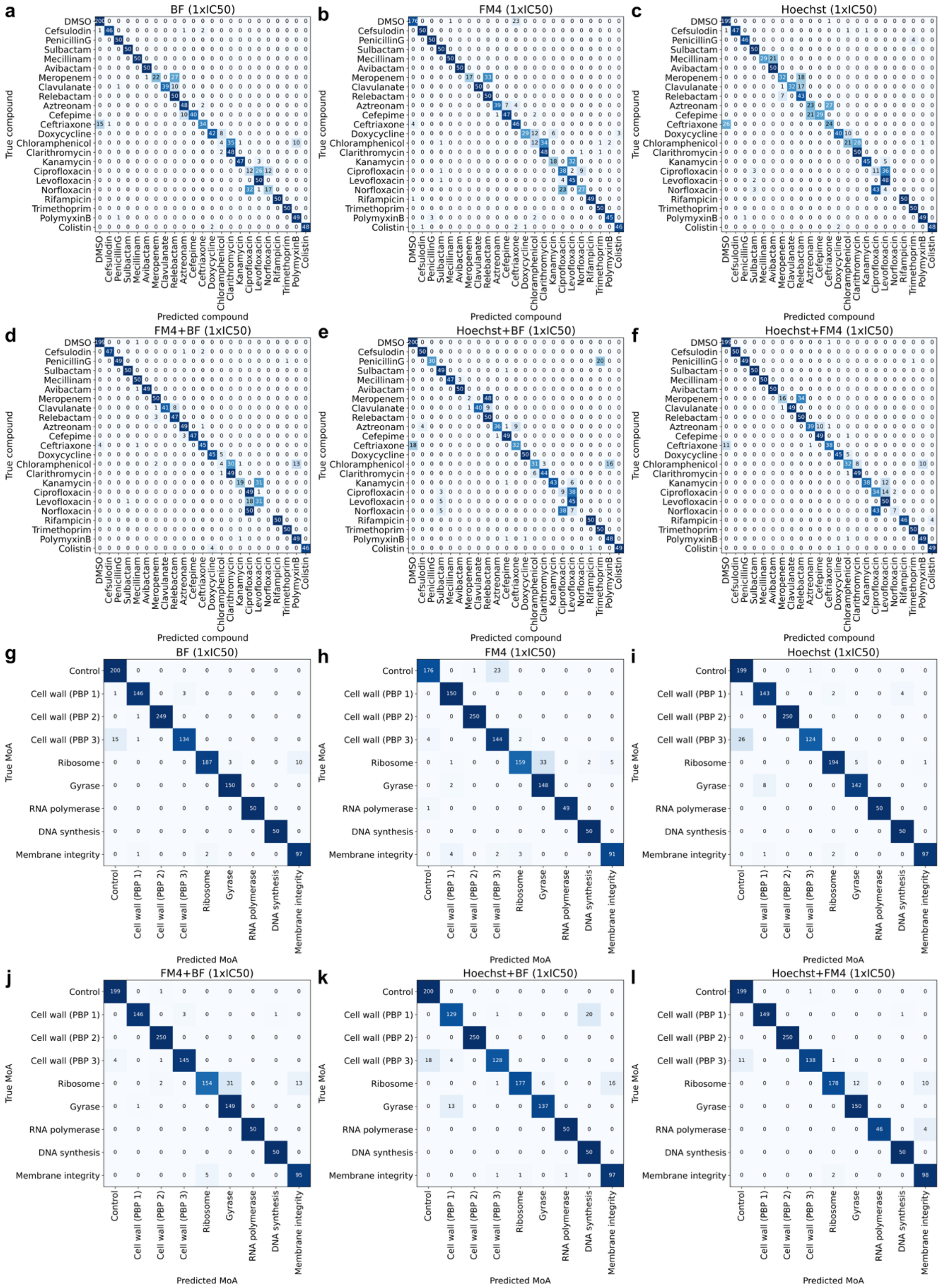

Supplementary Figure 11: Confusion matrices for different channel combinations at IC_{50}.
Confusion matrices by compound **(a-f)** and by MoA **(g-l)** are shown for different input channel combinations as indicated on top of each matrix. Here, confusion matrices for only one hold-out test plate are shown. Models were trained in a cross-validation experiment with three plates using one or two imaging channels in different combinations as input and tested on a hold-out test plate at IC_{50} with the same imaging channels. Corresponding confusion matrices for a model trained and tested on all three colour channels (using the same hold-out test plate) are shown in Fig. 2b and 2c. All plates are biological replicates. Source data are provided as a Source Data file.

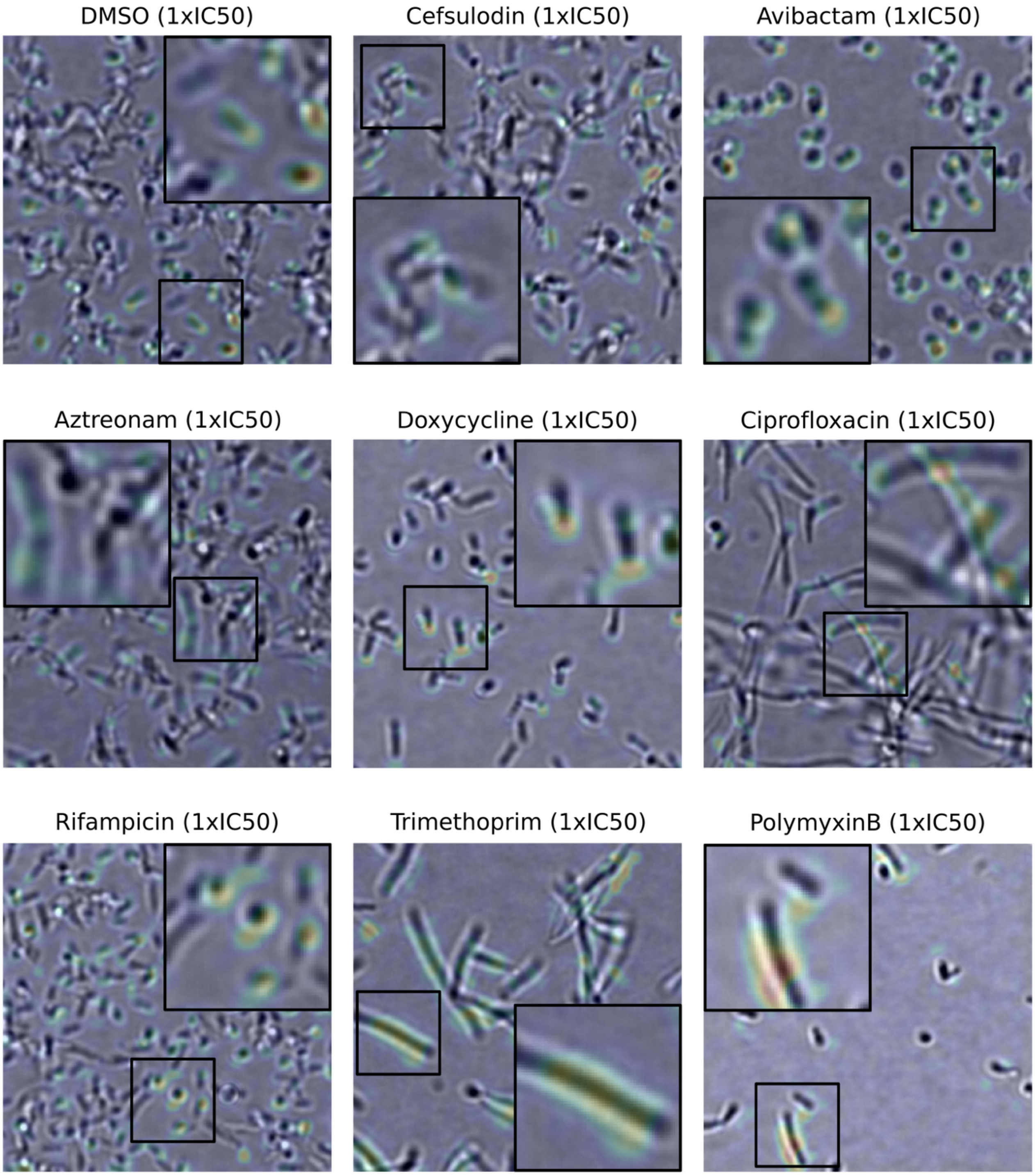

Supplementary Figure 12: Saliency maps highlight bacterial features.
The nine panels show examples of brightfield images (grey-scale) of *E. coli* bacteria treated with the indicated antibiotic (or the negative control, DMSO), with a superposed Grad-CAM activation heatmap (coloured). For each antibiotic, the shown Grad-CAM corresponds to the ground-truth class, e.g. for the bottom right image the Grad-CAM corresponds to the class 'PolymyxinB'. Images are of size 32.25 × 32.25 μm and insets are of size 8.38 × 8.38 μm.

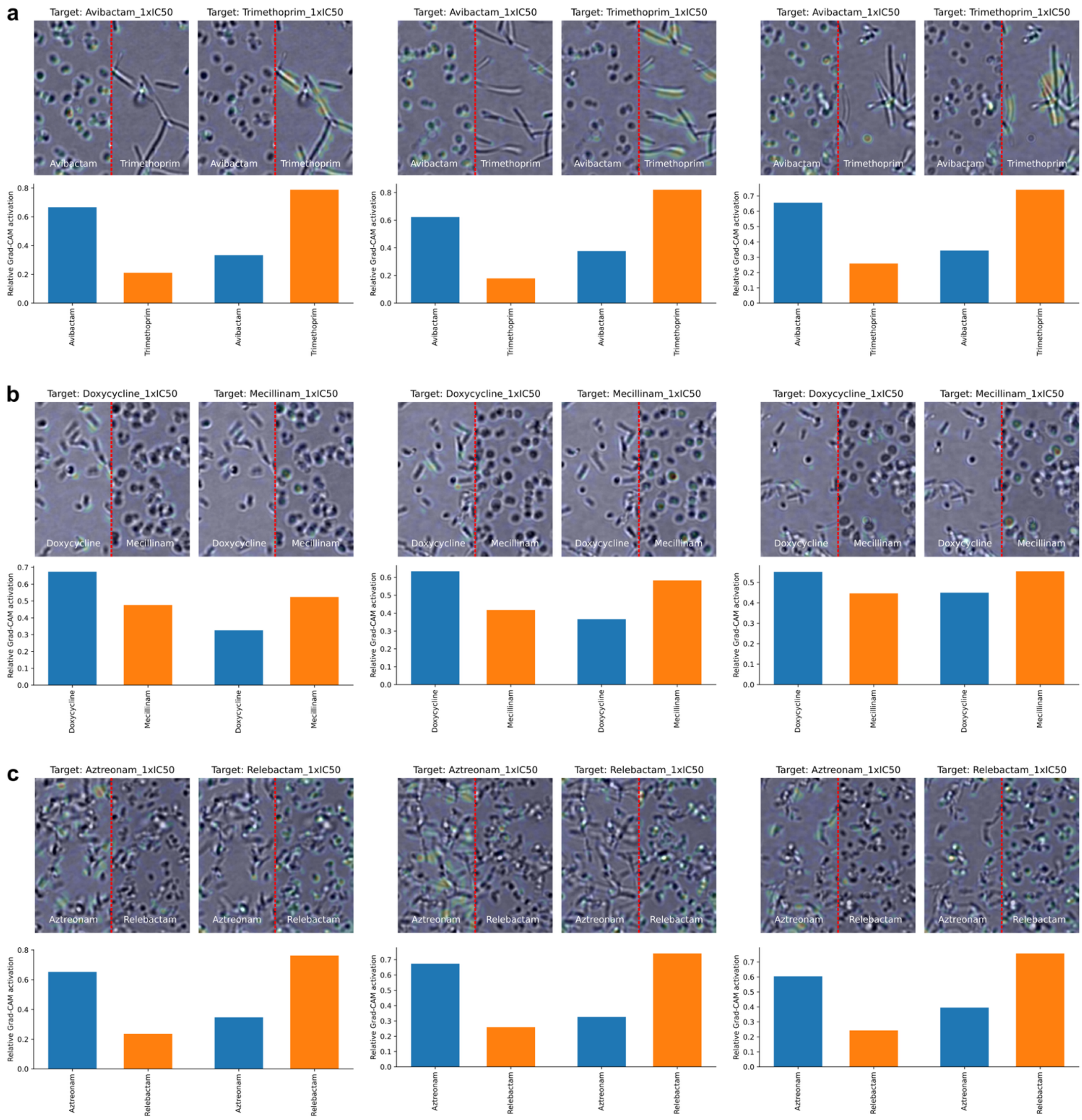

Supplementary Figure 13: Counterfactual saliency maps and quantification of relative activation.

Top rows in panels a-c show composite images where the left half is a brightfield image of *E. coli* bacteria treated with one antibiotic and the right half is a brightfield image of *E. coli* bacteria treated with another antibiotic. The antibiotics are indicated in white on the bottom. The composite images were passed to the CNN and the Grad-CAM activation maps were obtained for each of the antibiotic classes. Brightfield images are shown in grey, and the Grad-CAM is superposed as a coloured heat map, with the antibiotic class of the Grad-CAM indicated on top as 'Target'. Image size is 32.25×32.25 μm. Bar plots in the bottom rows show relative Grad-CAM activations computed as described in 'Methods'. For example, the relative activations for the left-hand-side panel in **a** were obtained (from left to right) as $R^{\text{Trimethoprim}}_{\text{Avibactam}}(\text{Avibactam})$, $R^{\text{Trimethoprim}}_{\text{Avibactam}}(\text{Trimethoprim})$, $R^{\text{Avibactam}}_{\text{Trimethoprim}}(\text{Avibactam})$ and $R^{\text{Avibactam}}_{\text{Trimethoprim}}(\text{Trimethoprim})$. Bars for the same antibiotic (colour) sum to 1. **(a)** Avibactam vs. trimethoprim, **(b)** doxycycline vs. mecillinam. **(c)** aztreonam vs. relebactam. Source data are provided as a Source Data file.

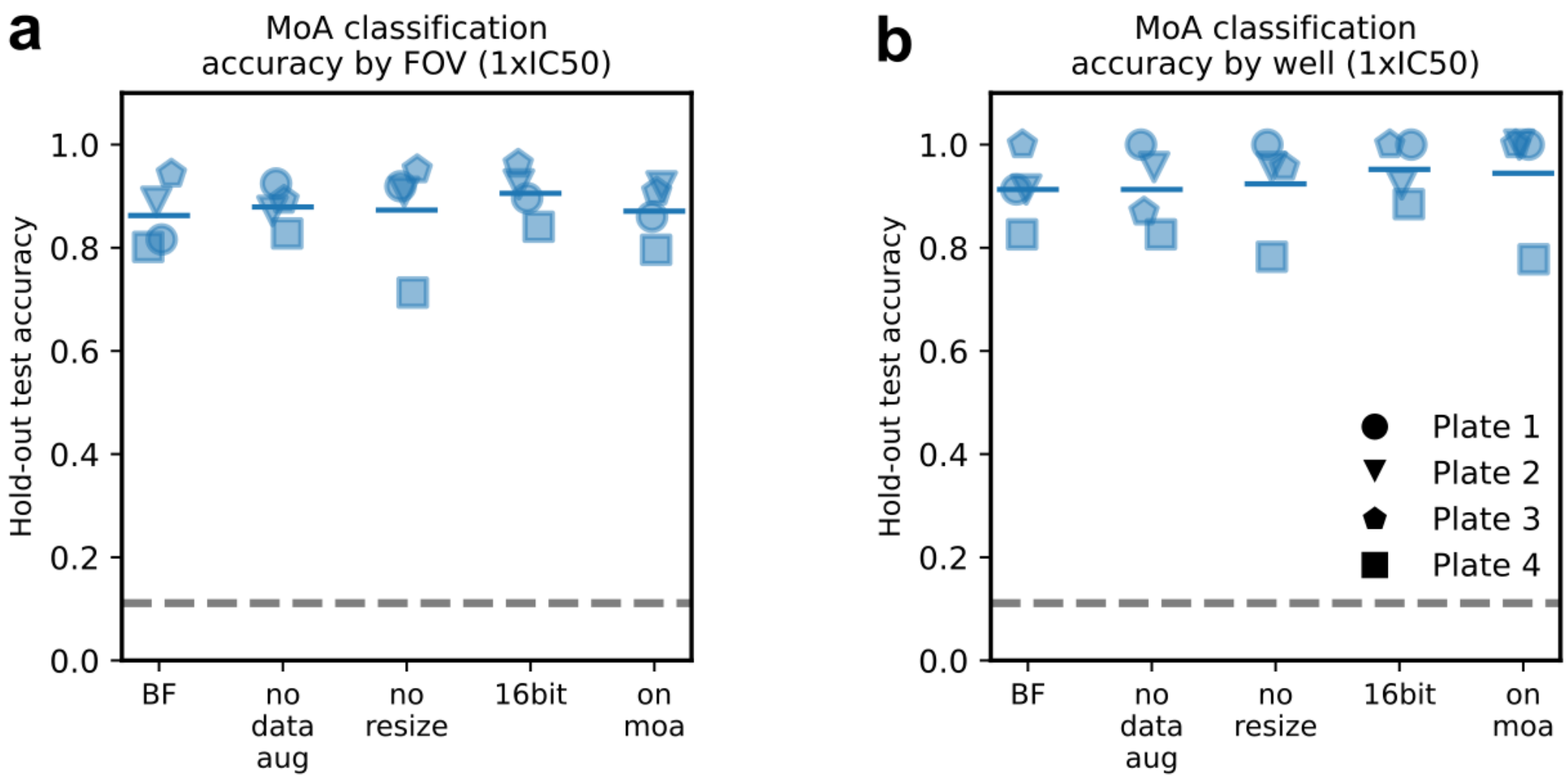

Supplementary Figure 14: MoA recognition accuracy for different training settings.
Plots show mode of action (MoA) classification accuracies by FoV **(a)** and by well **(b)** for five different training settings. For each setting, four models were trained on data from three plates for 100 epochs and tested on the treatments at the highest concentration (1xIC_{50}) in the fourth hold-out plate. All plates are biological replicates. 'BF': reference implementation with data augmentation, 2×2 image down-sampling, 8-bit image format, without MoA labels; 'no data aug.': models trained without data augmentation; 'no resize': models trained on full resolution images without down-sampling; '16bit': models trained on images with full bit-depth; 'on moa': models directly trained on MoA labels instead of treatment conditions (drugs+concentrations). A Friedman test (n=4 replicates, k=5 training settings) showed no significant differences between the different training settings on MoA classification by FoV (p=0.287) or by well (p=0.736). Source data are provided as a Source Data file.

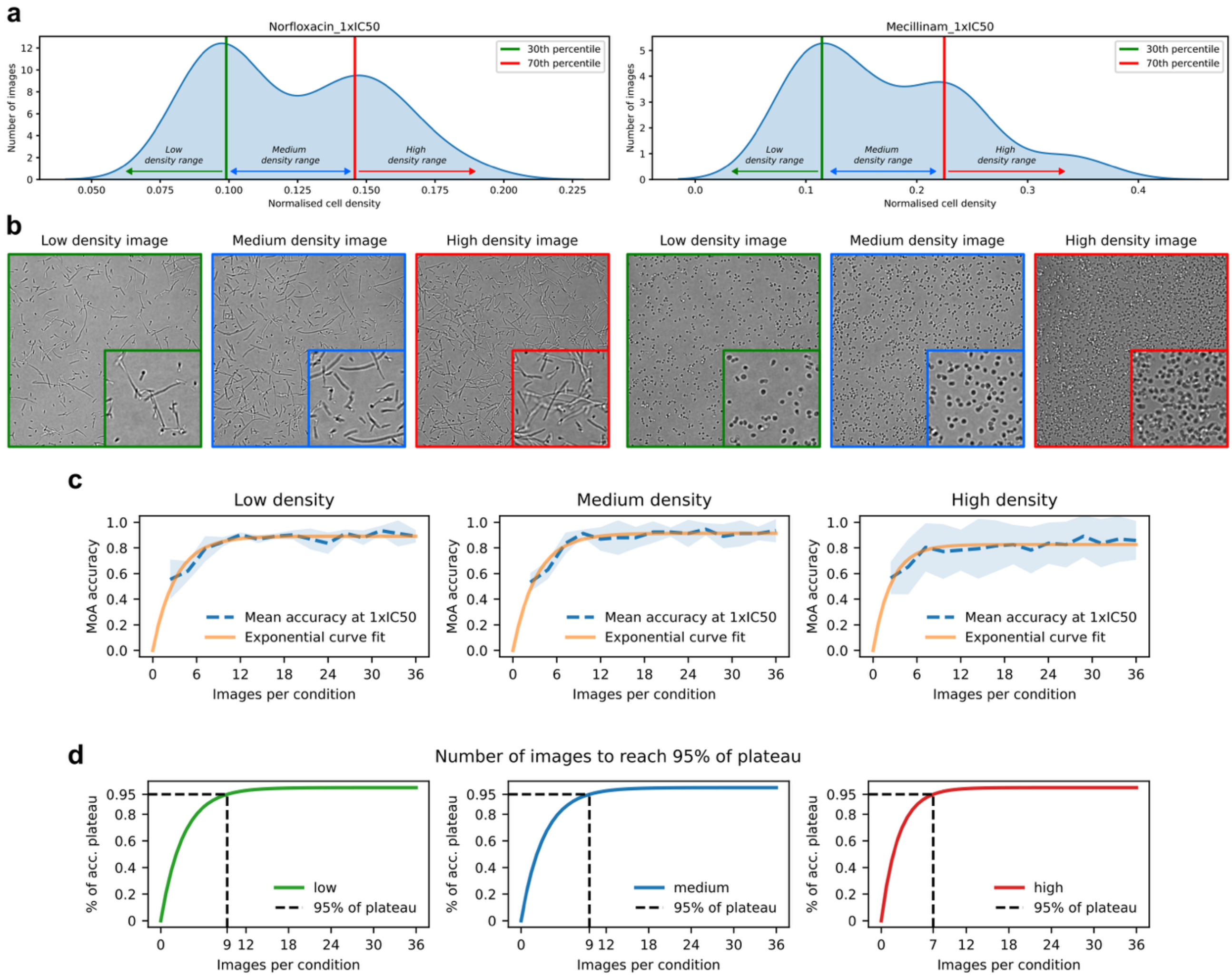

Supplementary Figure 15: Influence of cell density on required number of training images.
(a) Distribution of the normalised bacterial cell density across images (kernel density estimate) for two treatment conditions, calculated from the FM4-64FX channel as described in 'Methods'. For each treatment condition (compound + concentration), images were split into low, medium and high density based on the 30th and 70th percentile of the normalised density. **(b)** Example images with low (green), medium (blue) and high (red) bacterial density shown for norfloxacin (left) and mecillinam (right) at 1xIC_{50}. **(c)** Blue dashed curves show the average well-level MoA classification accuracy as function of the number of training images per condition with the shaded area indicating the standard deviation, using training images of different densities, and evaluated on bacteria treated at IC_{50}. The solid orange curve shows a fitted two-parameter exponential function and is used to calculate the number of images required to reach 95% of the maximum accuracy. **(d)** Curves show the fitted exponential and the number of images per condition required to reach 95% of the accuracy plateau for low, medium and high bacterial density, showing that between seven and nine images are sufficient. Source data are provided as a Source Data file.

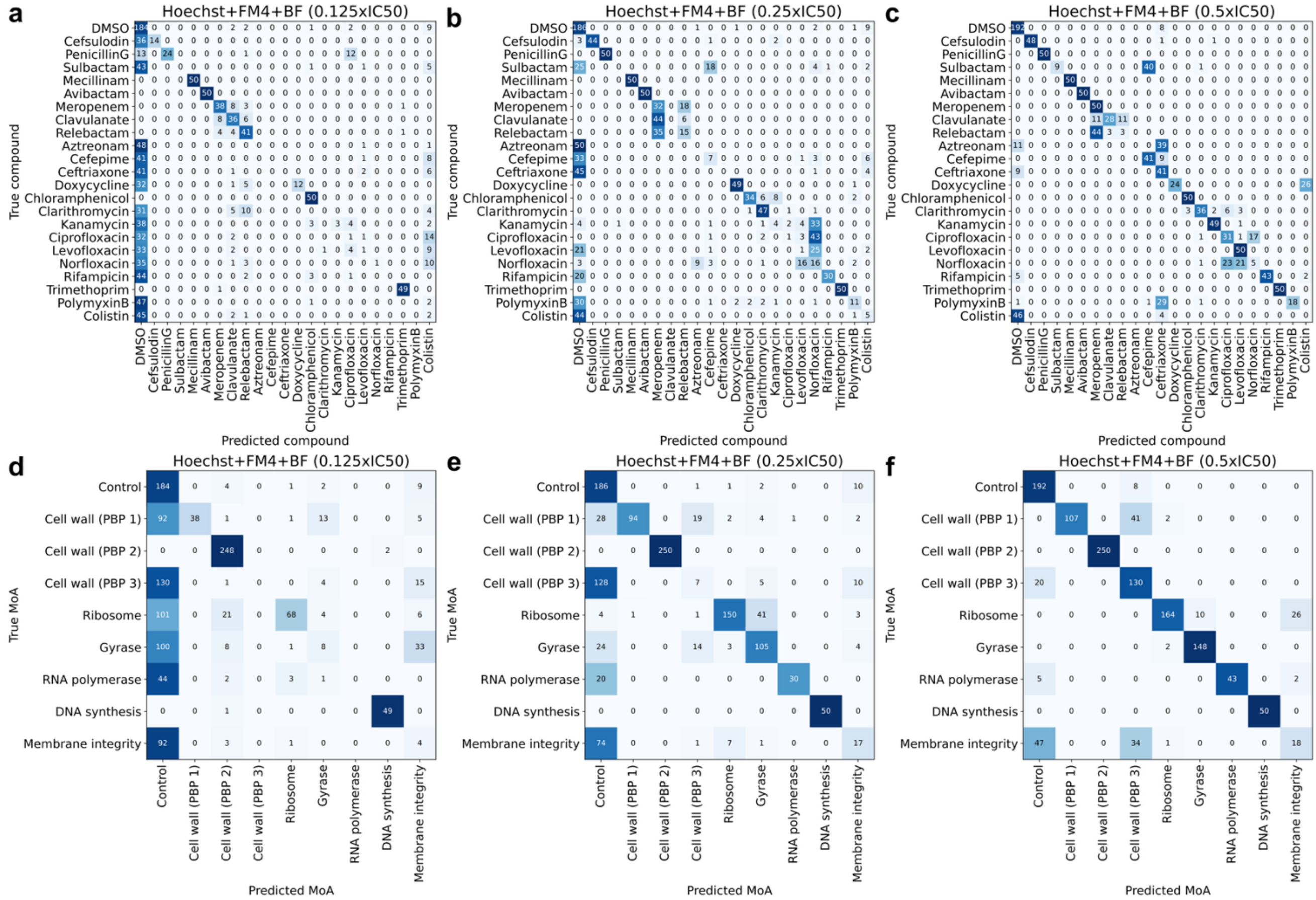

Supplementary Figure 16: Confusion matrices for sub-IC$_{50}$ concentrations.
Confusion matrices evaluated at three different sub-IC$_{50}$ concentrations (0.125×IC$_{50}$, 0.25×IC$_{50}$ and 0.5×IC$_{50}$) for compound prediction **(a-c)** and MoA prediction **(d-f)** show classification accuracy increasing with concentration. All three channels were used as input and the same model (trained on all compound-concentration combinations on three biological replicate plates) was used for all predictions. The same test plate (biological replicate) was used for all evaluations. Source data are provided as a Source Data file.

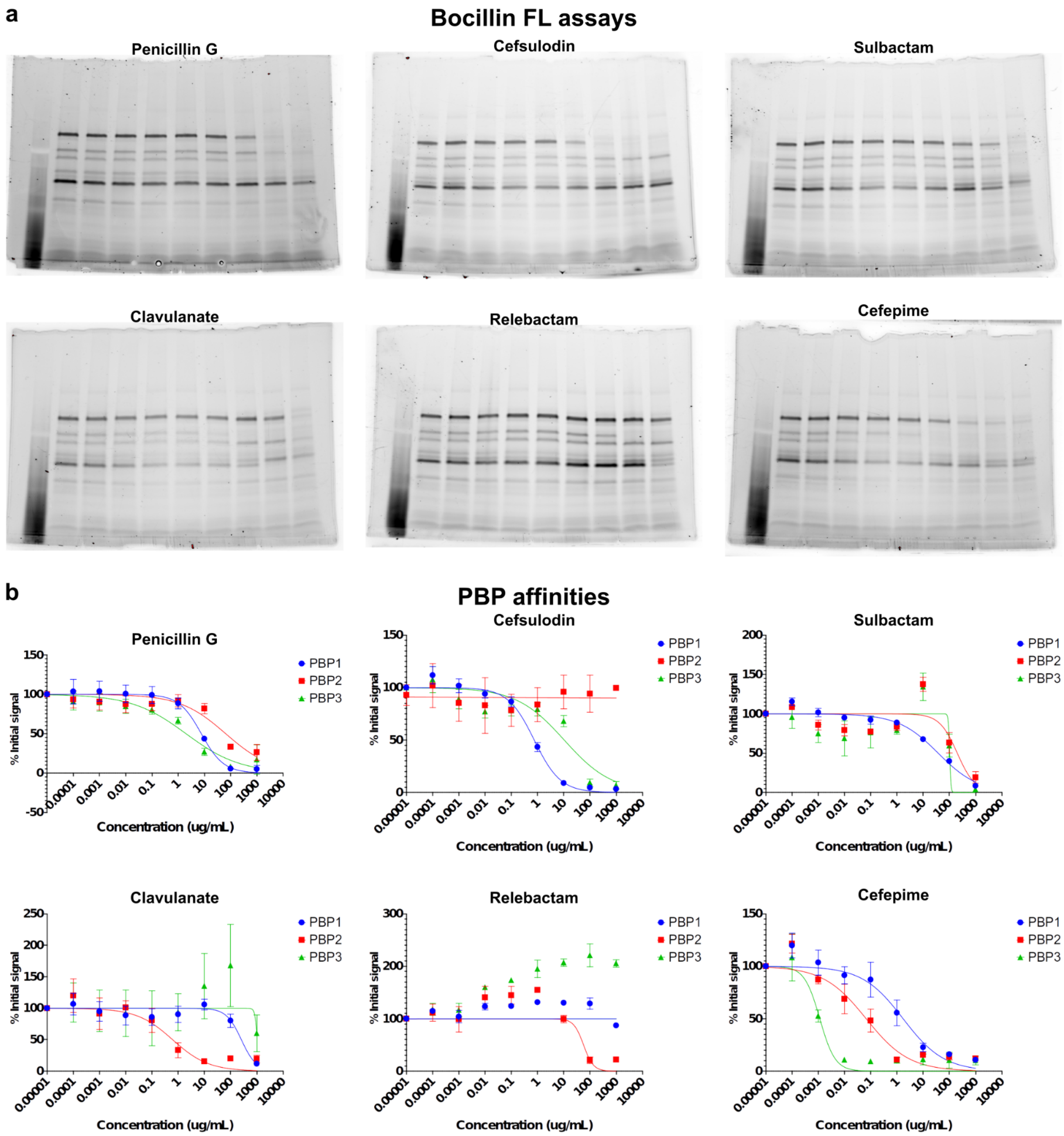

Supplementary Figure 17: Identifying PBP specificities with Bocillin FL competition assays. **(a)** The fluorescent penicillin analogue Bocillin FL labels PBPs in *E. coli* as resolved by SDS-PAGE. Using a competition assay against a dilution series of PBP inhibitors, changes to the Bocillin FL labelling of each individual PBP can be quantified to reveal the specificity of these inhibitors. **(b)** The quantified PBP bands were plotted against antibiotic concentrations. N=3 independent experiments were performed. Source data are provided as a Source Data file.

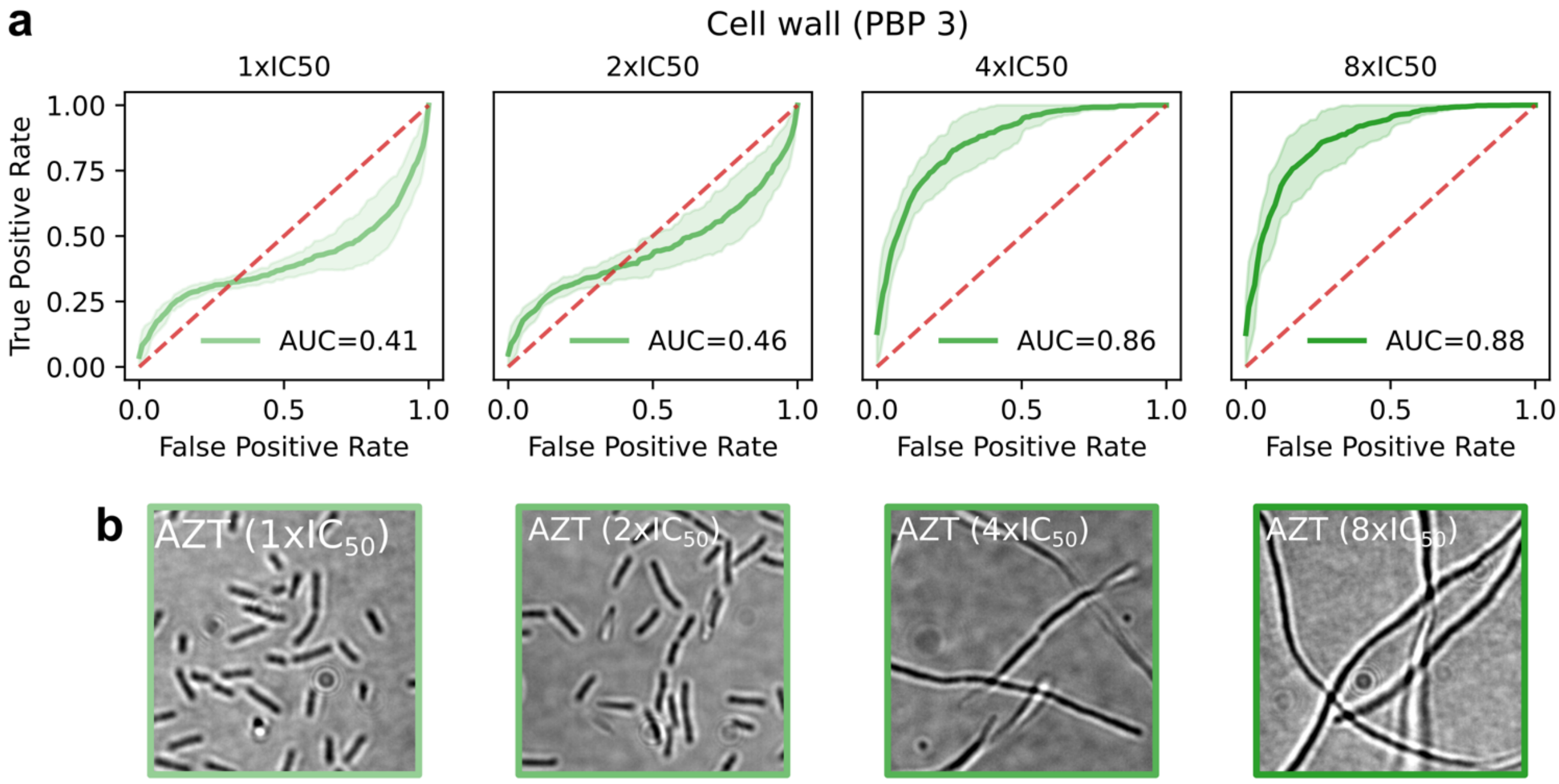

Supplementary Figure 18: PBP3 inhibitors are detected as novel MoAs at higher concentrations
For this figure, we repeated the MoA novelty detection analysis shown in Fig. 6 using the previously trained models to obtain embeddings from a new biological replicate, but using higher concentrations of the PBP3 inhibitors aztreonam, cefepime and ceftriaxone, ranging from $1\times IC_{50}$ to $8\times IC_{50}$. **(a)** Receiver operating characteristic (ROC) curves for PBP3 inhibitors evaluated on four hold-out test plates, as in Fig. 6e, but at concentrations of $1\times IC_{50}$, $2\times IC_{50}$, $4\times IC_{50}$ and $8\times IC_{50}$. Solid lines show averages and shaded areas the standard deviation across the four replicates. The mean area-under-the curve (AUC) is indicated. **(b)** Images of aztreonam-treated bacteria at these concentrations. A clear change in phenotype is apparent between $2\times IC_{50}$ and $4\times IC_{50}$. Source data are provided as a Source Data file.

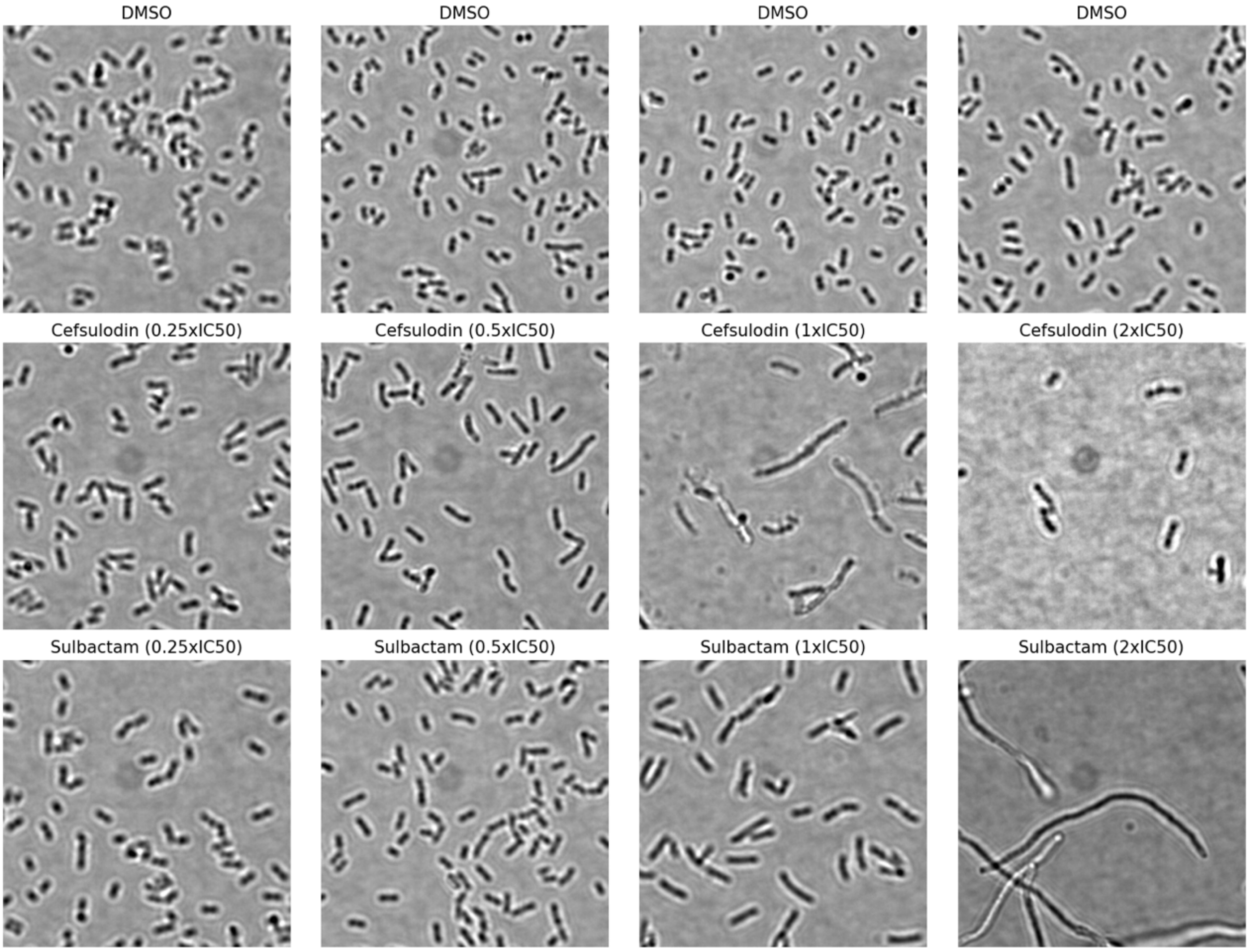

Supplementary Figure 19: Images of *K. pneumoniae* treated with PBP1A/B inhibitors.

Supplementary Figures 19-24 show randomly selected and centre-cropped (64.5 × 64.5μm) brightfield images of *K. pneumoniae* bacteria left untreated (DMSO negative control, top) or treated with the indicated compound at the indicated concentration (from 0.25xIC_{50} to 2xIC_{50}) and grouped by MoA: PBP1A/B (this figure), PBP2 (Supplementary Fig. 20), PBP3 (Supplementary Fig. 21), DNA gyrase (Supplementary Fig. 22), ribosome (Supplementary Fig. 23), membrane integrity, RNA polymerase and DNA synthesis (Supplementary Fig. 24).

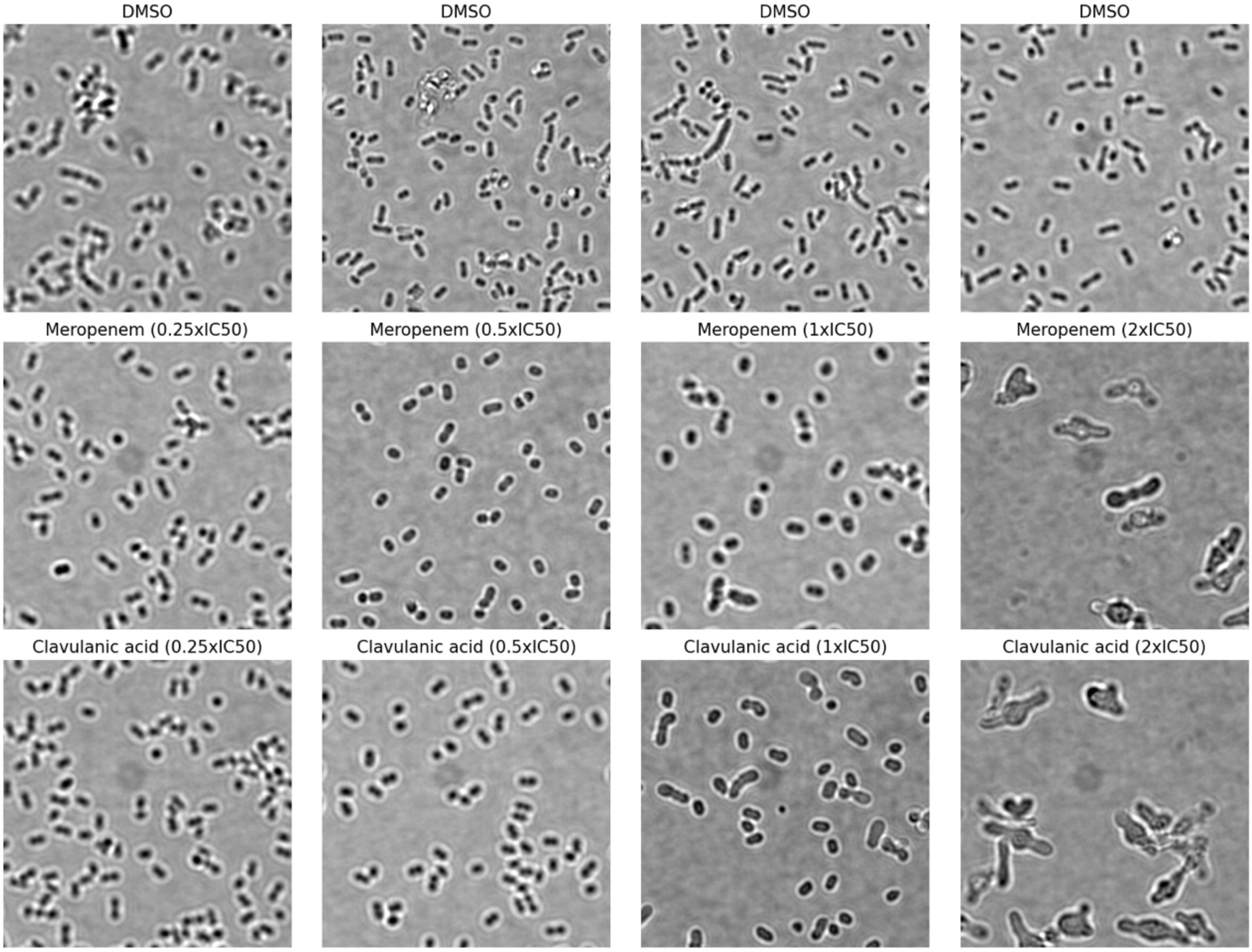

Supplementary Figure 20: Images of *K. pneumoniae* treated with PBP2 inhibitors.

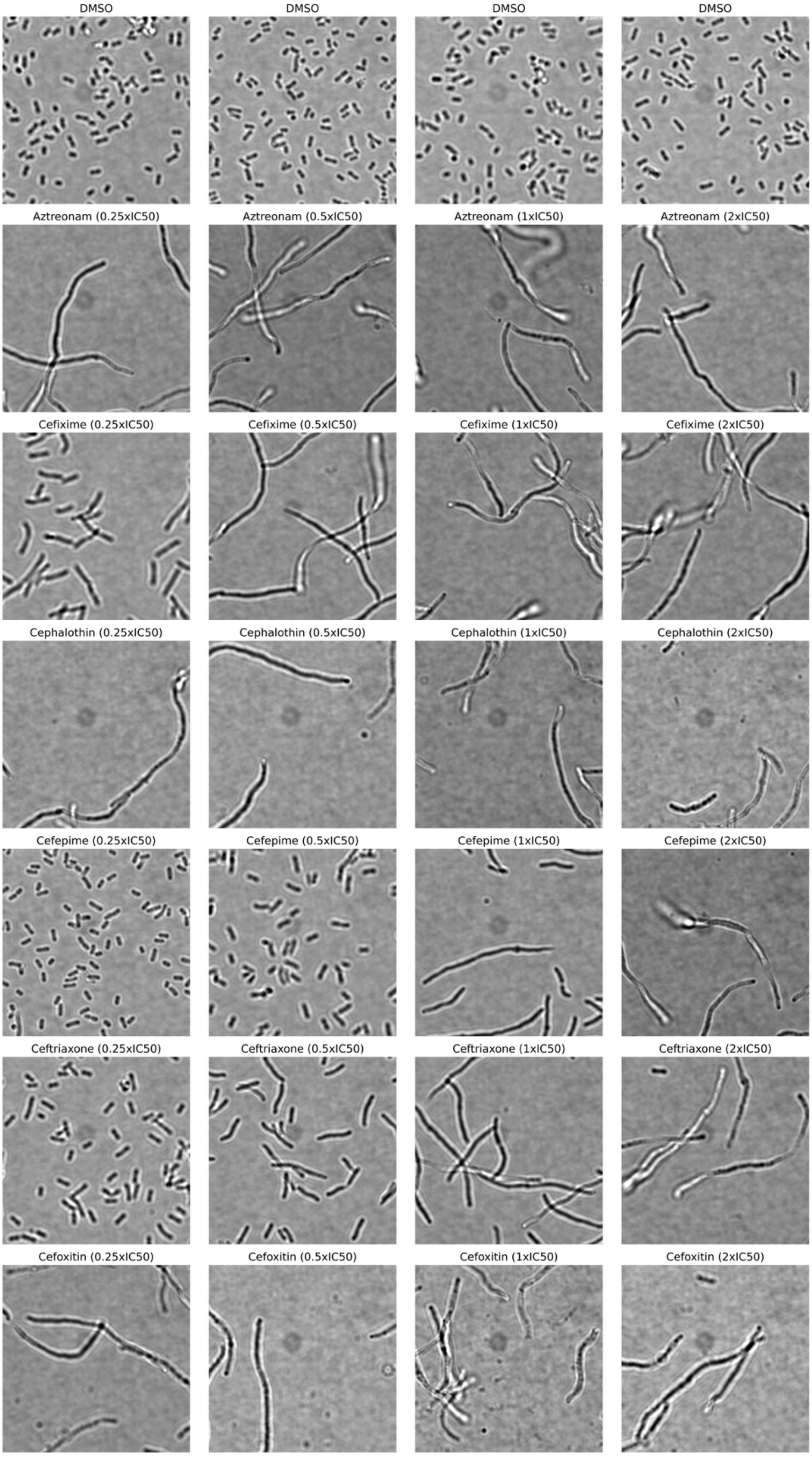

Supplementary Figure 21: Images of *K. pneumoniae* treated with PBP3 inhibitors.

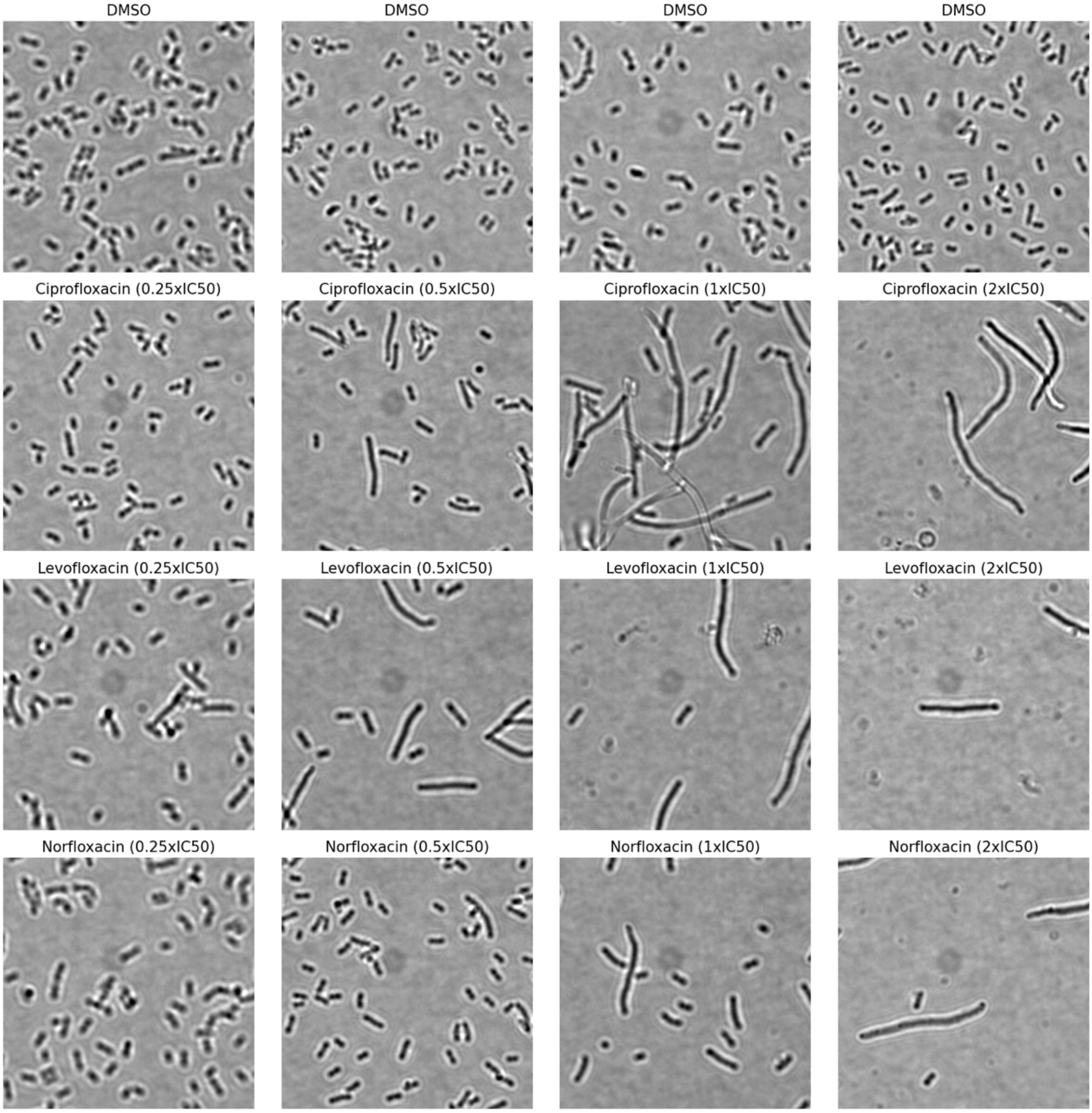

Supplementary Figure 22: Images of *K. pneumoniae* treated with DNA gyrase inhibitors.

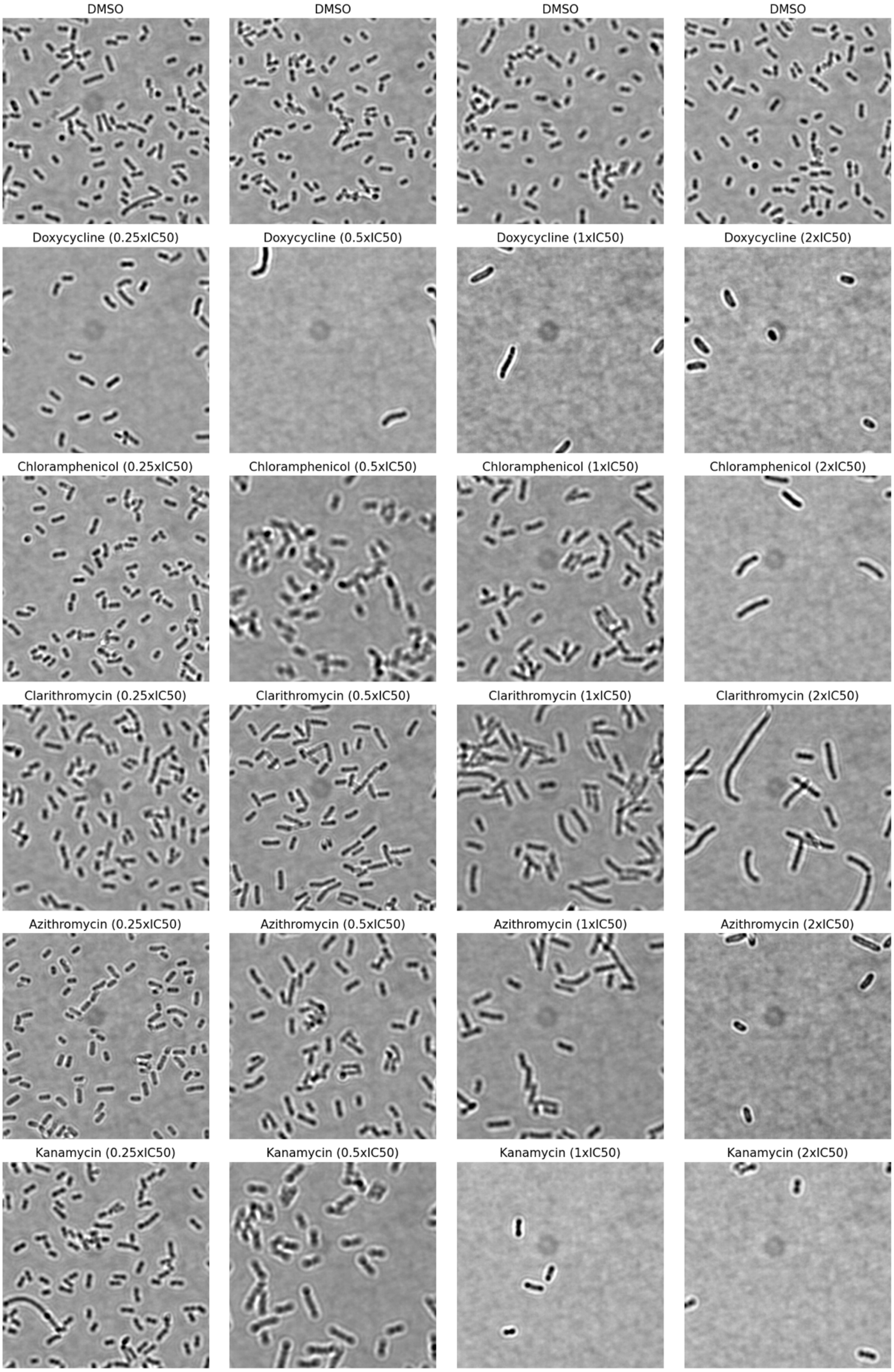

Supplementary Figure 23: Images of *K. pneumoniae* treated with ribosome inhibitors.

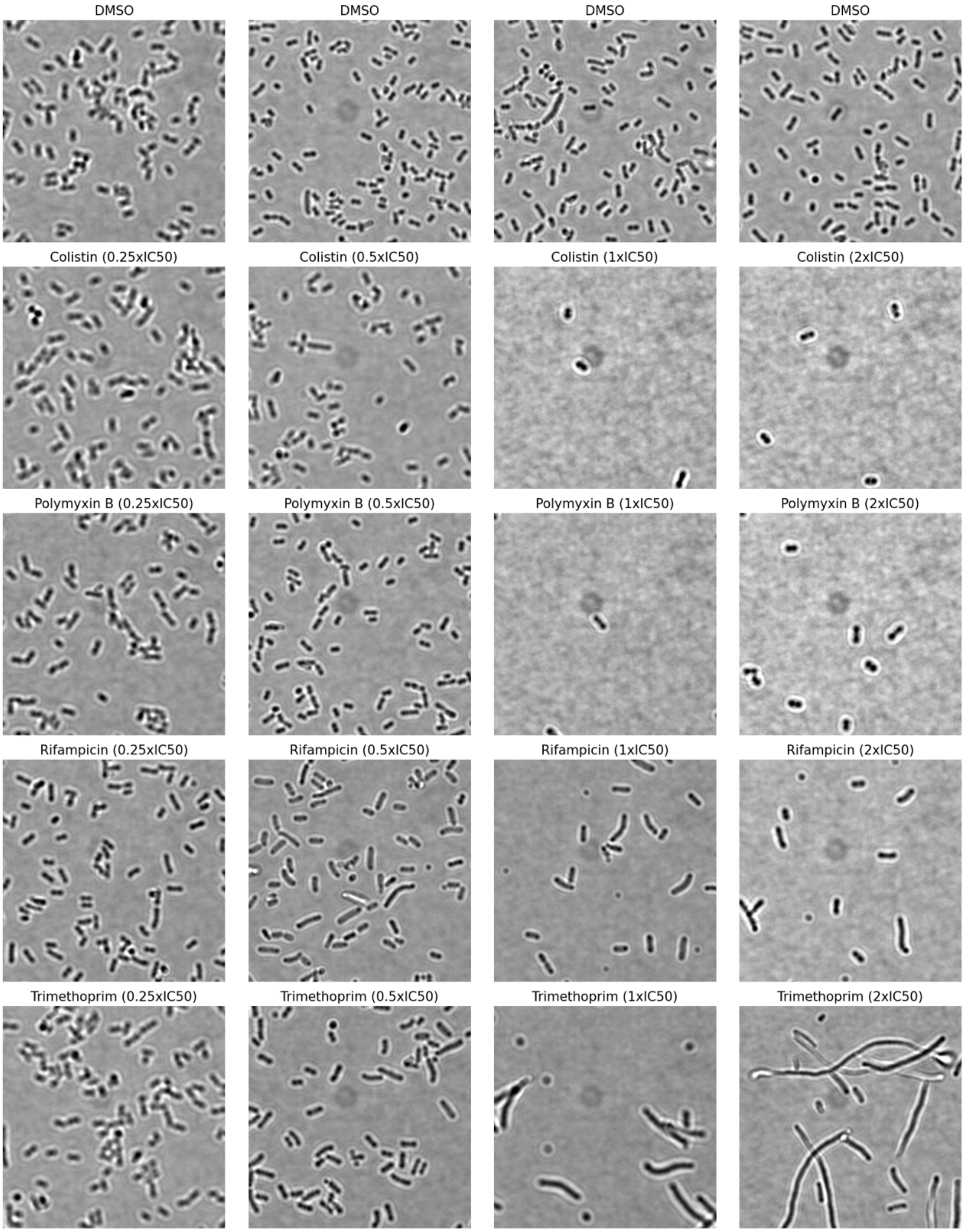

Supplementary Figure 24: Images of *K. pneumoniae* treated with membrane integrity, RNA polymerase and DNA synthesis inhibitors.

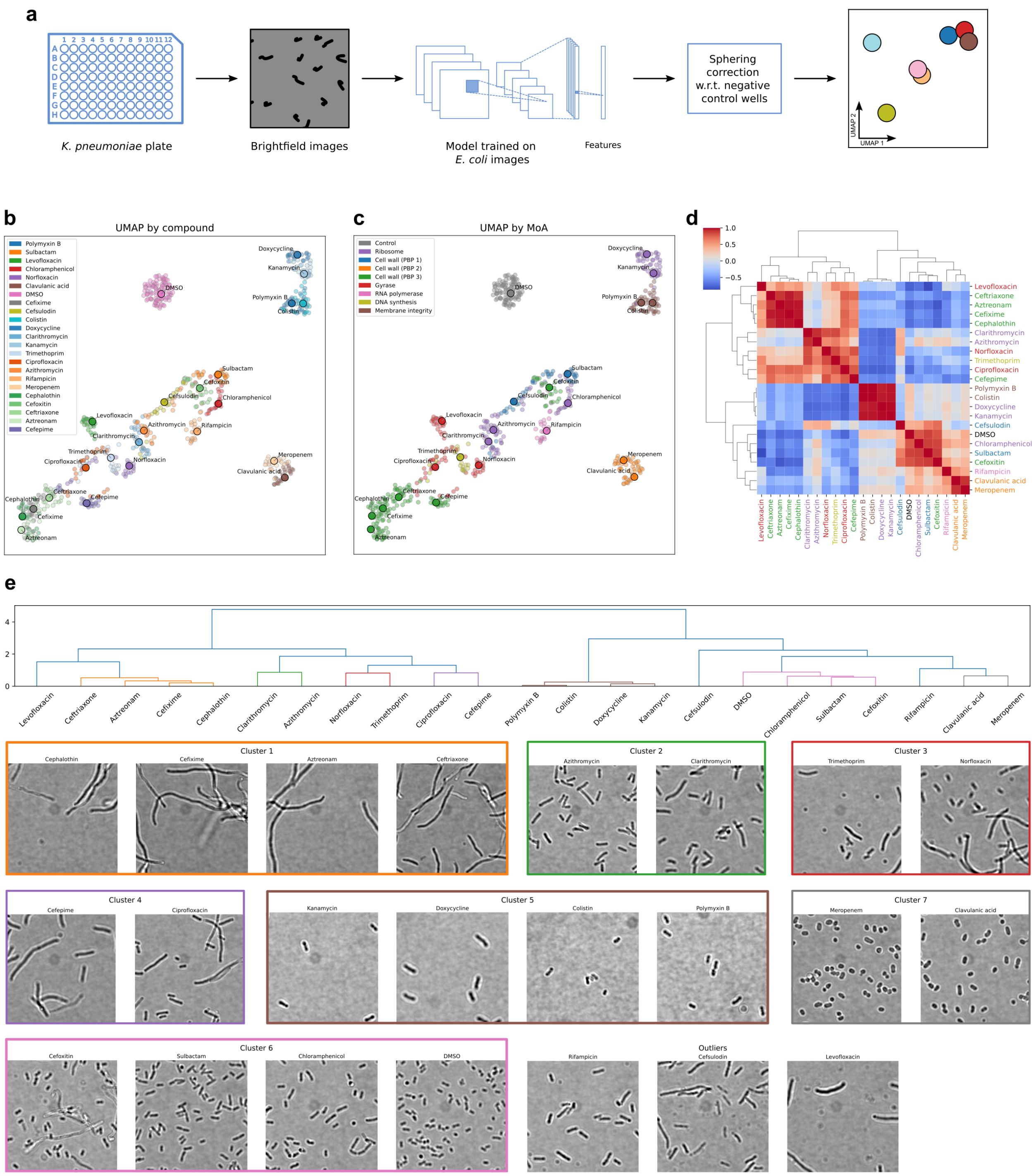

Supplementary Figure 25: Clustering images of drug-treated *K. pneumoniae* using features learned on *E. coli*.

(a) Feature vectors of brightfield images of *K. pneumoniae* bacteria exposed to 1xIC$_{50}$ were obtained with a model trained on brightfield images of *E. coli* bacteria. A sphering correction was computed with respect to the negative controls (DMSO) and applied to feature vectors corresponding to drug conditions to reduce technical variations ('Methods'). **(b, c)** UMAPs of corrected feature vectors coloured by compound identity **(b)** or mode of action (MoA) **(c)**. (**d**) Cosine similarity matrix between median feature vectors computed for each treatment condition with a dendrogram showing hierarchical clustering of feature vectors using Ward's minimum variance method[1]. **(e)** Clusters of compounds were then obtained by setting the threshold to 1 and randomly selected image crops of 32.25 × 32.25 μm are displayed for each condition. Box colours correspond to the clusters in the dendrogram. Source data are provided as a Source Data file.

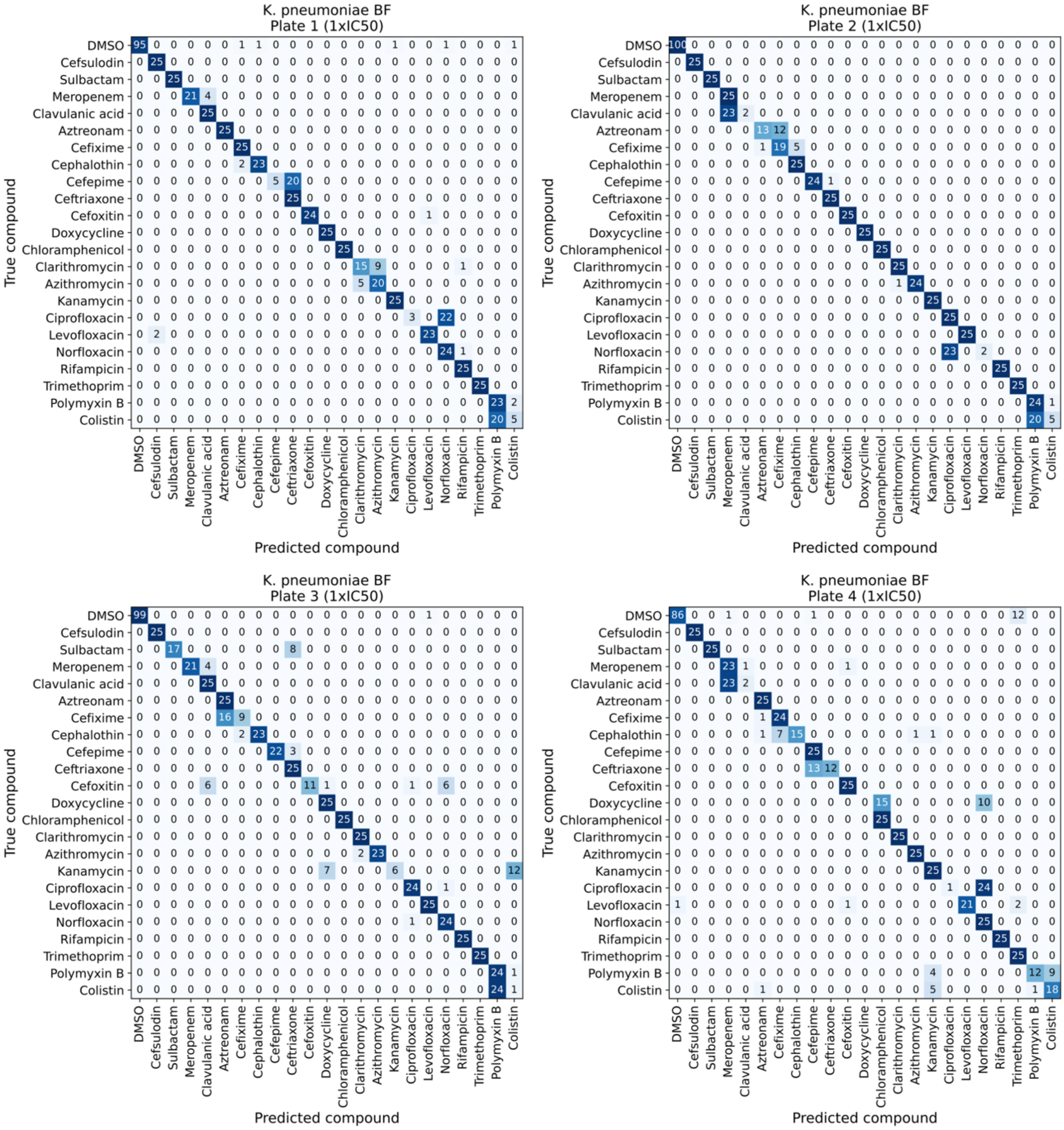

Supplementary Figure 26: Confusion matrices for drug recognition in *K. pneumoniae*.
These 23×23 confusion matrices show the predicted compound identity compared to the ground-truth for CNNs trained on three plates of *K. pneumoniae* images and tested on treatments at the same relative concentration (IC_{50}) in the fourth (hold-out) plate, for each FoV. All plates are biological replicates generated from a parent culture. FoV-level drug classification accuracies for the four test plates are as follows: **(a)** Plate 1: 85.54%. **(b)** Plate 2: 86.62%. **(c)** Plate 3: 85.23%. **(d)** Plate 4: 79.08%. Well-level accuracies are 20/23=86.96% for plates 1-3 and 19/23=82.61% for plate 4: Source data are provided as a Source Data file.

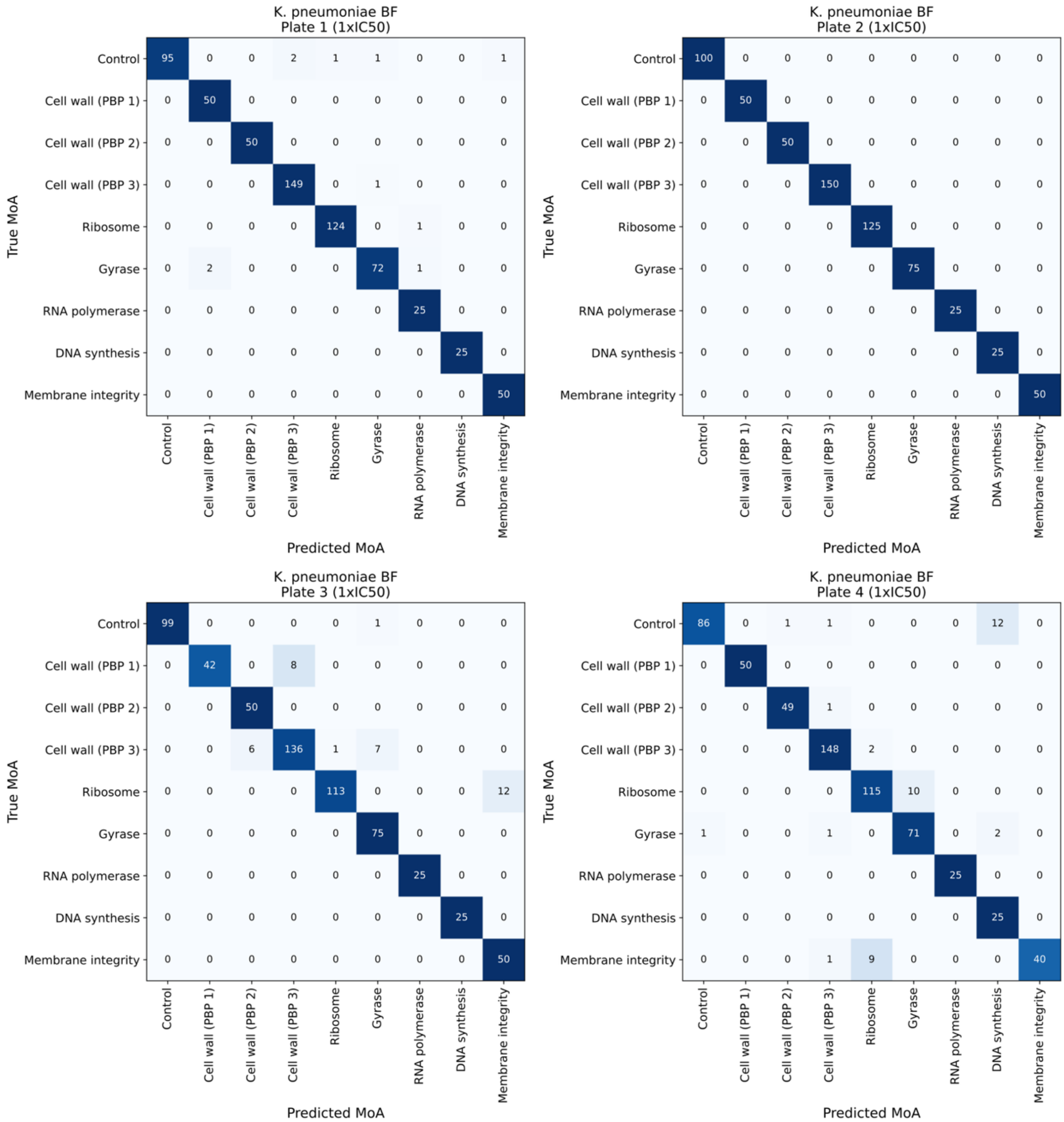

Supplementary Figure 27: Confusion matrices for MoA recognition in *K. pneumoniae*.
These 9×9 confusion matrices show the predicted mode of action (MoA) compared to the ground-truth MoA per field of view (FoV) for CNNs trained on three plates of *K. pneumoniae* images and tested on treatments at the same relative concentration (1xIC_{50}) in the fourth (hold-out) plate. All plates are biological replicates generated from a parent culture. Per-FoV MoA classification accuracies for the four test plates are as follows: **(a)** Plate 1: 98.46%. **(b)** Plate 2: 100%. **(c)** Plate 3: 94.62%. **(d)** Plate 4: 93.69%. Per-well accuracies are 100% for plates 1, 2 and 4 and 22/23 = 95.7% for plate 3. Source data are provided as a Source Data file.

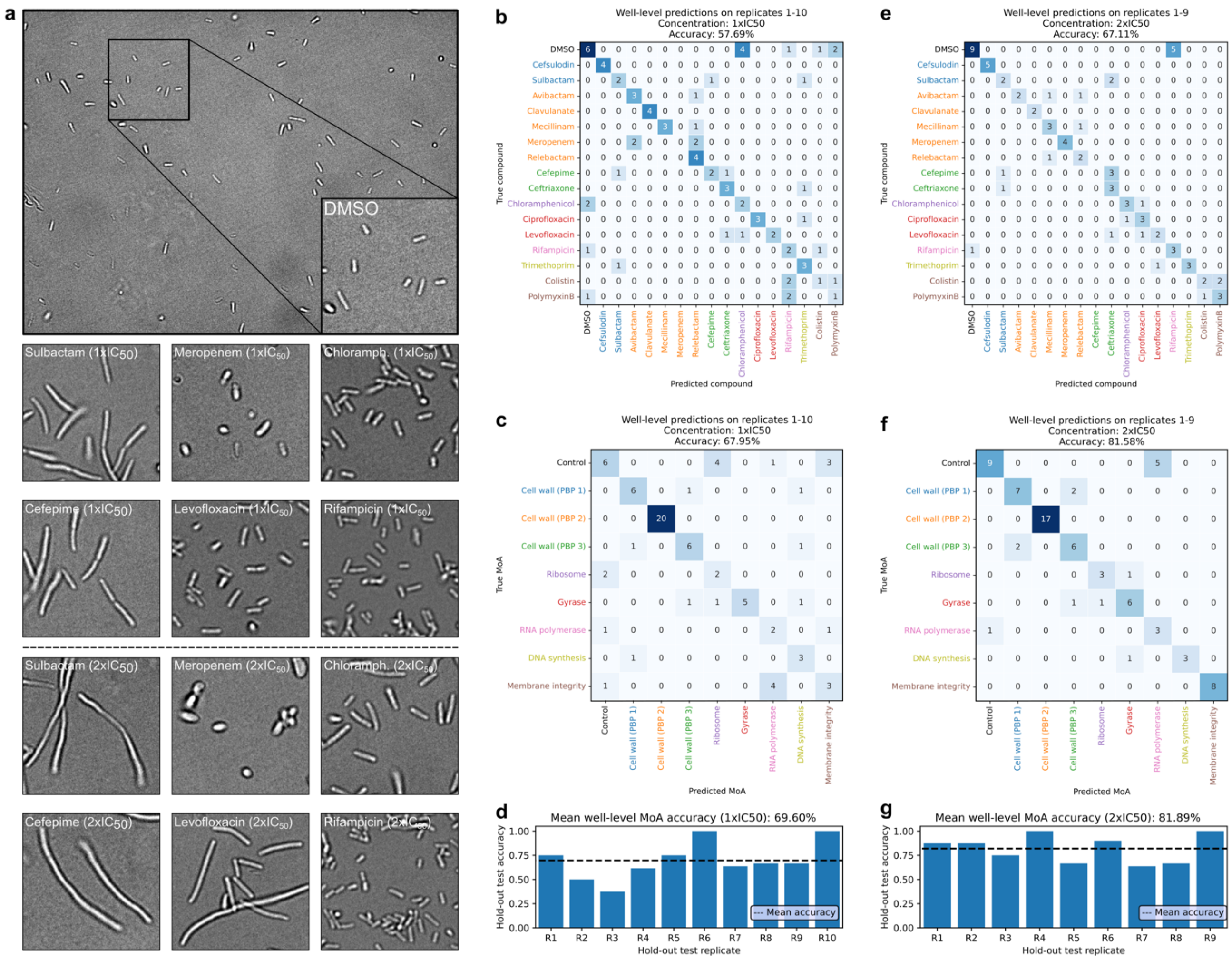

Supplementary Figure 28: Robustness to non-automated imaging of *E. coli*

(a) Images of *E. coli* obtained with a manually operated microscope and treated with manually administered reference antibiotics at $1\times IC_{50}$ or $2\times IC_{50}$, as indicated (or negative control, DMSO). **(b)** Confusion matrix shows well-level compound classification performance based on hold-out test images of *E. coli* treated at $1\times IC_{50}$ concentration aggregated from 10 independently trained CNNs. The CNNs were trained on images of *E. coli* treated at concentrations of $0.25\times IC_{50}$, $0.5\times IC_{50}$, $1\times IC_{50}$ and $2\times IC_{50}$ from rotations of 9 distinct training plates (biological replicates). The number of wells on individual biological replicates ranged from 6 to 58 with 50 FoVs per well (replicate R1: 50 wells; R2: 50 wells; R3: 49 wells; R4: 57 wells; R5: 58 wells; R6: 34 wells; R7: 42 wells; R8: 9 wells; R9: 11 wells; R10: 6 wells). Accuracy (unbalanced): 57.69%. **(c)** Confusion matrix for MoA classification at $1\times IC_{50}$ test concentration. Accuracy (unbalanced): 67.95%. **(d)** MoA classification accuracy (unbalanced) for each hold-out test plate (biological replicates, R1-10), with the remaining nine plates used to train the model. (**e-g**) Same as **b-d** but for test images of *E. coli* treated at $2\times IC_{50}$. The well-level compound classification accuracy (unbalanced) is 67.11% **(e)**, the well-level MoA classification accuracy (unbalanced) is 81.58% **(f)**. Note that in **g** the well-level MoA accuracy is only reported for replicates R1-9 because replicate R10 did not contain wells corresponding to $2\times IC_{50}$. Source data are provided as a Source Data file.

Supplementary Table 1: PBP specificities from literature.

Antibiotic	PBP specificity	PBP1A IC_{50}	PBP1B IC_{50}	PBP2 IC_{50}	PBP3 IC_{50}	Unit	*E. coli* Strain	Ref.
Cefsulodin	1	0.47	3.7	>250	>250	µg/mL	DC0	[2]
Penicillin G	1	0.5	3	0.8	0.9	µg/mL	DC0	[2]
Sulbactam	1	32	1024	128	512	µg/mL	J62-1	[3]
Avibactam	2	$>10^4$	310 ± 460	<100	$>10^4$	µM	MG1655	[4]
Mecillinam	2	>250	>250	<0.25	>250	µg/mL	DC0	[2]
Meropenem	2	$>10^4$	110 ± 86	0.01 ± 0.00	0.2 ± 0.2	µM	MG1655	[4]
Clavulanate	2	128	256	8	256	µg/mL	J62-1	[3]
Relebactam	-	-	-	-	-	-	-	-
Aztreonam	3	970 ± 940	1.6 ± 0.9	$>10^4$	0.09 ± 0.09	µM	MG1655	[4]
Cefepime	3	5.5 ± 2.6	2.6 ± 2.4	0.2 ± 0.1	$<10^{-4}$	µM	MG1655	[4]
Ceftriaxone	3	4.9 ± 2.2	0.2 ± 0.2	1.5 ± 0.9	0.001 ± 0.003	µM	MG1655	[4]

Supplementary Table 2: Antibiotic source

Antibiotic	Product name	Product supplier	Catalog number
Cefsulodin	Cefsulodin Sodium Salt	MP Biochemicals	098677
Penicillin G	Penicillin G sodium salt	Sigma	13752
Sulbactam	Sulbactam	Sigma	PHR2576
Avibactam	Avibactam sodium	RayBiotech	332-11720-1
Mecillinam	Mecillinam	Cayman Chemicals	9002008
Meropenem	Meropenem trihydrate	Sigma	PHR1772
Clavulanate	Lithium clavulanate	Sigma	L0720000
Relebactam	Relebactam	MedChemExpress	HY-16752
Aztreonam	Aztreonam	MP Biomedicals	150415
Cefepime	Cefepime hydrochloride	Sigma	PHR1763
Ceftriaxone	Ceftriaxone disodium salt hemi(heptahydrate)	Sigma	C5793
Chloramphenicol	Chloramphenicol	Sigma	C0378
Clarithromycin	Clarithromycin	Sigma	PHR1038
Doxycycline	Doxycycline hyclate	Sigma	D9891
Kanamycin	Kanamycin sulfate from Streptomyces kanamyceticus	Sigma	K4000
Ciprofloxacin	Ciprofloxacin hydrochloride	Euromedex	UC5074-B
Levofloxacin	Levofloxacin Anhydrous	Sigma	28266
Norfloxacin	Norfloxacin	Sigma	N9890
Rifampicin	Rifampicin	Sigma	R3501
Trimethoprim	Trimethoprim	Sigma	T7883
Colistin	Colistin Sulfate	Sigma	PHR1605
Polymyxin B	Polymyxin B sulfate salt	Sigma	P4932

Supplementary Table 3: Data preprocessing pipeline.

Operation	Function	Description	Applied at test time
Random vertical flip	torchvision.transforms.RandomVerticalFlip	With a 50% probability the image is flipped upside-down	No
Random horizontal flip	torchvision.transforms.RandomHorizontalFlip	With a 50% probability the image is mirrored left-to-right	No
Random rotation	torchvision.transforms.RandomRotation	The image is rotated by a random angle drawn uniformly from the interval [−90°, 90°].	No
Colour jitter	torchvision.transforms.ColorJitter	Random perturbations are applied independently to brightness (±0.15 of the original intensity), contrast (±0.15 of the original contrast), saturation (±0.15 of the original saturation) and hue (±0.1 in the normalised hue space).	No
Centre crop	torchvision.transforms.CenterCrop	The image is cropped to a central square region of 1,528x1,528 pixels to remove edge artifacts.	Yes[#]
Random crop	torchvision.transforms.RandomCrop	A square patch of 1,500x1,500 pixels is sampled at a random location from the previously cropped image adding additional spatial variability.	No
Image tiling	OverlappingCropMultiChannel *	The previously cropped image is further subdivided into nine non-overlapping tiles of 500x500 pixels.	Yes
Conversion to tensor		Tiles are stacked along a new dimension to produce a tensor of shape *(N, C, H, W)* where *N* is the number of tiles, *C* is the number of color channels, *H* and *W* are the image height and width, respectively.	Yes
Resize	torchvision.transforms.Resize	Each tile is rescaled with bilinear interpolation to a fixed dimension of 256x256 pixels.	Yes
Normalisation	torchvision.transforms.Normalize	Pixel intensities are normalised between [-1, 1].	Yes

*Custom implementation

[#]Square region corresponds to 1,500x1,500 pixels when loading the test dataset

Supplementary Table 4: Hyperparameter settings

Hyperparameter	Choice	Comments
Optimiser	Adam	γ=0.001, β_1=0.9, β_2=0.999, $\varepsilon=10^{-8}$
Regularisation	L2	λ=0.001
Batch size	16	The effective batch-size is 144 (16×9), since each image is further subdivided into 9 tiles.
Max. number of epochs	250	Model weights corresponding to the lowest validation loss are selected (early stopping).

Supplementary Table 5: Experimental conditions in replication experiments

This table compares experimental conditions used for *E. coli* imaging in automated high-throughput experiments at Institut Pasteur in Paris (IP) vs. non-automated replication experiments at the Helmholtz Center for Infection Research in Braunschweig (HZI).

	IP	HZI
Bacterial growth		
Volume of overnight culture	5 mL	20 mL
starting inoculum density:	0.05 OD_{600}	0.04 OD_{600}
Well plates for IC_{50} determination	96-well plate (100 µL)	384-well plate (50 µL)
Preparation of imaging plates		
Administration of antibiotics	Antibiotics were dispensed using an Echo 550 liquid handler before adding 100 µL of bacterial suspension (OD_{600} = 0.05)	Antibiotics were manually diluted. An epMotion 5070 was used to dispense 50 µL antibiotic solution and 50 µL bacterial suspension (OD_{600} = 0.1)
Distribution of treatment conditions on well plates	All antibiotics and concentrations on each plate	Due to technical limitations, not all antibiotics and concentrations fit on one plate (edge wells could not be imaged and were therefore left empty)
Centrifugation before imaging	35xg	50xg
Imaging		
Imaging plates	PhenoPlates (Revvity) with 100 µL	96-well half area (Greiner) with 50 µL
Microscopy system	Opera Phenix Plus High-Content Screening System (automated)	Keyence BZ-X810 microscope (manually operated)
Objective	63×/NA 1.15 water immersion	100×/NA 1.45 oil-immersion
FoV positions	non-overlapping randomly chosen positions in each well were the same on individual plates but varied between plates	non-overlapping randomly chosen positions in each well varied within and between plates
Exposure time per image	80 ms	10 ms

Supplementary Table 6: Comparison of antibiotic IC_{50} values against *E. coli* determined at Institut Pasteur and HZI.

Source data are provided as a Source Data file.

Antibiotic	IC_{50} (µg/mL) IP	IC_{50} (µg/mL) HZI	Fold differences between IP and HZI
Cefsulodin	23.77	13.28	1.8
Sulbactam	15.62	20.46	1.3
Avibactam	19.20	6.525	2.9
Mecillinam	0.3009	0.0560	5.4
Meropenem	0.1085	0.2135	2.0
Clavulanate	17.24	21.97	1.3
Relebactam	64	45.14	1.4
Cefepime	0.007517	0.0103	1.4
Ceftriaxone	0.004	0.0077	1.9
Chloramphenicol	3.079	0.9673	3.2
Ciprofloxacin	0.005723	0.0033	1.7
Levofloxacin	0.01089	0.0074	1.5
Rifampicin	3.358	3.169	1.1
Trimethoprim	0.1335	0.2624	2.0
Colistin	0.6465	1.2758	2.0
Polymyxin B	1.166	1.4765	1.3

Supplementary Note 1: Experimental procedures for replication experiments

In order to test the robustness of our approach to changes in the experimental data acquisition, we made a first attempt at acquiring image of *E. coli* treated with reference antibiotics in a distinct laboratory (at HZI Braunschweig) using manual imaging and drug dispension (instead of automated imaging and drug dispension at Institut Pasteur Paris), in addition to other changes in experimental settings, as detailed below and in Supplementary Table 5.

Bacterial growth protocol

E. coli K-12 MG1655 was grown in 20 mL of cation-adjusted Mueller-Hinton Bouillon 2 (MHBII, Merck Millipore cat. 90922) overnight at 37°C until the stationary phase. The culture was then diluted to an OD_{600} of 0.05 in fresh MHBII and grown at 37°C for 1 h 45 min until mid-exponential phase (OD_{600} 0.5 – 0.7) before further dilution. IC_{50} concentrations were determined via broth serial dilutions in 384-well plates with a starting inoculum OD_{600} of 0.04 in a total volume of 50 µL. Micro dilution plates were incubated at 37°C and 180 rpm (throw 25 mm, INFORS HT Multitron) for 18 h. The final cell density was measured using a Tecan Spark plate reader and plotted in GraphPad Prism to calculate IC_{50} values.

For microscopy, 50 µL of antibiotic solutions were pipetted into 96-well plates before adding 50 µL of bacteria diluted from mid-exponential phase to an OD_{600} of 0.1 (final OD_{600} in the well: 0.05). The plates were incubated at 37°C and 180 rpm (throw 3 mm, INFORS HT Multitron) for 2 h 30 min. Subsequently, the supernatant was removed after centrifugation at 4,000 x *g* for 5 min. 100 µL of fixative (2.8% paraformaldehyde, 0.04% glutaraldehyde in PBS) was added and the plates were incubated at room temperature at 800 rpm (3 mm orbit, Eppendorf ThermoMixer® C) for 30 min. Cells were washed twice with 200 µL of PBS by centrifugation and resuspension. Next, the OD_{600} was determined, and the entire plate was diluted based on the median OD of the negative control wells to a calculated OD_{600} of 0.036 in a final volume of 50 µL and the cells were transferred to 96-well half area plates (Greiner; cat. 675090). Due to technical limitations, it was not possible to view the entire plate under the microscope. Consequently, the outer wells were not filled with cells. The fixed and diluted cells were stored at 4°C until imaging.

Non-automated brightfield imaging

Before imaging, plates stored at 4°C were equilibrated to room temperature for at least 20 min and then centrifuged at 50xg for 10 min to bring floating bacteria to the well bottom. Images were then acquired using a non-automated Keyence BZ-X810 microscope with a 100×/1.45 NA oil-immersion objective. In total, 50 non-overlapping fields of view (FoVs) were acquired at randomly chosen positions covering the entire well area. Due to manual operation, images were acquired at different locations within each well. Brightfield images were acquired with exposure times of 10 ms.

Selection of compounds for replication experiments

We restricted our analyses to 16 out of the 22 compounds for which dose-response curves allowed accurate determination of IC_{50}'s.

Supplementary References

1. Ward Jr., J. H. Hierarchical Grouping to Optimize an Objective Function. *Journal of the American Statistical Association* **58**, 236–244 (1963).
2. Curtis, N. A., Orr, D., Ross, G. W. & Boulton, M. G. Affinities of penicillins and cephalosporins for the penicillin-binding proteins of Escherichia coli K-12 and their antibacterial activity. *Antimicrob Agents Chemother* **16**, 533–539 (1979).
3. Moosdeen, F., Williams, J. D. & Yamabe, S. Antibacterial characteristics of YTR 830, a sulfone beta-lactamase inhibitor, compared with those of clavulanic acid and sulbactam. *Antimicrobial Agents and Chemotherapy* **32**, 925–927 (1988).
4. Shirley, J. D., Nauta, K. M. & Carlson, E. E. Live-Cell Profiling of Penicillin-Binding Protein Inhibitors in Escherichia coli MG1655. *ACS Infect. Dis.* **8**, 1241–1252 (2022).
