# Supplementary material for: Deep learning recognises antibiotic modes of action from brightfield images
Source: Nat Commun. 2026 Sep 8;17:10660. doi: 10.1038/s41467-026-76355-0 (PMC13655182; doi:10.1038/s41467-026-76355-0)
Supplement: Supplementary file 2 [file 41467_2026_76355_MOESM2_ESM.pdf]

nature portfolio

Corresponding author(s): Daniel Krentzel, Ivo Boneca, Christophe Zimmer

Last updated by author(s): Jul 2, 2026

Reporting Summary

Nature Portfolio wishes to improve the reproducibility of the work that we publish. This form provides structure for consistency and transparency in reporting. For further information on Nature Portfolio policies, see our Editorial Policies and the Editorial Policy Checklist.

Statistics

For all statistical analyses, confirm that the following items are present in the figure legend, table legend, main text, or Methods section.

n/a	Confirmed	
☐	☒	The exact sample size (*n*) for each experimental group/condition, given as a discrete number and unit of measurement
☒	☐	A statement on whether measurements were taken from distinct samples or whether the same sample was measured repeatedly
☐	☒	The statistical test(s) used AND whether they are one- or two-sided<br>*Only common tests should be described solely by name; describe more complex techniques in the Methods section.*
☒	☐	A description of all covariates tested
☒	☐	A description of any assumptions or corrections, such as tests of normality and adjustment for multiple comparisons
☐	☒	A full description of the statistical parameters including central tendency (e.g. means) or other basic estimates (e.g. regression coefficient) AND variation (e.g. standard deviation) or associated estimates of uncertainty (e.g. confidence intervals)
☐	☒	For null hypothesis testing, the test statistic (e.g. *F*, *t*, *r*) with confidence intervals, effect sizes, degrees of freedom and *P* value noted<br>*Give P values as exact values whenever suitable.*
☒	☐	For Bayesian analysis, information on the choice of priors and Markov chain Monte Carlo settings
☒	☐	For hierarchical and complex designs, identification of the appropriate level for tests and full reporting of outcomes
☒	☐	Estimates of effect sizes (e.g. Cohen's *d*, Pearson's *r*), indicating how they were calculated

Our web collection on statistics for biologists contains articles on many of the points above.

Software and code

Policy information about availability of computer code

Data collection	Datasets were collected on a commercial high-content screening microscope (Revvity Opera Phenix Plus) or a manually operated Keyence microscope and preprocessed using a custom script (https://github.com/krentzd/ai4ab/blob/main/preprocessing/dataset_preprocessing.py)
Data analysis	Data analysis was performed using a combination of custom code and open-source packages. As indicated in the paper: Data analysis was carried out in Python 3.9. The following Python packages were used with version numbers in brackets: 'torch' (1.12.1), 'torchvision' (0.13.1), 'numpy' (1.23.1), 'scipy' (1.9.1), 'pandas' (1.5.0), 'matplotlib' (3.6.0), 'Pillow' (9.2.0), 'scikit-image' (0.19.3), 'imageio' (2.22.0), 'tifffile' (2022.8.12), 'imagecodecs' (2024.1.1), 'scikit-learn' (1.1.2), 'umap-learn' (0.5.3), 'xmltodict' (0.13.0), 'lxml' (4.9.1), 'openpyxl' (3.1.2), 'tensorboard' (2.10.0), and 'huggingface_hub[torch]' (1.7.2). See also our Code Availability statement.

For manuscripts utilizing custom algorithms or software that are central to the research but not yet described in published literature, software must be made available to editors and reviewers. We strongly encourage code deposition in a community repository (e.g. GitHub). See the Nature Portfolio guidelines for submitting code & software for further information.

Data

Policy information about availability of data

All manuscripts must include a data availability statement. This statement should provide the following information, where applicable:

- Accession codes, unique identifiers, or web links for publicly available datasets
- A description of any restrictions on data availability
- For clinical datasets or third party data, please ensure that the statement adheres to our policy

The imaging data generated in this study have been deposited on Bioimage Archive under accession code S-BIAD185139. The latent embeddings and model predictions generated in this study have been deposited on Zenodo40. Source data are provided with this paper.

Research involving human participants, their data, or biological material

Policy information about studies with human participants or human data. See also policy information about sex, gender (identity/presentation), and sexual orientation and race, ethnicity and racism.

Reporting on sex and gender	n/a
Reporting on race, ethnicity, or other socially relevant groupings	n/a
Population characteristics	n/a
Recruitment	n/a
Ethics oversight	n/a

Note that full information on the approval of the study protocol must also be provided in the manuscript.

Field-specific reporting

Please select the one below that is the best fit for your research. If you are not sure, read the appropriate sections before making your selection.

☒ Life sciences ☐ Behavioural & social sciences ☐ Ecological, evolutionary & environmental sciences

For a reference copy of the document with all sections, see nature.com/documents/nr-reporting-summary-flat.pdf

Life sciences study design

All studies must disclose on these points even when the disclosure is negative.

Sample size	A total of 4 96-well plates were acquired both for the E. coli and K. penumoniae experiments. The number of images per well was 50 and 25 respectively. For the replication experiment on E. coli acquired at HZI Braunschweig, a total of 10 plates were acquired.
Data exclusions	Six conditions for the dataset acquired at HZI Braunschweig for the replication experiment were excluded, since the associated dose-response curves did not allow accurate IC50 determination.
Replication	Each experiment was repeated in 4 or more biological replicates.
Randomization	Well-positions on each of the well plates were ranomised to decorrelate spatial locations from perturbations
Blinding	Not relevant, since this is a technique/tool development study

Reporting for specific materials, systems and methods

We require information from authors about some types of materials, experimental systems and methods used in many studies. Here, indicate whether each material, system or method listed is relevant to your study. If you are not sure if a list item applies to your research, read the appropriate section before selecting a response.

Materials & experimental systems

n/a	Involved in the study
☒	☐ Antibodies
☒	☐ Eukaryotic cell lines
☒	☐ Palaeontology and archaeology
☒	☐ Animals and other organisms
☒	☐ Clinical data
☒	☐ Dual use research of concern
☐	☐ Plants

Methods

n/a	Involved in the study
☒	☐ ChIP-seq
☒	☐ Flow cytometry
☒	☐ MRI-based neuroimaging

Plants

Seed stocks	n/a
Novel plant genotypes	n/a
Authentication	n/a
