# Supplementary material for: Deep learning recognises antibiotic modes of action from brightfield images
Source: Nat Commun. 2026 Sep 8;17:10660. doi: 10.1038/s41467-026-76355-0 (PMC13655182; doi:10.1038/s41467-026-76355-0)
Supplement: Supplementary file 3 [file 41467_2026_76355_MOESM3_ESM.pdf]

nature portfolio

Peer Review File

Deep learning recognises antibiotic modes of action from brightfield images

Corresponding Author: Dr Daniel Krentzel

This file contains all reviewer reports in order by version, followed by all author rebuttals in order by version.

Version 0:

Reviewer comments:

Reviewer #1

(Remarks to the Author)
This work describes an effort to develop a label-free, microscopy-based method using deep learning in order to detect antibiotic-induced phenotypes at subinhibitory concentrations, classify the mode of action (MoA) of antibiotics, and create a methodology to identify compounds with novel antibiotic MoAs.
A convolutional neural network, based on EfficientNet-B0, was trained on brightfield images of E. coli treated with 22 reference antibiotics tested at subinhibitory concentrations. The model was trained to classify 89 treatment conditions (22 drugs × 4 concentrations + 1 control), with some additional efforts to control technical confounders. Interestingly, no fluorescent labeling was required, which has clear implications for simplifying such experimental workflows.
The authors achieved high accuracy in MoA classification at the well level using only BF images, even with a low number of images per condition. The model could detect phenotypic changes at low concentrations, suggesting utility for detection of weakly active compounds. Using a leave-one-compound-out strategy, the model correctly predicted MoAs for unseen drugs with ~75% accuracy. Intriguingly, misclassifications often reflected phenotypic similarities rather than model failure, which is also something I've observed in performing similar work with imaging and phenotype classification. It was interesting that the three-channel accuracy contained redundant information, and that the brightfield channel performed similarly. Detection of novel MoAs was also encouraging, and the experimental work with relebactam supported that the model may be able to overcome inaccurate MoAs. The implications for the field are that this approach enables cost-effective, high-throughput phenotypic screening without fluorescent dyes for early-stage antibiotic discovery by identifying both known and novel MoAs from minimal image data.

The study is of course limited in its bacterial diversity, having focused on E. coli, and it's unclear if MoA models trained in one system will be translatable to other bacteria morphologies.
This study, like other "guilt-by-similarity" approaches to phenotypic profiling suffers from the unknown unknowns of MoA labels, although I believe the approach overall could help with improper metadata/labels, as the images are likely closer to ground truth than reported targets.
The CNN here is essentially black box, with limited discussion on which image features drive classification. In future work, incorporating saliency maps or feature attribution methods would enhance transparency.
While the leave-one-MoA-out analysis is promising, it was only tested on 6 MoAs. Detection performance for PBP3 inhibitors was poor (AUC = 0.40), and the reasons were only partially explored. A future effort to test many more less-characterized compounds would improve novelty detection capabilities.
It might be worth speculating on the generalizability of the model, and how one could assess that. For example, testing it on external datasets or simulating domain shifts (e.g., different lighting, magnification) might strengthen claims of generalizability.

(Remarks on code availability)
The code has a README file and installation instructions. Frankly, I'm not in a position to install and run the code myself, so hopefully one of your other reviewers can do this check.

Reviewer #2

(Remarks to the Author)

The manuscript “Deep learning recognises antibiotic modes of action from brightfield images” presents a timely and innovative application of deep learning for identifying antibiotic Modes of Action (MOA) from label-free images. The potential to extract mechanistic information from a simple and accessible imaging modality is a notable achievement with clear relevance to the field of antibiotic discovery. This approach has several key strengths:

1. Early Mechanistic Insight: Traditional primary screens measure growth inhibition (e.g., optical density), providing a simple yes/no answer. This method can provide immediate classification of a hit's potential Mode of Action (MoA). This is valuable for hit prioritization, allowing researchers to focus on compounds with desired or novel mechanisms early on.
2. It helps quickly identify and discard compounds with known MoAs, tackling the major problem of antibiotic rediscovery. The ability to flag phenotypes that don't match the trained classes is a powerful feature. These ‘outlier’ hits are promising candidates for possessing genuinely new MoAs, which are urgently needed.
3. The fact that it can perform at sub-inhibitory concentrations is a key strength for primary screening. Many antibiotics induce clear morphological changes (e.g., filamentation, swelling) well below the concentration needed to kill the bacteria, providing a wider window for detection.
4. The ability of the pipeline to reliably work with label-free methods, such as brightfield imaging, makes it versatile and easy to apply to wide classes of bacterial targets.

While the approach is compelling, the manuscript would benefit from substantial revision to address concerns regarding methodological clarity, model validation, biological generalizability, and the interpretation of key results. Below are detailed points that the authors are requested to address to strengthen the manuscript.

Major Points for Revision
1. Investigating Model Interpretability: A significant opportunity to strengthen the manuscript lies in investigating the model's decision-making process. For a study linking images to biological phenomena, this is an essential validation step.
The manuscript would be substantially strengthened by an explainability analysis. It is strongly recommended that the authors employ established methods such as Gradient-weighted Class Activation Mapping (Grad-CAM) or saliency maps to visualize the image features driving classification.
This analysis would be critical for two reasons:
a. Biological Discovery: It could reveal the specific morphological phenotypes the model has learned, connecting its predictions back to tangible biology and potentially uncovering novel cellular responses.
b. Artifact Detection: Alternatively, the model might be “cheating” by focusing on experimental artifacts instead of true biological phenomena. For example, it could learn that a certain class of antibiotic has lower solubility and tends to precipitate in the well. This may present subtle variations in the image background. This would make the model appear highly accurate in the lab but useless for predicting the MoA of a new, soluble compound, especially when the protocols are changed.
Therefore, analysis of image features driving the classifications would serve as a crucial control to help rule out the possibility that the model is relying on experimental artifacts (e.g., compound precipitate) rather than genuine cellular changes.

2. Addressing Biological Generalizability and Context
The claims of the study would be significantly broadened by demonstrating the approach's applicability beyond the current experimental scope.
a. To demonstrate broader utility, it would be beneficial to test the pipeline against a more diverse panel of clinically relevant pathogens. This could include a key Gram-positive organism (e.g., S. aureus) and other challenging Gram-negative pathogens (e.g., P. aeruginosa). Such experiments would be a powerful demonstration of the method's utility against the pathogens of greatest concern. Otherwise the conclusions of its utility remain limited to E. coli growing in rich media.
b. To enhance clinical relevance, the study would be strengthened by validating the approach using bacteria grown in minimal media, ensuring the learned phenotypes are robust across different metabolic states. A phenotype observed in rich lab media may not be relevant or even present under more realistic conditions. Bacteria in a host environment often face nutrient limitations. Their metabolic state and physiological responses to antibiotics can differ dramatically in minimal media versus rich media. This limitation however is not unique to this study; several recent papers that propose such DL approaches for antibiotic discovery or susceptibility tests have relied on rich media based phenotyping, which makes the tasks easier but less relevant.
c. It is suggested that the method's application be framed more explicitly as a powerful secondary or parallel screen, acknowledging that a morphology-based screen may not detect all classes of antibacterial compounds. The primary goal of a primary screen is to find any compound with antibacterial activity. This model is trained to recognize specific morphological changes. A compound that kills bacteria without inducing a recognizable visual phenotype would be missed entirely. Therefore, it's better suited as a secondary screen or a parallel screen alongside a traditional growth inhibition assay. The authors should discuss these points in making this decision.

3. Enhancing Methodological Clarity and Reproducibility
The manuscript in its current form lacks sufficient detail for the research community to fully reproduce the work and evaluate the findings. The following clarifications are requested:
a. Training and Data Processing: A more detailed description of the training loop and data augmentation pipeline is needed. It would be helpful to know, for instance, if augmentations were applied with a certain probability.
b. For the “minimal number of images” experiment, clarification on the image selection process is required. It is suggested that the authors elaborate on whether multiple models were trained on different random subsets to ensure the robustness of these results. e.g. how does the minimum required number of 7 images depend on the density and size of cells, and the magnification of the imaging system used here? Providing access to the relevant codes to the community would also be

highly beneficial and critical for the community to test the applicability in wider contexts.
Image Preprocessing Decisions:
c. A justification is requested for the decision to convert 16-bit images to 8-bit, as this step may discard useful data without offering a clear computational advantage. Since the model relies on morphology as well as texture from the BF images, it would be helpful to benchmark the performance across the raw and 8-bit formats.
Similarly, the rationale for downsizing images to 256x256 pixels should be explained. A comparison with models trained on larger tiles or crops would be informative to demonstrate that this loss of resolution does not negatively impact performance. Bacterial cells are small, and especially for poor media conditions or other smaller species, such as the relevant ones suggested below, the number of pixels per cell is a critical factor.

d. ImageNet Pretrained Model Implementation: The manuscript would benefit from clarification on the image normalization strategy used, particularly regarding the discrepancy between the described (-1, 1) scaling and the standard ImageNet-specific mean/std normalization expected by pretrained PyTorch models.
Finally, it is crucial for reproducibility that the authors specify precisely how the single-channel brightfield image was adapted for the three-channel pretrained model architecture.

4. Resolving Inconsistencies and Clarifying Model Behaviour
The manuscript presents several results that require further investigation and explanation to build confidence in the model's robustness and the conclusions drawn.
Hold-out Plate Variability (Supp. Fig. 3): An investigation and discussion of the potential causes for the high variability and specific confusion patterns across hold-out plates are requested.
Concentration-Dependent Accuracy: A deeper analysis is needed to explain why prediction accuracy is highly sensitive to concentration. It is suggested that interpretability tools could be used to show what morphological features are gained at higher concentrations.
Direct MOA Prediction: It would be a valuable benchmark if the authors trained and evaluated a model that directly predicts the MOA class, rather than relying on a post-hoc assignment after antibiotic-level prediction. This would provide a more direct test of the model's ability to learn MOA-specific features.
LOCO vs. Cosine Similarity: A discussion is needed to clarify the apparent discrepancies between the k-NN classification in the LOCO analysis and the cosine similarity of feature vectors.
Model Performance and Implementation Choices: An explanation is requested for why the brightfield-only model achieves the highest per-well accuracy but the lowest per-FOV accuracy. Furthermore, a justification for using k-NN for classification instead of the model's output logits should be provided.

Minor Points & Suggestions:
i. The manuscript could be enhanced by a brief discussion on the potential of a multi-task learning approach (e.g., predicting compound and concentration simultaneously).
ii. To better contextualize the data requirements, it would be informative to see how model performance is affected when data augmentation is turned off.
iii. For the Leave-One-MoA-Out (LOMO) tests, clarification is needed on whether the training data was rebalanced.
iv. A higher-resolution version of Supplementary Figure 3 and a more intuitive color scheme or direct labeling for Figure 2e are requested to improve readability.

(Remarks on code availability)
The code was not linked in the main manuscirpt. We missed it until we accessed this review submission page. It will be helpful to include to the github link for code in the main paper and the requested details in the review should be included in the methods details of the paper.

Reviewer #3

(Remarks to the Author)
I co-reviewed this manuscript with one of the reviewers who provided the listed reports. This is part of the Nature Communications initiative to facilitate training in peer review and to provide appropriate recognition for Early Career Researchers who co-review manuscripts.

(Remarks on code availability)

Version 1:

Reviewer comments:

Reviewer #1

(Remarks to the Author)

(Remarks on code availability)

Reviewer #2

(Remarks to the Author)
The authors have made substantial revisions to the manuscript, and many of the major conceptual and methodological concerns raised in the previous round have been addressed. I appreciate the addition of Grad-CAM analyses and composite-image controls, the new K. pneumoniae experiments, the bit-depth and image-resizing comparisons, the HZI/Braunschweig replication experiments, the expanded description of preprocessing and augmentation, the direct MoA-label training comparison, the analysis of cell-density effects on the training-image requirement, and the correction of the previous inconsistency in the channel-comparison analysis. These additions substantially strengthen the manuscript and improve the transparency of the work.

I also appreciate that the authors now more explicitly discuss limitations, including the restricted species scope, the remaining limitations of Grad-CAM for biological interpretation, the effect of concentration on phenotype strength, and the limitations of discrete MoA labels. Overall, the revised manuscript is considerably stronger than the original submission.

However after checking the linked code, I could not find the code for the Grad-CAM analyses or much of the supplementary analyses, K. pneumoniae, HZI replication or bit-depth/resizing comparisons, and only see notebooks for figures 2 to 6 at the GitHub link. Were these the only codes intended to be shared?

While browsing the code I came across some points that would benefit from some clarification:
During training the validation uses the same training augmentation (get_train_transforms) which will apply random flips, rotations etc. Since checkpoints are selected based on validation loss, the model selection is stochastic. Typically validation should use deterministic test/eval transforms. The authors should justify this choice or demonstrate that it does not affect checkpoint selection or reported performance.

I am confused by the term “well-level”. Can the authors please clarify what they mean? In utils.py, _compute_conditional_max_accuracy() loops over set(cond_labels), not over well identifiers. Am I right that since each compound and dose is one well, grouping by cond_label is effectively grouping over individual wells?

Therefore, if use_moa_labels is set to True, _compute_conditional_moa_max_accuracy() wil be called and this first maps the FOV labels to the MoA labels, and then loops over set(moa_cond_labels). Does this not mean that the MoA majority vote is over all FOVs sharing the same true MoA, and not over each well? I note that the revised manuscript gives a classification accuracy at the well-level for one of the plates of 88.9%, which is 8/9. The authors should clarify the denominator for the reported “well-level” MoA accuracies and, if necessary, recompute true per-well MoA accuracy. I believe the “seven images per treatment condition” result also inherits this issue. The authors should verify whether the “seven images per condition” result is robust when evaluated using true per-well or per-condition MoA predictions.

I am also confused by the scaling of the feature vectors in the LOMO analysis. LOMOPlotter.plot_roc_curves fits a StandardScaler to feat_vecs_ which is the full feature set, is it not? This seems to be done before fitting the local outlier factor model. Surely for novelty detection scaling and other preprocessing should be fitted on the training/reference distribution only, and then applied to the held-out candidate compounds? The authors should check whether this affects the reported ROC values. The same issue appears in plot_novelty_score() and related LOMO visualisation code.

The same is true for plot_roc_curve (singular) for Figure 4. Cosine distance is not translation invariant so centering the data on feat_vecs could influence the scoring space. I do not know if this will change the ROC values at all but the authors should check their implementation because this seems like a potential data leakage issue. The authors should either recompute the ROC curves without this centring step, or fit any preprocessing only on an appropriate reference distribution, such as training-set controls, before applying it to held-out samples.

Two more final points:
The authors might want to consider citing similar work on DL-based novelty detection that was published recently (after their preprint): https://doi.org/10.3389/fmicb.2025.1640252. The authors might want to soften some claims about novelty detection in the introduction.

The Zoffman et al paper in Scientific Reports, cited by the authors defines a concept known as the “lowest observed effective dose” (LOED) and finds that they can detect changes in certain features at an MIC/LOED ratio of 3.9x. They perform analysis at 2x-4x LOED, which is in the sub-MIC range, although I grant they likely don’t go as low as this manuscript. However the authors might want to soften the claims in the introduction.

(Remarks on code availability)
NA

Reviewer #3

(Remarks to the Author)
I co-reviewed this manuscript with one of the reviewers who provided the listed reports. This is part of the Nature Communications initiative to facilitate training in peer review and to provide appropriate recognition for Early Career

Researchers who co-review manuscripts.

(Remarks on code availability)
Please see my comments on the code which are attached to the main reviewer.

Open Access This Peer Review File is licensed under a Creative Commons Attribution 4.0 International License, which permits use, sharing, adaptation, distribution and reproduction in any medium or format, as long as you give appropriate credit to the original author(s) and the source, provide a link to the Creative Commons license, and indicate if changes were made.
In cases where reviewers are anonymous, credit should be given to 'Anonymous Referee' and the source.
The images or other third party material in this Peer Review File are included in the article's Creative Commons license, unless indicated otherwise in a credit line to the material. If material is not included in the article's Creative Commons license and your intended use is not permitted by statutory regulation or exceeds the permitted use, you will need to obtain permission directly from the copyright holder.
To view a copy of this license, visit https://creativecommons.org/licenses/by/4.0/

Krentzel et al. point-by-point response

REVIEWER COMMENTS

We thank all reviewers for their careful and constructive analysis of our paper. Their comments prompted us to thoroughly revise our paper with a substantial number of additional experiments and analyses and to improve the clarity and methodological details provided in our paper. These changes are reflected in part by the addition of two main Figures (Figs. 7 and 8) and 1 main Table (Table 2), 10 Supplementary Figures, 4 new Supplementary Tables and 1 Supplementary Notes, as well as an additional Results section, an expanded Discussion and Methods section.
Below is a detailed point by point response to all comments.

We also took the opportunity to improve other aspects of the paper and made two corrections in Figures 4 and Supp. Fig. 5 (see Table below and response R2N). All changes to the main text have been tracked.

The Tables below are intended to help reviewers track changes in the main Figures and Supplementary items:

**Figure or Table**	**Type of change**	**Changes**
Fig. 1	Minor	we removed 'E. coli' in panel 1 to reflect the inclusion of Klebsiella data
Fig. 2	Minor	We included annotations to the UMAP in panel e
Fig. 3	Unchanged	
Fig. 4	Correction	Panel e was corrected (in the submitted version this plot corresponded to a different test plate, leading to an inconsistency with the corresponding text, which is now resolved)
Fig. 5	Unchanged	
Fig. 6	Unchanged	
Fig. 7	**New Figure**	See response R1A
Fig. 8	**New Figure**	See response R2D
Table 1	Unchanged	
Table 2	**New Table**	Antibiotics used for K. pneumoniae

**Supplementary Figure in revised paper**	**Corresponding Supplementary Figure in initial ms.**	**Changes in Figure (not including minor changes in the legend)**
Supp. Fig. 1	Supp. Fig. 1	Unchanged
Supp. Fig. 2a-f	Supp. Fig. 2a-f	Unchanged

Supp. Fig. 3	Supp. Fig. 3	Improved resolution, ordering compounds by MoA, addition of visual guides
Supp. Fig. 4	Supp. Fig. 4	Unchanged
Supp. Fig. 5	Supp. Fig. 5	Corrected – see response R2N
Supp. Fig. 6	Supp. Fig. 6	Unchanged
Supp. Fig. 7		**New Figure**
Supp. Fig. 8		**New Figure**
Supp. Fig. 9		**New Figure**
Supp. Fig. 10		**New Figure**
Supp. Fig. 11	Supp. Fig. 7	Unchanged
Supp. Fig. 12	Supp. Fig. 8	Unchanged
Supp. Fig. 13		**New Figure**
Supp. Fig. 14a-f		**New Figure**
Supp. Fig. 15		**New Figure**
Supp. Fig. 16		**New Figure**
Supp. Fig. 17		**New Figure**
Supp. Fig. 18		**New Figure**
Supp. Table 1	Supp. Table 1	Unchanged
Supp. Table 2	Supp. Table 2	Unchanged
Supp. Table 3		**New Table**
Supp. Table 4		**New Table**
Supp. Table 5		**New Table**
Supp. Table 6		**New Table**
Supp. Note 1		**New Note**

Reviewer #1 (Remarks to the Author):

This work describes an effort to develop a label-free, microscopy-based method using deep learning in order to detect antibiotic-induced phenotypes at subinhibitory concentrations, classify the mode of action (MoA) of antibiotics, and create a methodology to identify compounds with novel antibiotic MoAs.

A convolutional neural network, based on EfficientNet-B0, was trained on brightfield images of E. coli treated with 22 reference antibiotics tested at subinhibitory concentrations. The model was trained to classify 89 treatment conditions (22 drugs × 4 concentrations + 1 control), with some additional efforts to control technical confounders. Interestingly, no fluorescent labeling was required, which has clear implications for simplifying such experimental workflows.
The authors achieved high accuracy in MoA classification at the well level using only BF images, even with a low number of images per condition. The model could detect phenotypic changes at low concentrations, suggesting utility for detection of weakly active compounds. Using a leave-one-compound-out strategy, the model correctly predicted MoAs for unseen drugs with ~75% accuracy. Intriguingly, misclassifications often reflected phenotypic similarities rather than model failure, which is also something I’ve observed in performing similar work with imaging and phenotype classification. It was interesting that the three-channel accuracy

contained redundant information, and that the brightfield channel performed similarly. Detection of novel MoAs was also encouraging, and the experimental work with relebactam supported that the model may be able to overcome inaccurate MoAs. The implications for the field are that this approach enables cost-effective, high-throughput phenotypic screening without fluorescent dyes for early-stage antibiotic discovery by identifying both known and novel MoAs from minimal image data.

We thank the reviewer for this summary and appreciation of the impact of our work in the field of high-throughput phenotypic screening for early-stage antibiotic discovery.

The study is of course limited in its bacterial diversity, having focused on E. coli, and it's unclear if MoA models trained in one system will be translatable to other bacteria morphologies.

R1A:
We agree that the restriction to *E. coli* was a main limitation of our study, as we had mentioned in the Discussion section. Although we consider an exhaustive analysis of other bacterial species as to be outside the scope of our paper, the reviewer's comments prompted us to repeat our experiments and analysis on *Klebsiella pneumoniae*, another Gram negative bacterium of high concern.

In brief, we exposed *K. pneumoniae* to 22 drugs including 18 identical to those used in *E. coli* (Penicillin G, Avibactam, Mecillinam, and Relebactam were replaced by Cefixime, Cephalothin, Cefoxitin, and Azithromycin). We used these antibiotics at four different concentrations, ranging from 0.25xIC50 to 2xIC50.

At first, we attempted to directly use the model trained on *E. coli* cells to predict the compounds (for the 18 compounds common to both data sets) or MoA from *K. pneumoniae* cells. However this led to poor accuracy.

As a second step we used the *E. coli* model to extract feature vectors and cluster treatment conditions in *K. pneumoniae*, as shown in our **new Supplementary Figure 15.** This allowed some meaningful grouping of compounds, suggesting that some features learned on *E. coli* transfer to *K. pneumoniae,* but also some incorrect groupings.

We therefore repeated the approach used for *E. coli*, i.e. we retrained our model on three biological replicates (plates), and tested it on the hold out plate. We focused our evaluations on the concentration 1xIC50 and computed drug-classification and MoA-classification accuracies on the level of individual FoV and wells. We are happy to report that classification results are excellent: drug recognition accuracies are similarly high as for *E. coli*, and we achieve perfect MoA classification on a per-well basis in all four replicates, as shown in our new **Figure 7**. These new results indicate that our method is not restricted to *E. coli* but extends to other species.

- ⇨ We added a new Results section entitled "MoA recognition from brightfield images in Klebsiella" along with **a new Figure 7** and **new Supplementary Figures 14-17**

⇨ We also updated our Discussion by adding the following sentence:

"While we focused on E. coli, our analysis of K. pneumoniae data also supports excellent recognition of drug MoA in a second bacterial species of high relevance to antimicrobial resistance (Fig. 7)."

Of course, these results on *K. pneumoniae* do not demonstrate applicability to other species in general. In particular, since *E. coli* and *K. pneumoniae* are both Gram negative, one may ask whether the approach extends to Gram positive bacteria. However, we point the reviewer to a recently submitted preprint that successfully extends our deep learning approach (with some changes to the training strategy and use of fluorescent channels) to *C. glutamicum,* a Gram positive bacterium, which has a much smaller size and displays much less pronounced morphological changes upon drug treatment than *E. coli.* :

⇨ We added the following sentences to the Discussion section:

"The results in our study are limited to E. coli and K. pneumoniae bacteria, two Gram negative bacteria with pronounced morphological changes in response to drug exposure. To what extent our BF-only methodology extends to Gram positive bacteria and/or other Gram negative species with more subtle morphological phenotypes remains to be determined. However, we point out that a variation of our deep learning based analysis method proved effective at analysing images of the Gram-positive bacterium Corynebacterium glutamicum, a proxy for Mycobacterium tuberculosis, despite its significantly smaller size and less pronounced phenotypes, as detailed in Krentzel et al29. Follow-up work could investigate the applicability of our approach to a broader range of bacterial species."

This study, like other "guilt-by-similarity" approaches to phenotypic profiling suffers from the unknown unknowns of MoA labels, although I believe the approach overall could help with improper metadata/labels, as the images are likely closer to ground truth than reported targets.

R1B:

We agree that approaches that assign MoAs using phenotypic similarity based on MoA labels are limited by the caveats associated to these labels. An example of this in our paper is provided by Penicillin G and Sulbactam, which according to the literature specifically target PBP1A or PBP1B, whereas our affinity assays show promiscuous targeting of several other PBPs (Supp. Fig 12). But as the reviewer pointed out, our phenotypic profiling can help flag such discrepancies, as indeed illustrated by our discordant MoA predictions for these three drugs. We also stress that our CNN model itself is not predicated on MoA annotations, since it is only trained on drug and concentration labels, as opposed to MoA.

⇨ To better highlight these aspects we updated the following passage in the Discussion (underlined is new):

Third, we emphasise that our model was trained solely to predict the combinations of drugs and concentrations and thus requires no explicit prior knowledge of the MoA. Despite this, we show that our model implicitly encodes similarities by MoA in its latent space. This is a welcome feature, as it means that a straightforward supervised learning strategy with well-defined and unambiguous labels (drugs and concentrations) can learn biologically meaningful representations from the image data alone in a manner immune to incorrect MoA annotations. On the contrary, our approach can be used to flag potential annotation errors.

⇨ and added the following sentence to the Discussion:

"In any case, the discrepancies between the predicted MoAs of compounds that nominally share the same MoA (such as cefsulodin, sulbactam and penicillin G in Fig. 5h) suggest that MoA annotations should be revisited. Corrections of MoA ground-truth assignments e.g. by affinity assays are then likely to yield improved image-based prediction accuracies."

The CNN here is essentially black box, with limited discussion on which image features drive classification. In future work, incorporating saliency maps or feature attribution methods would enhance transparency.

R1C:
We agree with the reviewer that our CNN is essentially a black box and does not in itself provide insights into which image features drive classification. This comment prompted us to explore the use of saliency maps, which we computed using the popular Gradient-weighted Class Activation Mapping (Grad-CAM) method (Selvaraju et al. ICCV 2017). Our new Grad-CAM analysis highlights bacterial cell features as opposed to the background, as shown in **our new Supplementary Figure 7.** Moreover, in a 'counterfactual analysis' using composite images of bacteria treated with distinct antibiotics, the saliency maps exhibit higher activations in the image halves corresponding to the antibiotic that matches the chosen Grad-CAM class (**Supplementary Figure 8**). These results are consistent with our CNNs having learned image features specific to each MoA as opposed to confounding factors such as variations in background intensity.

⇨ We included these new analyses as **Supplementary Figures 7 and 8** and mention them as follows at the end of the Results section 'Brightfield images alone allow accurate recognition of drug MoA':

"To shed light on the image features used by our model, we computed saliency maps using Gradient-weighted Class Activation Mapping (Grad-CAM)16, which highlights the parts of the image most important for the prediction of each class. Grad-CAM highlighted different features of bacterial cells, rather than the background (Supplementary Fig. 7, Methods), consistent with the CNN having learned biologically relevant image features as opposed to technical confounders. In composite images of cells treated with antibiotics of distinct MoAs, the class-dependent activations are consistently stronger for the image halves corresponding to the antibiotic class, as expected if the CNN has learned MoA-specific features (Supplementary Fig. 8)."

⇨ We provide details on the calculations of Grad-CAM in a new Methods subsection 'Grad-CAM saliency maps'.

Nevertheless, while these Grad-CAM analyses are comforting, we consider that they still fall short of fully and clearly revealing the detailed specific image features, eg cellular morphologies, that drive CNN classifications for different MoA. Indeed, established tools for explainable AI such as Grad-CAM still face important limitations, as highlighted by prior studies, eg Kamakshi & Krishnan, AI 2023, Taimeshkanov, Sicre and Garreau ECML PKDD 2024. As the field of X-AI matures, more powerful methods will hopefully allow to extract more detailed and discriminative image features from the data.

⇨ We added a few sentences to mention this perspective at the end of the Discussion:

"Finally, we acknowledge that despite our use of Grad-CAM saliency maps (Supplementary Figs. 7, 8), our approach provides limited insights into the MoA-specific image features learned by the CNN. Leveraging recent advances in

explainable AI to overcome limitations of Grad-CAM or similar methods and extract more detailed information about these features provides another exciting perspective for follow-up studies30–32."

While the leave-one-MoA-out analysis is promising, it was only tested on 6 MoAs. Detection performance for PBP3 inhibitors was poor (AUC = 0.40), and the reasons were only partially explored. A future effort to test many more less-characterized compounds would improve novelty detection capabilities.

R1D:
Our leave-one-MoA-out analysis was restricted to only 6 MoA out of the 8 MoA spanned by our reference antibiotics (see Table 1) because we left out the two MoAs represented by only a single antibiotic, namely RNA polymerase (rifampicin) and DNA synthesis (trimethoprim). The reason is slightly technical: to evaluate the ability to detect a truly novel MoA and compute ROC curves (Fig. 6e) we needed negative controls in the form of unseen compounds from a seen MoA. Thus in this analysis we needed MoAs with at least two compounds, one of which could be used in the training set (when assessing MoA detection for another left-out MoA) while the other was used as a negative control during testing.

As for the poor detection performance for PBP3 inhibitors (Fig. 6e): this appears to be a consequence of the surprisingly subtle phenotypes induced by exposing our bacteria to these compounds at concentrations up to 1xIC50. Although our CNN is able to detect and recognize this phenotype when directly trained to recognize PBP3 inhibitors (**Fig. 3e**), the phenotype seems to be too weak to be detected as distinct from control in the absence of any PBP3 inhibitor in the training data.
We have performed new imaging experiments using higher concentrations of the PBP3 inhibitors, namely 1xIC50, 2xIC50, 4xIC50 and 8xIC50. These images revealed a clear change in phenotype between 2xIC50 and 4xIC50, as shown in our new **Suppl. Fig. 13.** Consistently, the performance of MoA-novelty detection increased (from a AUC below 0.5 to 0.87); see new **Suppl. Fig. 13)**.

⇨ We have added these new data in a new **Supplementary Figure 13** and added the following sentence to the Results section 'Detecting drugs with unseen modes of action'**:**

"However, this was dependent on concentration, since at 4×IC50 or higher PBP3 inhibitors led to a clear change in phenotype enabling good novelty detection (AUC=0.87) (Supplementary Fig. 13)."

It might be worth speculating on the generalizability of the model, and how one could assess that. For example, testing it on external datasets or simulating domain shifts (e.g., different lighting, magnification) might strengthen claims of generalizability.

R1E: We appreciate this comment, which we addressed in two ways.

First, we analyzed the robustness of our model to differences in magnification by comparing performance with or without image down-sampling, which indicated robustness to a two-fold change in image resolution. See **our new Supplementary Fig. 9** and details under R2G below.

⇨ We included a new **Supplementary Figure 9** showing the robustness to two-fold resolution change, and added a sentence in the Results section (see R2G below)

Second, the comment prompted us to repeat some of our *E. coli* experiments in a different lab, namely the department of Mark Brönstrup (HZI Braunschweig) - all other experiments were performed at the Institut Pasteur in Paris (unit of Ivo Boneca).

In the new experiments in Braunschweig, IC50s were determined independently (leading to some differences with the IC50s determined in Paris). Moreover, images were obtained using a manually operated microscope with a 100x oil immersion objective (as opposed to the automated high-throughput imaging system used with a 63x water immersion objective in Paris). Additionally, compounds in the Braunschweig experiments were administered manually, based on independently determined IC50 values, whereas the compounds in Paris were distributed automatically with an automated liquid handling system. These and other important differences are listed in **our new Supplementary Tables 5,6** along with **Supplementary Note 1**, which describes the experiments performed in Braunschweig.
Please note that although we initially considered the same 22 compounds as in the Paris experiments, we restricted our analyses to 16 out of 22 drugs for which dose-response curves allowed accurate determination of IC50.
We retrained and evaluated the model on these new images, obtained at concentrations of 0.25x to 2xIC50, using held-out test plates for evaluation as before. For testing we focused on the treatments at 2xIC50. As shown in **our new Supplementary Figure 18**, the unbalanced drug recognition accuracy across the 10 plates was 71.2% and the MoA recognition accuracy 85.0%. Although these accuracies are not quite as good as those we reported based on the Paris *E. coli* data, they confirm that MoA recognition from BF images is possible using different experimental setups and indicate a promising level of robustness. Furthermore, these data suggest that our method may be applicable to settings without automation of imaging or drug dispensing, extending its potential reach to many more biological labs, including low-resource environments. A careful analysis of the differences between the data sets might allow to identify the causes of the remaining classification errors, but we leave this for future work.

⇨ We included a new **Supplementary Figure 18** with the analysis of the new data from Braunschweig along with a new **Supplementary Note 1** and **Supplementary Table 5** about the experimental procedures and a new **Supplementary Table 6** about IC50 values.

⇨ We added the following paragraph to the Discussion:

"At the technical level, it will be interesting to determine the robustness of our method to changes in experimental conditions such as microscopy settings. We verified that MoA recognition accuracy is robust to changes in the image bit-depth (16-bit vs. 8-bit) and to a roughly 2-fold change in image resolution (500x500 vs 256x256 pixels)

(Supplementary Fig. 9). Additionally, we acquired BF images of unstained E. coli in a different laboratory with a conventional, manually operated imaging system. Compounds were administered manually at concentrations ranging from 0.25× to 2×IC50, based on independently determined IC50 values, and many other experimental conditions differed (Supplementary Note 1, Supplementary Tables 5,6). After retraining our CNN on this new data set, we achieved a mean MoA recognition accuracy per well on hold-out test plates of 59.3% at 1×IC50 and 82.3% at 2×IC50 (Supplementary Fig. 18). Despite a drop in performance, these data suggest that our approach is relatively robust to changes in experimental conditions and may be applied to characterize compound MoA even without automated microscopy and liquid handling systems. This could considerably extend the reach of our method, including to low-resource settings. Nevertheless, we acknowledge that more analyses beyond these preliminary data will be needed to better define the experimental conditions required for near-perfect MoA recognition from BF images alone."

Reviewer #1 (Remarks on code availability)
The code has a README file and installation instructions. Frankly, I'm not in a position to install and run the code myself, so hopefully one of your other reviewers can do this check.

Reviewer #2 (Remarks to the Author):

The manuscript “Deep learning recognises antibiotic modes of action from brightfield images” presents a timely and innovative application of deep learning for identifying antibiotic Modes of Action (MOA) from label-free images. The potential to extract mechanistic information from a simple and accessible imaging modality is a notable achievement with clear relevance to the field of antibiotic discovery. This approach has several key strengths:

1. Early Mechanistic Insight: Traditional primary screens measure growth inhibition (e.g., optical density), providing a simple yes/no answer. This method can provide immediate classification of a hit's potential Mode of Action (MoA). This is valuable for hit prioritization, allowing researchers to focus on compounds with desired or novel mechanisms early on.
2. It helps quickly identify and discard compounds with known MoAs, tackling the major problem of antibiotic rediscovery. The ability to flag phenotypes that don't match the trained classes is a powerful feature. These ‘outlier’ hits are promising candidates for possessing genuinely new MoAs, which are urgently needed.
3. The fact that it can perform at sub-inhibitory concentrations is a key strength for primary screening. Many antibiotics induce clear morphological changes (e.g., filamentation, swelling) well below the concentration needed to kill the bacteria, providing a wider window for detection.
4. The ability of the pipeline to reliably work with label-free methods, such as brightfield imaging, makes it versatile and easy to apply to wide classes of bacterial targets.

We thank the reviewer for this summary and for highlighting these key strengths of our study.

While the approach is compelling, the manuscript would benefit from substantial revision to address concerns regarding methodological clarity, model validation, biological generalizability, and the interpretation of key results. Below are detailed points that the authors are requested to address to strengthen the manuscript.

Major Points for Revision

1. Investigating Model Interpretability: A significant opportunity to strengthen the manuscript lies in investigating the model's decision-making process. For a study linking images to biological phenomena, this is an essential validation step.
The manuscript would be substantially strengthened by an explainability analysis. It is strongly recommended that the authors employ established methods such as Gradient-weighted Class Activation Mapping (Grad-CAM) or saliency maps to visualize the image features driving classification.
This analysis would be critical for two reasons:
a. Biological Discovery: It could reveal the specific morphological phenotypes the model has learned, connecting its predictions back to tangible biology and potentially uncovering novel cellular responses.
b. Artifact Detection: Alternatively, the model might be “cheating” by focusing on experimental artifacts instead of true biological phenomena. For example, it could learn that a certain class of

antibiotic has lower solubility and tends to precipitate in the well. This may present subtle variations in the image background. This would make the model appear highly accurate in the lab but useless for predicting the MoA of a new, soluble compound, especially when the protocols are changed.
Therefore, analysis of image features driving the classifications would serve as a crucial control to help rule out the possibility that the model is relying on experimental artifacts (e.g., compound precipitate) rather than genuine cellular changes.

R2A:

We thank the reviewer for this comment, which closely aligns with a related comment from reviewer 1.

Encouraged by these comments, we have computed saliency maps using Grad-CAM, as recommended by both reviewers. We verified that these maps highlight bacterial cells (entirely or in part) as opposed to background areas, addressing the concern that the model might be cheating by focusing on variations in the image background. Moreover we used composite images to verify that Grad-CAM activations are stronger for the class corresponding to the actually used antibiotic, indicating that the learned features are MoA-specific.

However we note that Grad-CAM and related approaches have well-known limitations preventing these tools from providing a clear and specific description of the biological phenotypes extracted by the CNN. We hope that ongoing developments in interpretable AI will allow us to obtain such information in the future.

- ⇨ For more details about our Grad-CAM analyses and modifications to the manuscript, please see our response R1C above.

2. Addressing Biological Generalizability and Context
The claims of the study would be significantly broadened by demonstrating the approach's applicability beyond the current experimental scope.

a. To demonstrate broader utility, it would be beneficial to test the pipeline against a more diverse panel of clinically relevant pathogens. This could include a key Gram-positive organism (e.g., S. aureus) and other challenging Gram-negative pathogens (e.g., P. aeruginosa). Such experiments would be a powerful demonstration of the method's utility against the pathogens of greatest concern. Otherwise the conclusions of its utility remain limited to E. coli growing in rich media.

R2B: We thank the reviewer for this comment, which also aligns with a comment from reviewer 1.

Although systematically testing our approach on a wider range of bacterial pathogens is outside the scope of our paper, we have included new data and analyses on *Klebsiella pneumoniae,* another Gram-negative species of great concern, showing that our method's utility is not limited to *E. coli*. In addition we mention a preprint from two of our teams showing successful adaptation

of our methodology to *Corynebacterium glutamicum*, a Gram positive species whose smaller size and subtler phenotypes make analysis more challenging.

We believe these new data significantly strengthen the paper and hope they address the reviewer's comment.

⇨ For more details about these additions, please see our response R1A above.

b. To enhance clinical relevance, the study would be strengthened by validating the approach using bacteria grown in minimal media, ensuring the learned phenotypes are robust across different metabolic states. A phenotype observed in rich lab media may not be relevant or even present under more realistic conditions. Bacteria in a host environment often face nutrient limitations. Their metabolic state and physiological responses to antibiotics can differ dramatically in minimal media versus rich media. This limitation however is not unique to this study; several recent papers that propose such DL approaches for antibiotic discovery or susceptibility tests have relied on rich media based phenotyping, which makes the tasks easier but less relevant.

R2C:
We chose Mueller-Hinton (MHIIB) media for clinical microbiology reference, as it is the reference medium in clinical microbiological diagnosis and determination of MICs. We do agree with the reviewer that the bacterial phenotypes are dependent on growth media and that testing our approach in other growth media (including minimal media and media mimicking the host) would be an important extension of this work. However, we consider that investigating this lies beyond the scope of our present paper and will make it the topic of future work.

c. It is suggested that the method's application be framed more explicitly as a powerful secondary or parallel screen, acknowledging that a morphology-based screen may not detect all classes of antibacterial compounds. The primary goal of a primary screen is to find any compound with antibacterial activity. This model is trained to recognize specific morphological changes. A compound that kills bacteria without inducing a recognizable visual phenotype would be missed entirely. Therefore, it's better suited as a secondary screen or a parallel screen alongside a traditional growth inhibition assay. The authors should discuss these points in making this decision.

R2D:
We appreciate the reviewer's suggestion to more explicitly frame our method's applicability as a primary or secondary screen, and to discuss its complementarity to traditional growth inhibition assays. In line with the reviewer's comment, we propose that our method can play a role both as a secondary screen and as a parallel primary screen to growth inhibition assays. Specifically, we propose that our method can form the basis of a secondary screen to analyze compounds previously determined as active by a growth inhibition screen, where - after determination of an IC50 - it can help determine the MoA of these compounds or detect if they have a novel MoA. We also propose that our method can be used as a primary screen to identify compounds that elicit

phenotypes at sub-inhibitory concentrations (and would thus have been missed by growth inhibition screens). Such compounds may then be structurally optimized to more potent antibiotics (not to mention their value in disclosing and studying bacterial cell biology that goes beyond blunt killing).

⇨ This comment prompted us to include a new **Figure 8** that highlights these applications, along with the following sentences in the Discussion:

"We propose that our imaging-based approach provides a valuable complement to widely used growth inhibition screens, thanks to its ability to identify compounds at sub-inhibitory concentrations and its ability to either recognize MoA or detect novel MoAs (Fig. 8)."

"As sketched in Fig. 8, we propose that our CNN approach and the downstream analysis methods described here complements traditional growth-inhibition assays in a comprehensive phenotypic screening pipeline to discover novel classes of antibiotics to fight AMR."

3. Enhancing Methodological Clarity and Reproducibility
The manuscript in its current form lacks sufficient detail for the research community to fully reproduce the work and evaluate the findings. The following clarifications are requested:

a. Training and Data Processing: A more detailed description of the training loop and data augmentation pipeline is needed. It would be helpful to know, for instance, if augmentations were applied with a certain probability.

R2E:
We apologize for the lack of information in the submitted manuscript.

⇨ To address this request we added the relevant details about the training and data augmentation pipelines in the updated Methods sections 'Image preprocessing' and 'Deep learning model training' along with the **new Supplementary Table 3** (which details data preprocessing and data augmentation at training or testing time) and the **new Supplementary Table 4** (which lists model hyperparameters).

b. For the "minimal number of images" experiment, clarification on the image selection process is required. It is suggested that the authors elaborate on whether multiple models were trained on different random subsets to ensure the robustness of these results. e.g. how does the minimum required number of 7 images depend on the density and size of cells, and the magnification of the imaging system used here? Providing access to the relevant codes to the community would also be highly beneficial and critical for the community to test the applicability in wider contexts.

R2F:
For the results shown in Fig. 3g-i, models were indeed trained on random subsets of the images without consideration of cell density.

To address the reviewer's comment, we have repeated the analysis after splitting our training image data set into high, medium and low cellular density (evaluations were done on the exact same data set consisting of all images of the fourth replicate plate without considering differences in cell density). The average cell density between the high and low density data sets differed by a ratio of 1.11 to 1.96 depending on the compound, with an average of 1.35.
As shown in our new **Suppl. Fig. 10,** the number of training data required to achieve 95% of the plateau accuracy ranged from 6 to 8, i.e. a ratio of 1.33, almost equal to the average ratio of cell density. This is consistent with the intuitive expectation that the required amount of training data corresponds to a certain number of cells rather than a certain number of images.

- ⇨ We included these analyses in our **new Suppl. Fig. 10** and added a section 'Splitting images by bacterial density' to the Methods section with details about the estimation of cell density.
- ⇨ We also added the following sentence in the Results section 'CNN learns MoA-specific features from only seven images per condition' to mention this, after the sentence "We found that between seven (IC50) and 15 images (0.25×IC50) per condition (644 and 1,380 images in total) were sufficient to achieve the maximum accuracy.":

"This number ranged from six to eight (at 1×IC50) when training on the 30% images of highest or lowest cellular density, respectively, roughly consistent with an average 1.33-fold difference in average cell density (Supplementary Fig. 10)."

As for access to the code: our Github repository https://github.com/krentzd/ai4ab should allow anyone to apply our method to wider contexts. Please note that we also provide multiple pretrained models on HuggingFace at https://huggingface.co/krentzd/ai4ab.

- ⇨ We added a 'Code availability statement' to mention these repositories

c. A justification is requested for the decision to convert 16-bit images to 8-bit, as this step may discard useful data without offering a clear computational advantage. Since the model relies on morphology as well as texture from the BF images, it would be helpful to benchmark the performance across the raw and 8-bit formats.
Similarly, the rationale for downsizing images to 256x256 pixels should be explained. A comparison with models trained on larger tiles or crops would be informative to demonstrate that this loss of resolution does not negatively impact performance. Bacterial cells are small, and especially for poor media conditions or other smaller species, such as the relevant ones suggested below, the number of pixels per cell is a critical factor.

R2G:
We converted images from 16 bit to 8-bit formats and resized image patches from 500x500 to 256x256 for convenience, because the 8-bit 256x256 image format is suited to the EfficientNet architecture chosen in our work. However, we agree with the reviewer that these choices could potentially discard useful data, especially given the small size of bacteria. To address this concern, we retrained and tested our model on the original 16-bit data and assessed MoA recognition accuracy both at the level of individual fields of view and at the level of wells. As before, this was done 4 times, training on 3 replicate plates and testing on the fourth. As shown in our new **Supplementary Figure 9**, the MoA recognition accuracy does not significantly differ

when using 8-bit or 16-bit formats. Similarly, we retrained and tested models using the 500x500 images as input, without resizing. Again, as shown in **Supplementary Figure 9,** this change did not significantly affect MoA recognition accuracy**.** These new results underscore the robustness of our method to changes in bit depth and image resolution.

⇨ We included these new analyses in a new **Supplementary Figure 9** and added the following sentence to the Discussion paragraph starting with "At the technical level, it will be interesting to determine the generalizability of our method to changes in experimental conditions such as microscopy settings. ":

"We verified that MoA recognition accuracy is robust to changes in the image bit-depth (16-bit vs. 8-bit) and to a roughly 2-fold change in image resolution (500x500 vs 256x256 pixels) (Supplementary Fig. 9)."

d. ImageNet Pretrained Model Implementation: The manuscript would benefit from clarification on the image normalization strategy used, particularly regarding the discrepancy between the described (-1, 1) scaling and the standard ImageNet-specific mean/std normalization expected by pretrained PyTorch models.

R2H:
The choice to use a min-max normalisation between (-1,1) was made to reduce the model's sensitivity to cell density. Normalising by mean/std is not robust to variations in density, since images with higher cell density will have higher standard deviations.

⇨ We added the following sentence to the Methods section 'Image preprocessing':

"The latter normalization choice was preferred to the standard normalization (by subtraction of mean and division by standard deviation) to reduce the effect of changes in cell density. "

Finally, it is crucial for reproducibility that the authors specify precisely how the single-channel brightfield image was adapted for the three-channel pretrained model architecture.

R2I:
The model was adapted by explicitly changing the number of input channels in the first layer of the EfficientNet-B0 model. Please note that the parameters of the model pretrained on ImageNet were used only as initialization, i.e. that we retrained all parameters of the entire model to predict the combination of antibiotic and concentration.

⇨ We updated the Methods section 'Deep learning model training' as follows to explain this:

"To allow the model to accept images with different numbers of input channels, the first layer of the EfficientNet-B0 architecture was replaced with a convolutional layer (torch.nn.functional.Conv2d) with the appropriate number of input channels."

4. Resolving Inconsistencies and Clarifying Model Behaviour

The manuscript presents several results that require further investigation and explanation to build confidence in the model's robustness and the conclusions drawn.

Hold-out Plate Variability (Supp. Fig. 3): An investigation and discussion of the potential causes for the high variability and specific confusion patterns across hold-out plates are requested.

R2J:

We first would like to stress (as we did at the beginning of the Results section "Deep learning accurately recognises antibiotic mode of action from images") that the confusion matrices shown in **Supp. Fig. 3** pertain to the task of predicting both the identity of the antibiotic and its concentration from the images alone. This can be considered as a "pretext task" since in the evaluations of our model we actually ignore concentrations and (in most of our paper) pool antibiotics by MoA. Since antibiotics sharing same MoA are likely to have similar phenotypes and since two-fold changes in concentration are not always expected to yield strong changes in phenotypes, it would be very surprising if the model was able to determine both the precise identity and the concentration for each of the 22x4+1= 89 combinations of drug and concentration. In this light, we consider that these confusion matrices show rather strong recognition accuracy (~30 - 40 times better than random depending on the test plate). Nonetheless, we acknowledge that these confusion matrices exhibit a number of classification errors that are worth discussing.

First, we would like to point out that classification errors are more prevalent at lower concentrations. We acknowledge that this was not readily apparent in the confusion matrices of our former **Supp. Fig. 3** because treatments were ordered first by drug and then by concentration. Therefore we updated **Supp. Fig. 3** by changing the ordering to concentration first and drug second and added red boxes to highlight the four different concentrations. As an example, the confusion matrix for replicate 1 is shown below (**Fig. R0 below**). It is clear from the reordered confusion matrices that drug recognition accuracy increases with concentration, with poor recognition accuracy at 0.125xIC50 and high accuracy at 1xIC50. This observation is of course in line with our analysis of MoA recognition as function of concentration in the main text (section "Detecting drug phenotypes at subinhibitory concentrations") and Figure 4.

A closer inspection of classification errors at lower concentrations (up to 0.5xIC50) reveals several trends:

1) A first trend corresponds to errors in predicting concentration only, as reflected by the blue dashed diagonals in the confusion matrix below. For example, 27 FoV of Trimethoprim at 0.125xIC50 get misclassified as Trimethoprim at 0.25xIC50, and 49 FoV of Trimethoprim at 0.5xIC50 get misclassified as Trimethoprim at 1.0xIC50. Similarly, 30 FoV of Chloramphenicol at 0.25xIC50 get misclassified as Chloramphenicol at 0.5xIC50, etc. Such confusions are not surprising since (as mentioned above) 2-fold changes in drug concentration are not necessarily expected to yield strong phenotypic differences.
2) A second trend corresponds to several drugs that get misclassified as one specific (incorrect) drug, as reflected by errors along the columns highlighted by vertical dashed lines below. For example, many FOVs of several drugs at the lowest concentration (including Doxycyline, Polymyxin B and Colistin) get misclassified as controls (this is more

pronounced in replicates 2 and 3); likewise, many FOV of multiple drugs (including Meropenem, Clavulanate, and Relebactam) get misclassified as Trimethoprim in replicate 1; many FoV of multiple drugs get misclassified as Aztreonam or Cefepime at 0.5xIC50 in replicate 3, etc. Misclassifications as controls are not surprising for drugs at low concentrations, since the elicited phenotypes may be too weak for cells to be distinguishable from untreated cells. Confusions with one or a few specific drugs may seem less intuitive. However, we note that according to our analyses drugs of some MoA are more easily discernible from controls than other MoAs, as shown in Figure 4d,e. For example, PBP3 inhibitors (Aztreonam, Cefepime, Ceftriaxone) and membrane integrity inhibitors (Polymyxin B und Colistin) cannot be distinguished from controls for concentrations below 0.5 x IC50. Thus, these treatment conditions are essentially similar to controls and confusions of drugs with weak phenotypes with any of these conditions can be expected, in contrast to confusion with drugs that elicit strong phenotypes even at low concentrations, such as the PBP2 inhibitors

3) A third trend are confusions of drugs within the same MoA, either at the same concentration (relative to the IC50) or across different concentrations as reflected by errors within the black blocks on the diagonal or off-diagonal, respectively. These errors are of course entirely consistent with our main claim of accurate MoA recognition.

Inspection of the 89x89 confusion matrices in Supplementary Figure 3 shows that the vast majority of misclassifications are covered by one of these three patterns. We added the visual guides mentioned here to all confusion matrices (one for each replicate) to help their interpretation.

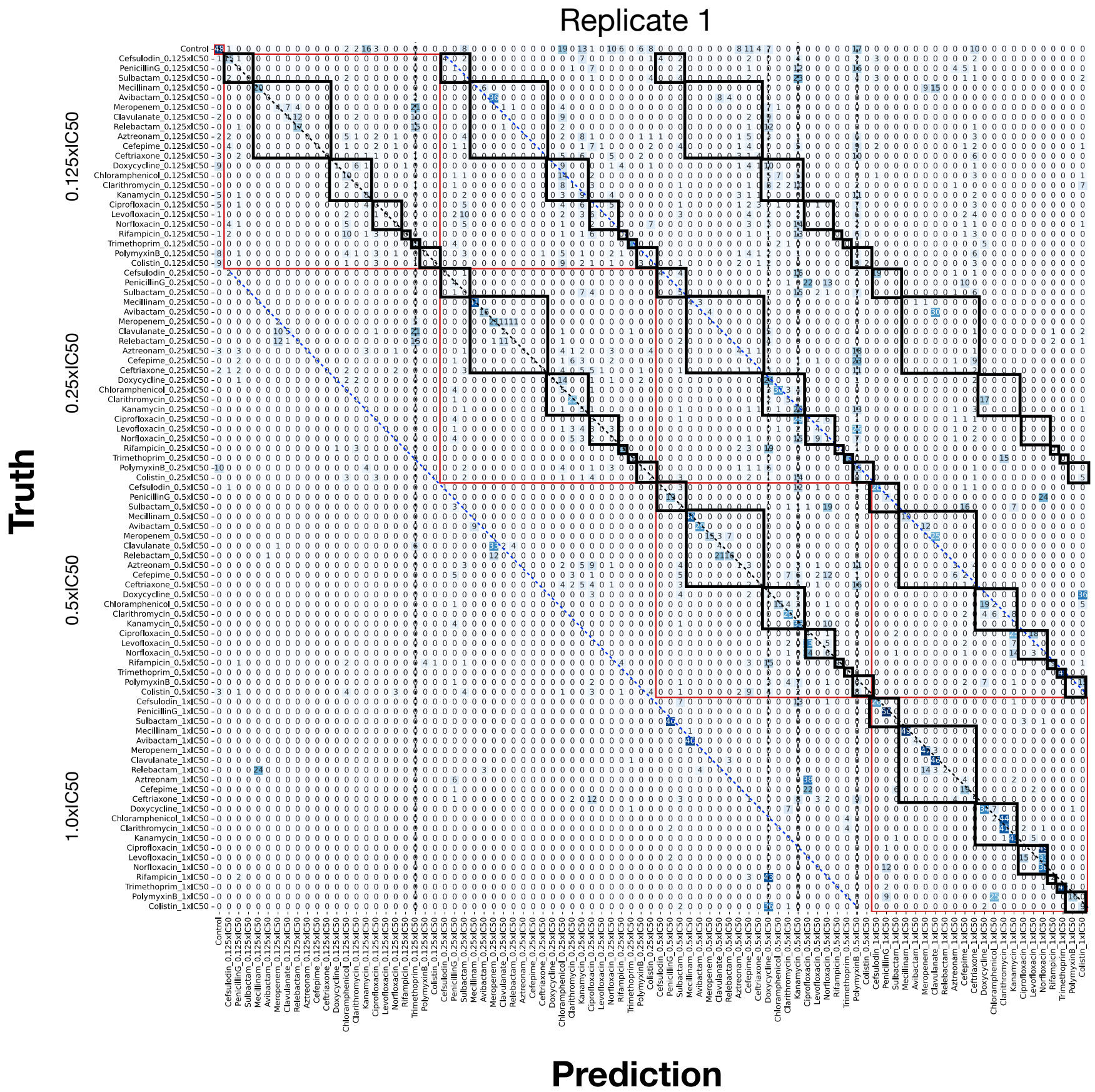

Figure R0: Confusion matrix of drug+concentration classification.
This confusion matrix shows classification results for a model trained on replicates 2-4 and tested on replicates 1, as shown in Suppl. Fig. 3. Red boxes show truth-prediction pairs within the same concentration levels (0.125, 0.25, 0.5 or 1x IC50). Black boxes show truth-prediction pairs for drugs belonging to the same MoA. Blue dashed diagonal lines correspond to confusions of concentration only. Vertical black lines highlight confusions of multiple drugs with the same drug.

However, we acknowledge that confusions are still present at 1x IC50 and merit discussions. We therefore analyzed the classification errors for drugs at IC50 and divided them in three categories:

1. Confusion between drugs of different MoAs (e.g. aztreonam misclassified as ciprofloxacin)
2. Confusion between drugs of the same MoA (e.g. aztreonam misclassified as cefepime)
3. Confusion of concentration only (e.g. cefsulodin at 1xIC50 confused with cefsulodin at 0.5xIC50)

Across the four plates, out of a total of 22x4=88 predictions at the level of individual drugs, we observed 36 classification errors. However, the majority of these misclassifications (20) were confusions between drugs sharing the same MoA (second category) and 2 additional errors were confusions of concentration only (third category). This left 14 cases of MoA confusions, i.e. an error rate of 14/89= 15.7%. None of these 14 MoA confusions were consistent across all four plates or even in three plates. Only 2 MoA confusions were present in more than one plate (i.e. two plates): (i) rifampicin was confused with doxycycline in plates 1 and 4, and (ii) colistin was confused with doxycyline in plates 1 and 4. Thus the majority of confusions (10/14) was restricted

to a single plate. In addition, we observe that both aztreonam and cefepime had wrong MoA classifications in plates 1 and 4, but they were classified in different MoAs (both drugs were classified as Ciprofloxacin in plate 1, whereas they were classified as Clarithromycin and Chloramphenicol, respectively, in plate 4). Thus overall there appeared to be no systematic pattern of MoA confusions across our 4 plates.

⇨ We modified Suppl. Fig. 3 to ease the interpretation of classification errors in the 89x89 confusion matrices by changing the order of treatment conditions and adding lines and MoA blocks as mentioned above. While we believe that the lengthy discussion of classification errors above may distract the reader from the main findings, we added the following sentence to the Results section 'Deep learning accurately recognises antibiotic mode of action from images' :

"Visual inspection of classification errors indicated that the majority of errors were confusions between drugs sharing the same MoA and that misclassifications were more frequent at lower concentrations, where predictions often defaulted to a small number of drugs or the negative control (Supplementary Fig. 3)."

Concentration-Dependent Accuracy: A deeper analysis is needed to explain why prediction accuracy is highly sensitive to concentration. It is suggested that interpretability tools could be used to show what morphological features are gained at higher concentrations.

R2K:
The observed sensitivity of prediction accuracy to concentration arises because for most of the considered antibiotics the phenotypes deviate much more from the control at higher concentrations then at low concentrations, as illustrated by the examples below (**Fig. R1**), where 1x IC50 levels of trimethoprim or norfloxacin lead to much more elongated bacteria than low concentrations, e.g. 0.125xIC50.

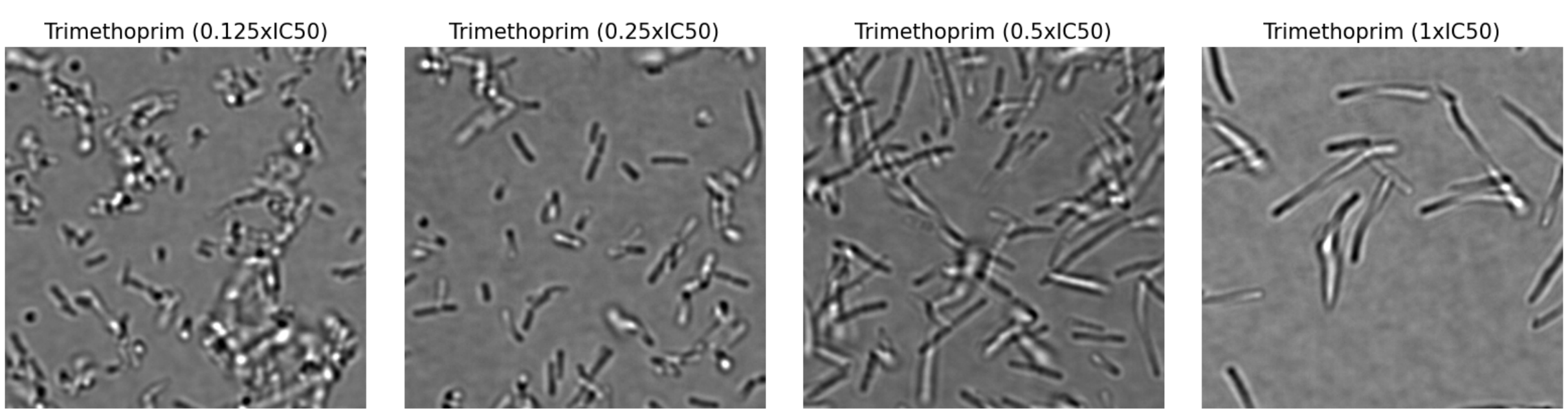

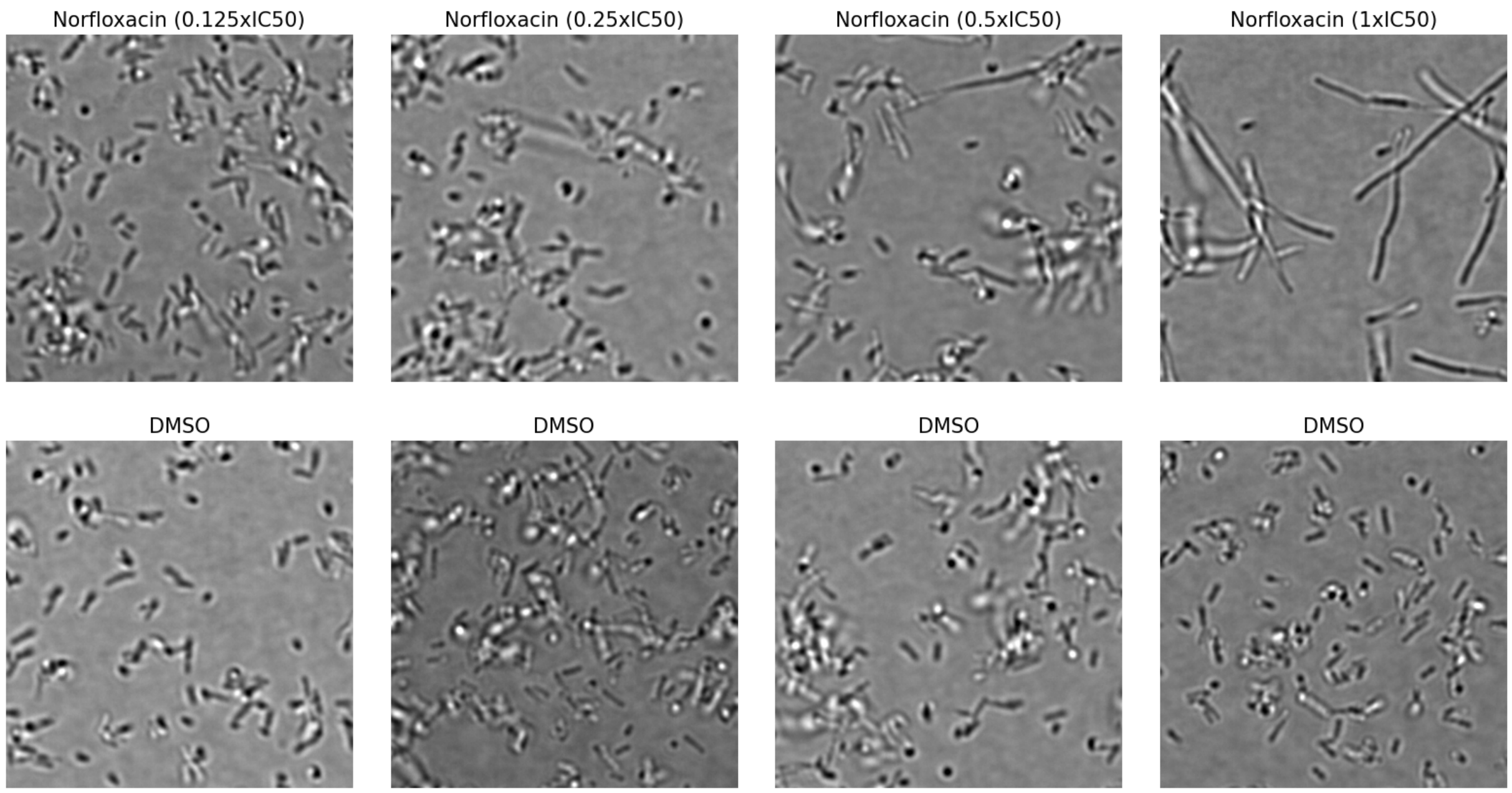

Figure R1: Concentration-dependent phenotypes
Examples images of *E coli* treated with trimethoprim and norfloxacin as function of concentration (from 0.125xIC50 to 1xIC50) and controls (DMSO).

Encouraged by the reviewer we attempted a Grad-CAM interpretability analysis in the hope of identifying concentration-dependent features in the images. For this purpose, we created composite images of cells treated by low and high concentrations of antibiotics (**Fig. R2**), along the same lines as the Grad-CAM analyses reported in R1C and R2A above. This analysis showed that, as expected, the Grad-CAMs highlight the image half corresponding to the targeted concentration class more so than the other image half. This is consistent with the model having learned concentration-dependent image features. However, this analysis did not provide clear additional insights into concentration-specific morphological features compared to direct inspection of the images themselves (**Fig. R1**). We therefore did not include these additional data in the paper. As mentioned above (see R1C), we included a brief passage about interpretability in the perspectives of the Discussion.

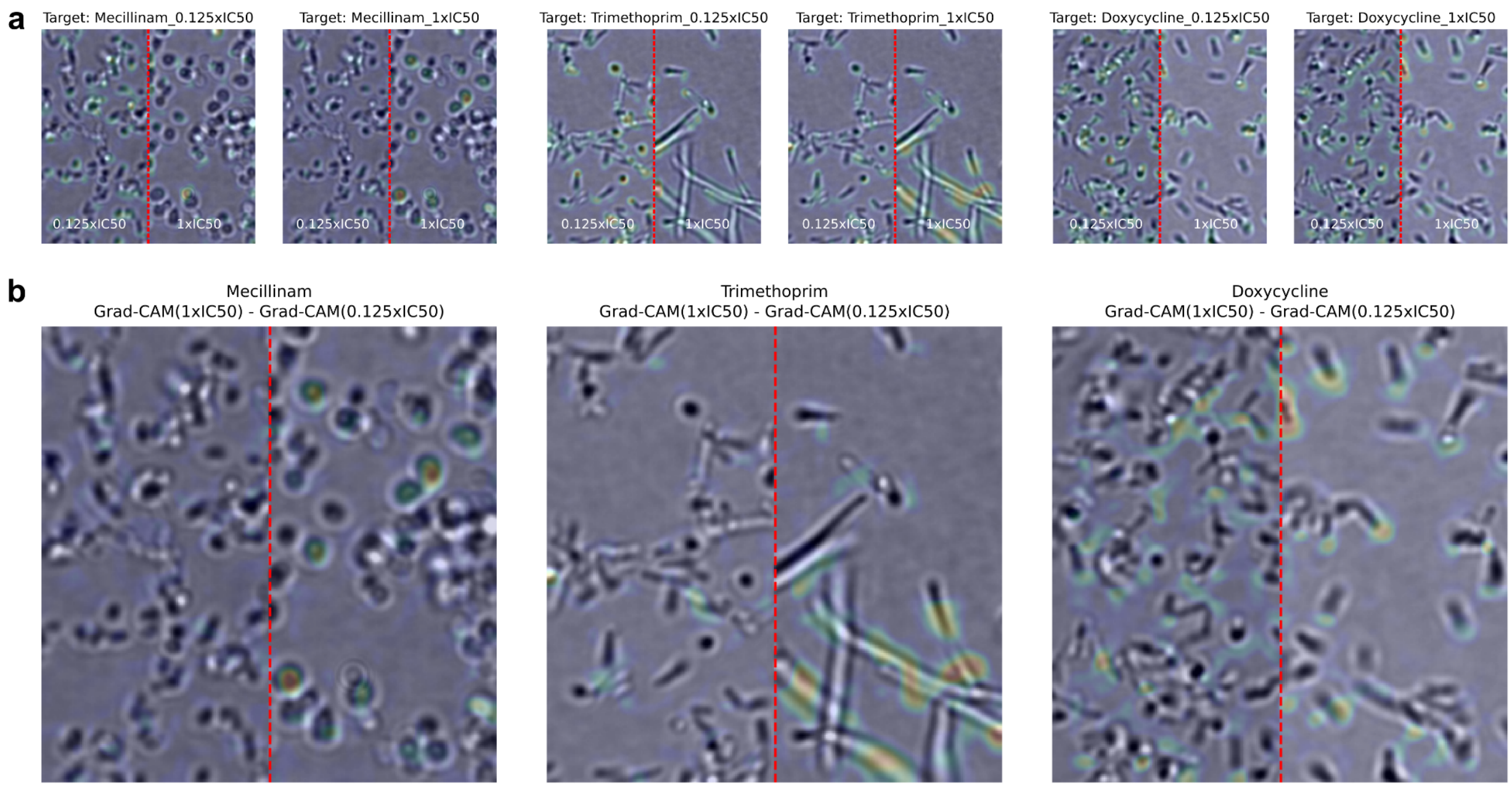

Figure R2: Grad-CAM computed between low and high drug concentrations.
a) Shown are composite brightfield images where the left half is from a low concentration treatment (0.125xIC50) and the right half is from a high concentration treatment (1xIC50) of Mecillinam (left), Trimethoprim (center) or Doxycycline (right). The superposed heatmaps correspond to the Grad-CAMs for the corresponding drug at the low or high concentration, as indicated. **b**) same as a, but here the superposed heat maps show the differential Grad-CAMs, ie the differences between the Grad-CAM for the high concentration and the Grad-CAM for the low concentration.

Direct MOA Prediction: It would be a valuable benchmark if the authors trained and evaluated a model that directly predicts the MOA class, rather than relying on a post-hoc assignment after antibiotic-level prediction. This would provide a more direct test of the model's ability to learn MOA-specific features.

R2L: We trained on antibiotics and concentrations as labels (rather than directly on MoA labels) because this circumvents the need to assign antibiotics to specific MoAs at training time. MoA assignment of individual antibiotics is not entirely straightforward and may depend on the exact definition of the MoA, and on potentially incomplete or inaccurate annotations in the literature. These difficulties are entirely avoided when training on antibiotics and concentrations. Please see our response R1B above.

Nonetheless, we followed the reviewer's suggestion and retrained our model on MoA labels. This did not lead to a significant change in the classification accuracy, as shown in our **new Supplementary Figure 9** (compare 'on moa' to 'BF'), indicating that our choice of training label does not negatively affect performance.

LOCO vs. Cosine Similarity: A discussion is needed to clarify the apparent discrepancies between the k-NN classification in the LOCO analysis and the cosine similarity of feature vectors.

R2M: We are not sure to understand this point. Please note that MoA assignment in the LOCO analysis is based on a majority vote of the 150 nearest embeddings computed from individual images (FOV), whereas the cosine similarity matrix applies to the median of all embeddings from a single well. In addition the embeddings in the LOCO analysis of Fig. 5 are based on a somewhat different model than that used in Fig. 3 since one compound was left out from the training data in the former but not in the latter. Nevertheless we do not see a clear discrepancy here since the k-NN MoA assignments in Fig. 5 are in fact consistent with the cosine similarity of Fig. 3f.

Model Performance and Implementation Choices: An explanation is requested for why the brightfield-only model achieves the highest per-well accuracy but the lowest per-FOV accuracy.

R2N:
We thank the reviewer for pointing this out. In principle, the per-well accuracy does not necessarily rank like the per-FOV accuracy because the per-well predictions are computed from a majority vote, ie a maximum statistics, while the per-FOV accuracies are not. However we double checked this analysis and found that there was a mistake in Suppl. Fig. 5 (due to an inconsistent choice of checkpoints across the models). We apologize for this error. After correction, it turns out that the brightfield-only model no longer has the lowest per-FOV accuracy, as shown below.

Old Suppl. Fig .5:

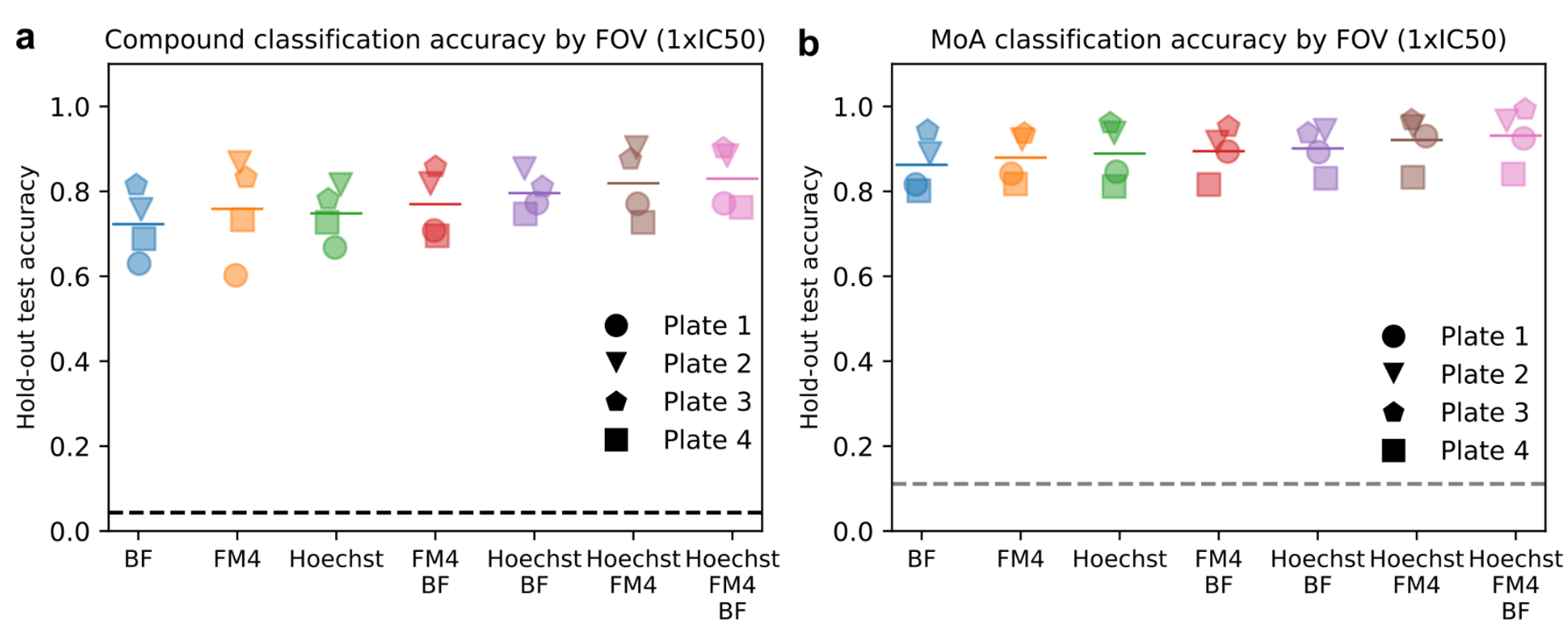

Corrected Suppl. Fig. 5:

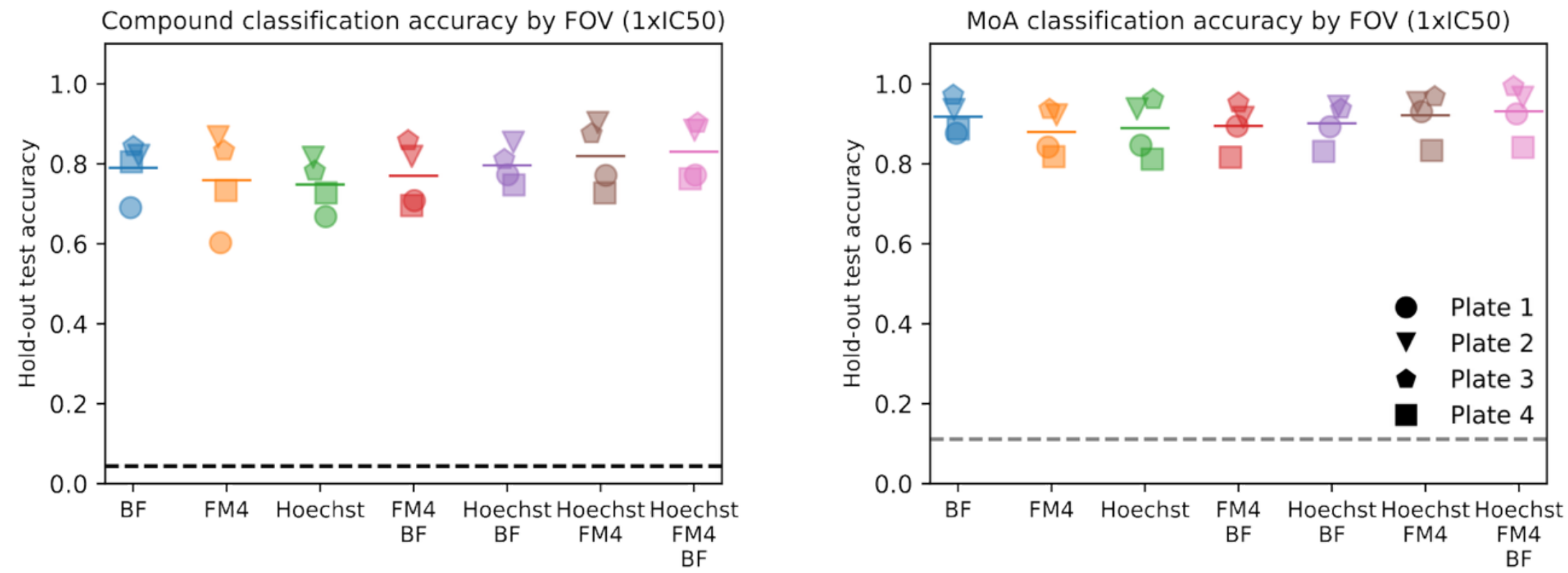

Please note that in the revised manuscript we included statistical tests to assess if the differences in accuracies are significant between any of the considered combinations of input channels.

⇨ We corrected **Suppl. Fig. 5** and added statistical tests in the revised manuscript.

Furthermore, a justification for using k-NN for classification instead of the model's output logits should be provided.

R2O:
We agree that using the logits may have been a more natural choice. However, we empirically observed that the classification accuracy using the k-NN is slightly better, as shown in Fig. 5c. This is why we turned to the k-NN classification in subsequent analyses.

Minor Points & Suggestions

i. The manuscript could be enhanced by a brief discussion on the potential of a multi-task learning approach (e.g., predicting compound and concentration simultaneously).

R2P:
Our model is trained to predict both compound and concentration, treating the combination of both as a single class. Although we showed that this approach is highly effective (and has benefits over alternatives such as training on MoA), we agree that other approaches may be considered and could potentially lead to further improvements. We therefore added the following sentence to the second to last paragraph of the Discussion (right after the discussion of explainability:

"A related limitation is our treatment of concentration labels in the same manner as compound labels. We speculate that better performance on compound classification might be achievable by reframing the prediction of concentration as a regression task or by enforcing the learning of features robust to changes in concentration."

ii. To better contextualize the data requirements, it would be informative to see how model performance is affected when data augmentation is turned off.

R2Q:
We thank the reviewer for this comment and have re-trained the models without data augmentation. Models without data augmentation rapidly overfit, evidenced by the discrepancies between training and validation loss (Fig. R3, right). Despite this, test accuracies are comparable when training with or without data augmentation for 100 epochs.

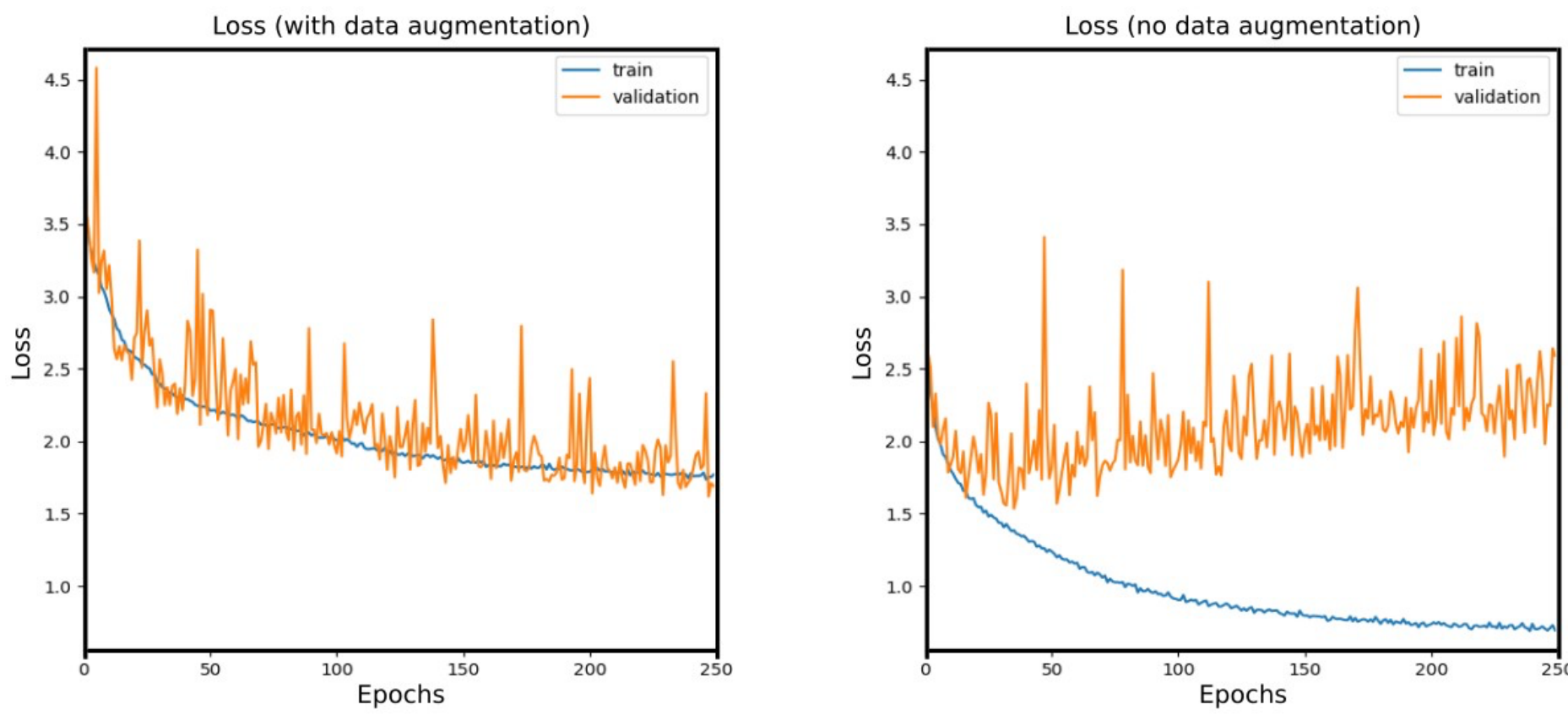

Figure R3: Effect of data augmentation.
Training and validation loss curves are shown as function of epoch in blue and orange, respectively. Left: with data augmentation. Right: without data augmentation.

iii. For the Leave-One-MoA-Out (LOMO) tests, clarification is needed on whether the training data was rebalanced.

R2R:
For the LOMO analysis, models were trained to predict the combination of antibiotic and concentration only. Hence the number of training images was the same for each class (50x3=150 across the three replicate plates) and no rebalancing was needed.

Please note that for the new data from HZI Braunschweig, we used a balanced cross-entropy loss function to account for the class imbalance between treatment conditions and DMSO negative controls.

iv. A higher-resolution version of Supplementary Figure 3 and a more intuitive color scheme or direct labeling for Figure 2e are requested to improve readability.

R2S: We apologize for the low resolution of Suppl. Fig. 3 and the limited readability of Fig. 2e.

- ⇨ We replaced Suppl. Fig. 3 with a higher resolution version and added labels to improve readability of Fig. 2e.

Reviewer #2 (Remarks on code availability)
The code was not linked in the main manuscirpt. We missed it until we accessed this review submission page. It will be helpful to include to the github link for code in the main paper and the requested details in the review should be included in the methods details of the paper.

R2T:
We apologize for having omitted a link to the source code in the submitted manuscript. We have now added a Code availability statement with the link to the Github repository corresponding to our code (https://github.com/krentzd/ai4ab) as well as a link to a HuggingFace page from which multiple trained models can be downloaded.

As for the details requested in the review, we included them in our updated Methods section as indicated in our responses above.

Reviewer #3 (Remarks to the Author)
I co-reviewed this manuscript with one of the reviewers who provided the listed reports. This is part of the Nature Communications initiative to facilitate training in peer review and to provide appropriate recognition for Early Career Researchers who co-review manuscripts.

Response to reviewer comments

Reviewer #2 (Remarks to the Author):
The authors have made substantial revisions to the manuscript, and many of the major conceptual and methodological concerns raised in the previous round have been addressed. I appreciate the addition of Grad-CAM analyses and composite-image controls, the new K. pneumoniae experiments, the bit-depth and image-resizing comparisons, the HZI/Braunschweig replication experiments, the expanded description of preprocessing and augmentation, the direct MoA-label training comparison, the analysis of cell-density effects on the training-image requirement, and the correction of the previous inconsistency in the channel-comparison analysis. These additions substantially strengthen the manuscript and improve the transparency of the work.

I also appreciate that the authors now more explicitly discuss limitations, including the restricted species scope, the remaining limitations of Grad-CAM for biological interpretation, the effect of concentration on phenotype strength, and the limitations of discrete MoA labels. Overall, the revised manuscript is considerably stronger than the original submission.

R0: We thank the reviewer for this assessment and the very careful analysis of our revised paper.

However after checking the linked code, I could not find the code for the Grad-CAM analyses or much of the supplementary analyses, K. pneumoniae, HZI replication or bit-depth/resizing comparisons, and only see notebooks for figures 2 to 6 at the GitHub link. Were these the only codes intended to be shared?

R1: We apologise for this oversight and have now uploaded all of the relevant code to the GitHub repository:

- *K. pneumoniae* analysis (corresponding to Figures 7b,c): https://github.com/krentzd/ai4ab/blob/main/analysis/Figure_7.ipynb
- Grad-CAM saliency maps (corresponding to Supp. Figures 7 and 8): https://github.com/krentzd/ai4ab/tree/main/model/GradCAM
- HZI replication replication analysis (corresponding to Supp. Figure 18): https://github.com/krentzd/ai4ab/tree/main/analysis/Supplement/HZI_replication_analysis
- Bit-depth/resizing and other comparisons (corresponding to Supp. Fig. 9): https://github.com/krentzd/ai4ab/tree/main/analysis/Supplement/Benchmarking
- Clustering of K. pneumoniae data based on E. coli features (corresponding to Supp. Fig. 15): https://github.com/krentzd/ai4ab/tree/main/analysis/Supplement/K_pneumoniae_clustering_with_E_coli_model
- Analysis of the influence of cell density (corresponding to Supp Fig. 10): https://github.com/krentzd/ai4ab/tree/main/analysis/Supplement/Minimum_required_training_images_by_cell_density

While browsing the code I came across some points that would benefit from some clarification: During training the validation uses the same training augmentation (get_train_transforms) which will apply random flips, rotations etc. Since checkpoints are selected based on validation loss, the model selection is stochastic. Typically validation should use deterministic test/eval transforms. The authors should justify this choice or demonstrate that it does not affect checkpoint selection or reported performance.

R2: To address this comment, we retrained our model and tracked the validation loss using deterministic augmentations and three randomly augmented validation sets. Please note that we used exactly the same validation images and only changed the applied transforms. We selected the checkpoint with the lowest validation loss using each of the tracked losses and found that the performance is not affected when applying random data augmentation to the validation dataset (see **Figure RF1** below).

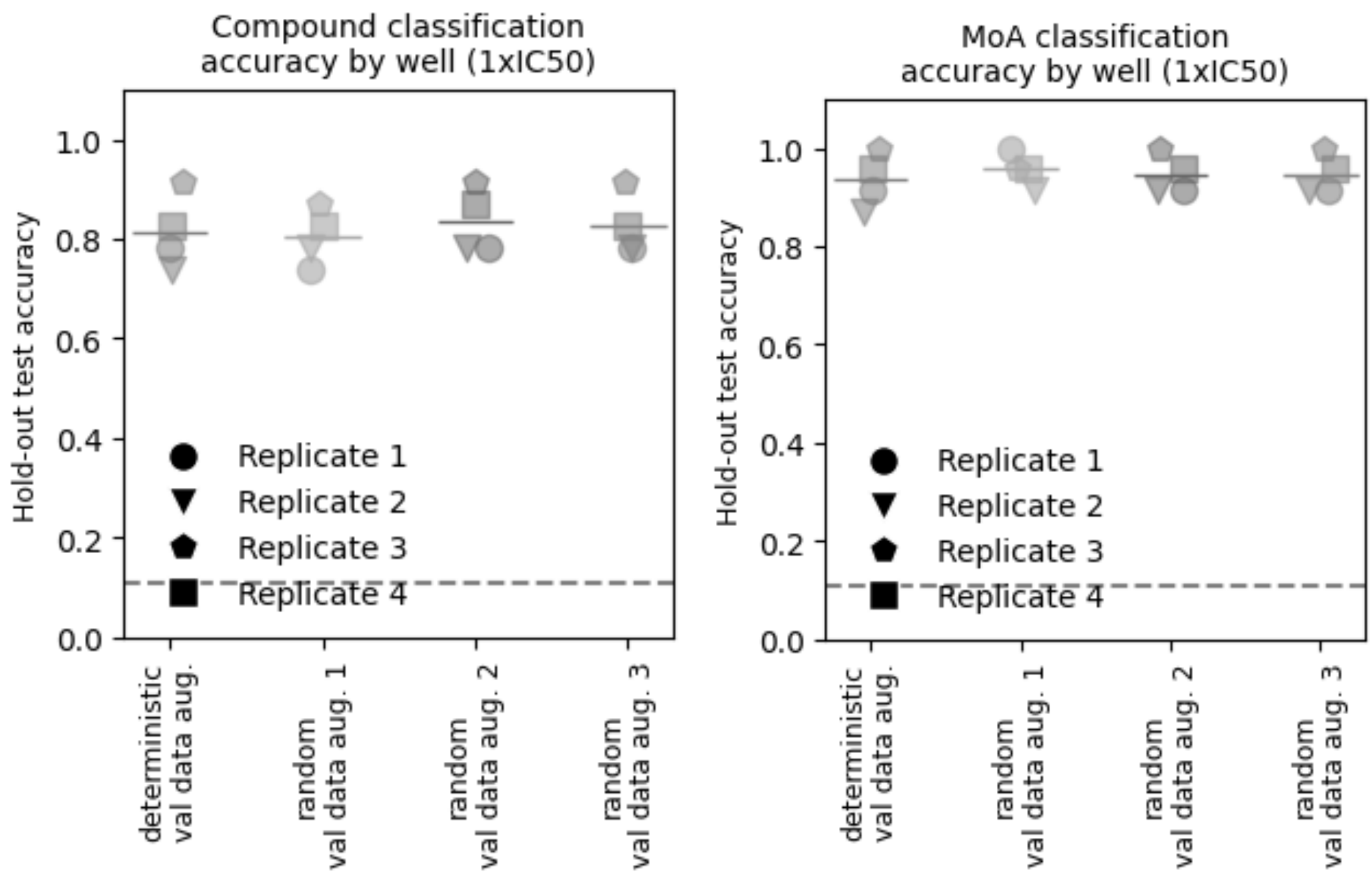

Figure RF1: Benchmarking classification performance with checkpoints selected from loss curves of deterministically vs randomly augmented validation datasets. Well-level classification accuracies for compound (left) or MoA (right) prediction. Each symbol corresponds to test data from one replicate (plate), with the three other replicates (plates) being used for training. The validation loss was computed using the same validation images but with four different data transformations applied: deterministic data transformations (as used for the test images) and three with random augmentations (as used for the training images). Horizontal lines show the median classification accuracy. Dashed lines correspond to random classification.

I am confused by the term "well-level". Can the authors please clarify what they mean? In utils.py, _compute_conditional_max_accuracy() loops over set(cond_labels), not over well identifiers. Am I right that since each compound and dose is one well, grouping by cond_label is effectively grouping over individual wells? Therefore, if use_moa_labels is set to True, _compute_conditional_moa_max_accuracy() wil be called and this first maps the FOV labels to the MoA labels, and then loops over set(moa_cond_labels). Does this not mean that the MoA majority vote is over all FOVs sharing the same true MoA, and not over each well? I note that the revised manuscript gives a classification accuracy at the well-level for one of the plates of 88.9%, which is 8/9. The authors should clarify the denominator for the reported "well-level" MoA accuracies and, if necessary, recompute true per-well MoA accuracy. I believe the "seven images per treatment condition" result also inherits this issue. The authors should verify whether the "seven images per condition" result is robust when evaluated using true per-well or per-condition MoA predictions.

R3: We very much thank the reviewer for this comment, which identified an inconsistency between the term "well-level" and the MoA-classification accuracies we reported. As pointed out by the reviewer, the reported accuracies actually corresponded to the 9x9 MoA confusion matrices (as shown e.g. in Fig. 2c and 3e), which involve a pooling of the compounds by MoA and hence a denominator of 9. Although this pooling is relevant for the ground truth compounds, it is not relevant for the tested compounds, since MoA groupings cannot be assumed a priori. Thus, the practically relevant accuracies should actually have a denominator of 23 (the number of antibiotics + control).
To address this, we have corrected the code in question and revised all of the reported well-level MoA accuracies throughout the manuscript, and updated **Fig. 3c** and **Supp. Fig. 9**. Note that as a result of this correction the MoA prediction accuracy from BF data is no longer 9/9=100% for all replicates, but is still excellent (median: 22/23=95.6%). We also corrected the mean well-level MoA accuracies for the HZI analysis and have updated **Supp. Fig. 18** accordingly. We also updated the analysis for estimating the minimum number of required images, which increased slightly from 7 to 8 at IC50 and we have updated **Fig. 3g-i** accordingly. We thank again the reviewer for having caught this important point.

I am also confused by the scaling of the feature vectors in the LOMO analysis. LOMOPlotter.plot_roc_curves fits a StandardScaler to feat_vecs_ which is the full feature set, is it not? This seems to be done before fitting the local outlier factor model. Surely for novelty detection scaling and other preprocessing should be fitted on the training/reference distribution only, and then applied to the held-out candidate compounds? The authors should check whether this affects the reported ROC values. The same issue appears in plot_novelty_score() and related LOMO visualisation code.

The same is true for plot_roc_curve (singular) for Figure 4. Cosine distance is not translation invariant so centering the data on feat_vecs could influence the scoring space. I do not know if this will change the ROC values at all but the authors should check their implementation because this seems like a potential data leakage issue. The authors should either recompute

the ROC curves without this centring step, or fit any preprocessing only on an appropriate reference distribution, such as training-set controls, before applying it to held-out samples.

R4: We thank the reviewer for having identified this potential data leakage issue. We of course agree that the scaling of feature vectors should be based exclusively on the training data. We have therefore corrected the calculation of the scaling transform such that it only relies on the feature vectors present in the training data and removed the centring step. This resulted in slight changes of the reported novelty detection scores, ROC curves (in some cases imperceptible) and associated AUC values (see **Figure RF2** below). We have updated **Fig. 6d** and **6e** accordingly.

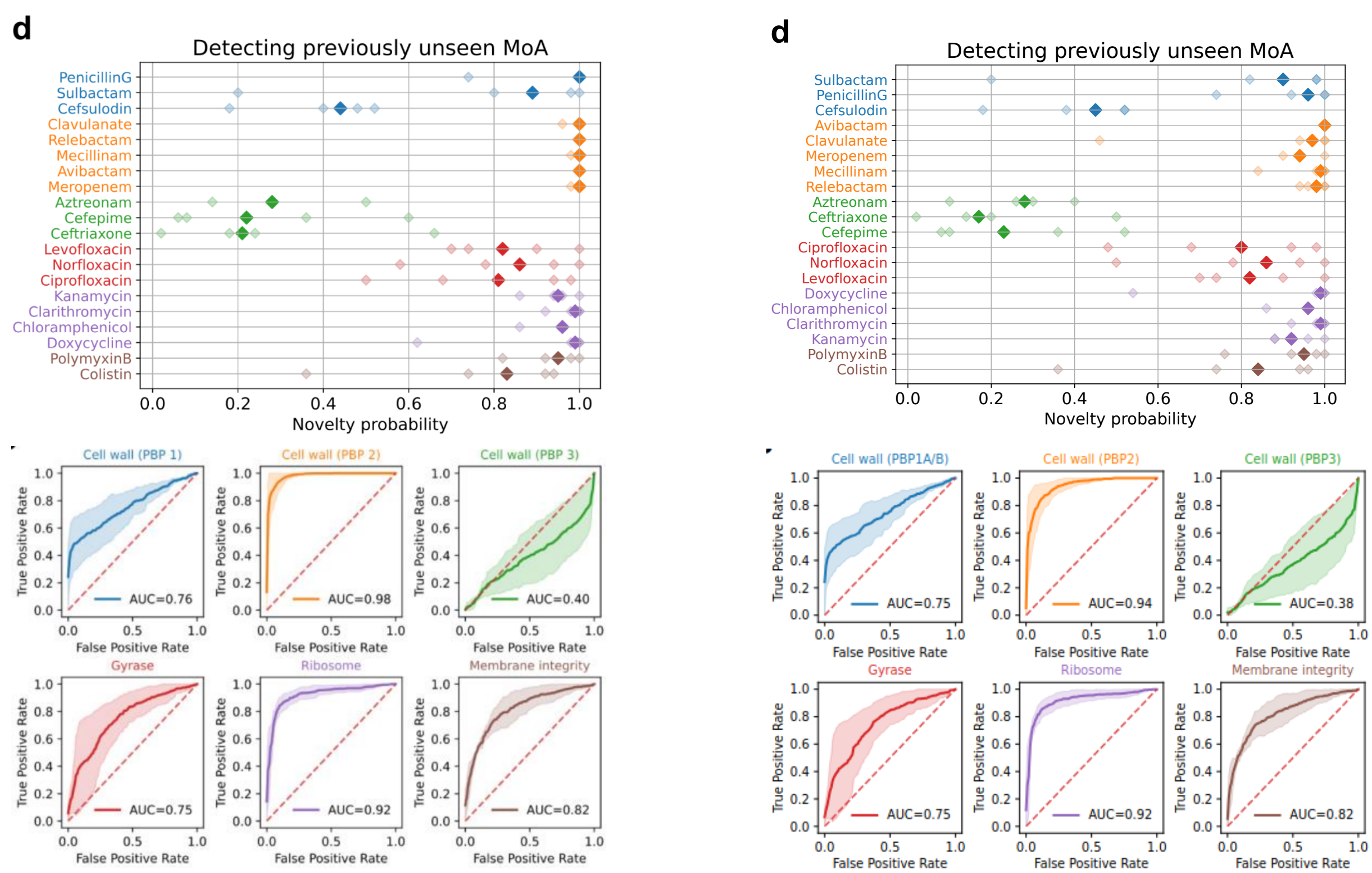

Figure RF2: Original and updated probability scores and ROC curves for MoA novelty detection.
Left: original Figures 6d (top) and 6e (bottom), before correction. Right: after correction of the scaling procedure.

Two more final points:
The authors might want to consider citing similar work on DL-based novelty detection that was published recently (after their preprint): https://doi.org/10.3389/fmicb.2025.1640252. The authors might want to soften some claims about novelty detection in the introduction.

R5: Sedaghat et al. is indeed a highly related paper that was published after submission of our study. This paper uses holographic microscopy and deep learning to determine antibiotic MoA and also includes an analysis of MoA novelty detection based on a few test compounds. We now cite it in two places of the Discussion:

1) In the paragraph "First, we establish that DL can extract both MoA- and compound-specific features … Our finding that fluorescent labelling of bacterial membranes and DNA is redundant with BF images is consistent with studies showing that DL models can be trained to generate realistic fluorescent labelling or coloration (virtual staining) *in silico* from label-free images in eukaryotes[20] and in bacteria respectively, as illustrated by a recent report that Gram-staining can be predicted from darkfield images of *E. coli* and *Listeria innocua*[21]. They are also in line with a recent related study demonstrating MoA recognition in unlabelled *E. coli* using time-resolved holographic microscopy[22]. " (added sentence underlined)
2) "Fifth, we have addressed the critical task of determining if a novel compound also has a novel MoA[22]. " (added reference)

We note that the novelty analysis of Sedaghat et al. was done on three compounds at concentrations well above MIC, whereas our LOMO analysis was done systematically on the majority of the antibiotics in our data set and used sub-MIC concentrations (IC50). We therefore modified our sentence in the Introduction concerning MoA novelty as follows:

> "Second, it remains to be determined to what extent unseen compounds can be assigned to their correct MoA and, critically, whether compounds with novel MoAs can be detected, especially at sub-MIC concentrations." (added words underlined)

Furthermore, our revised abstract (now shortened to 200 words as requested) reflects these aspects in the sentence:

> "Imaging-based profiling of drug-treated bacteria can inform on MoA, but current approaches generally require fluorescent labelling and/or inhibitory concentrations and it remains unclear whether compounds with novel MoAs can be robustly detected"

The Zoffman et al paper in Scientific Reports, cited by the authors defines a concept known as the "lowest observed effective dose" (LOED) and finds that they can detect changes in certain features at an MIC/LOED ratio of 3.9x. They perform analysis at 2x-4x LOED, which is in the sub-MIC range, although I grant they likely don't go as low as this manuscript. However the authors might want to soften the claims in the introduction.

R6) We acknowledge this and modified the corresponding statement in the introduction as follows:

> "First, it is unclear if antibiotic compounds can be identified from images at concentrations well below MIC, which would greatly improve the sensitivity of large-scale screens that typically test thousands of compounds at only one or two fixed concentrations."

Reviewer #2 (Remarks on code availability):
NA

Other changes not requested by the reviewer:

R7) We realized that our Grad-CAM implementation used ReLu functions after the activations, which were not used in the original Grad-CAM paper, and therefore removed them. As a result, our counterfactual analysis improved quantitatively (i.e. the class-dependent activations are higher for the correct class and lower for the incorrect classes), without changing the qualitative conclusions. We updated the relevant supplementary figures (**Supp. Figs. 7** and **8**).

R8) While assembling the data from the HZI replication experiments for upload to Bioimage Archive, we noticed that images in four of the wells were acquired at a different magnification, which was not accounted for in the analyses reported in **Supp. Fig. 18**. We have therefore repeated the analysis after removing these four wells from the data and updated **Supp. Fig. 18** and the corresponding text accordingly. The results are only marginally different (e.g. well-level accuracies on MoA are now 81.6% instead of 85%) and do not change our conclusions.
